# Configuration and Delivery of Primary Care in Rural and Urban Settings

**DOI:** 10.1007/s11606-022-07472-x

**Published:** 2022-03-09

**Authors:** Taressa K. Fraze, Valerie A. Lewis, Andrew Wood, Helen Newton, Carrie H. Colla

**Affiliations:** 1 Department of Family and Community Medicine, Healthforce Center, Philip R. Lee Institute for Health Policy Studies, University of California, San Francisco, CA USA; 2 Department of Health Policy and Management, Gillings School of Global Public Health, University of North Carolina at Chapel Hill, Chapel Hill, NC USA; 3 The Dartmouth Institute for Health Policy and Clinical Practice, Geisel School of Medicine, Dartmouth College, Hanover, NH USA; 4 School of Public Health, Yale University, New Haven, CT USA

**Keywords:** rural health, primary care, outpatient utilization, inpatient utilization, practices, access to care

## Abstract

**Background:**

There are concerns about the capacity of rural primary care due to potential workforce shortages and patients with disproportionately more clinical and socioeconomic risks. Little research examines the configuration and delivery of primary care along the spectrum of rurality.

**Objective:**

Compare structure, capabilities, and payment reform participation of isolated, small town, micropolitan, and metropolitan physician practices, and the characteristics and utilization of their Medicare beneficiaries.

**Design:**

Observational study of practices defined using IQVIA OneKey, 2017 Medicare claims, and, for a subset, the National Survey of Healthcare Organizations and Systems (response rate=47%).

**Participants:**

A total of 27,716,967 beneficiaries with qualifying visits who were assigned to practices.

**Main Measures:**

We characterized practices’ structure, capabilities, and payment reform participation and measured beneficiary utilization by rurality.

**Key Results:**

Rural practices were smaller, more primary care dominant, and system-owned, and had more beneficiaries per practice. Beneficiaries in rural practices were more likely to be from high-poverty areas and disabled. There were few differences in patterns of outpatient utilization and practices’ care delivery capabilities. Isolated and micropolitan practices reported less engagement in quality-focused payment programs than metropolitan practices. Beneficiaries cared for in more rural settings received fewer recommended mammograms and had higher overall and condition-specific readmissions. Fewer beneficiaries with diabetes in rural practices had an eye exam. Most isolated rural beneficiaries traveled to more urban communities for care.

**Conclusions:**

While most isolated Medicare beneficiaries traveled to more urban practices for outpatient care, those receiving care in rural practices had similar outpatient and inpatient utilization to urban counterparts except for readmissions and quality metrics that rely on services outside of primary care. Rural practices reported similar care capabilities to urban practices, suggesting that despite differences in workforce and demographics, rural patterns of primary care delivery are comparable to urban.

**Supplementary Information:**

The online version contains supplementary material available at 10.1007/s11606-022-07472-x.

## INTRODUCTION

Primary care aims to deliver care that is a patients’ first contact, comprehensive, and coordinated, and offers continuity between clinicians and patients.^1–5^ Pressure on primary care clinicians to assume responsibility for population health, outcomes, and costs has increased over the past decade.^6–8^ Despite the recognition that high-quality primary care is an important determinant of health, problems persist: access to primary care is uneven,^9^ supply of primary care physicians is decreasing,^10,11^ patients are increasingly complex,^12–14^ and primary care is under-resourced. There are just 3 primary care physicians per 10,000 people in non-metropolitan areas compared with 8 in metropolitan areas.^15^ The workload for rural primary care clinicians is also greater because they typically deliver a wider range of services despite the reduced workforce.^16–18^

Policymakers have committed to addressing challenges around access to care in rural settings by tailoring existing payment and delivery policies with a rural “lens.”^19^ At the same time, provider organizations, including the American Medical Association, advocate policies aimed at increasing the supply of physicians in rural settings.^20^ Despite the increased focus and commitment to supporting rural primary care, we know strikingly little about how primary care is configured and delivered across rural settings. There are clear cultural and workforce differences across rural settings, yet research typically combines isolated, small towns, and suburban areas into a single rural category which may mask important differences. While there are fears about the ability of rural primary care to deliver coordinated and comprehensive care,^18,21^ there are few national studies examining care delivery capabilities of primary care practices across settings. Patients regularly report challenges to accessing primary care in rural settings,^22,23^ yet there is little research comparing the patterns of outpatient utilization.

In this paper, we seek to fill these gaps by exploring the configuration of primary care practices to understand the landscape of primary care delivery.

## METHODS

We characterized physician practices and their Medicare beneficiaries across isolated, small town, micropolitan, and metropolitan settings in 2017.

### Study Population and Data Sources

IQVIA’s OneKey database, which operationalizes the relationships between individual clinicians and practices, was used to define and characterize physician practices. OneKey nationally characterizes physician practices in terms of ownership, composition, number of physicians, and types of clinicians.^24^ OneKey is a proprietary database that relies on primary data collection efforts, the American Medical Association’s Physician Masterfile, and publicly available sources. The study population retains all IQVIA OneKey physician practices with assigned Medicare beneficiaries, regardless of size or specialty.

We used 2017 Medicare fee-for-service claims to understand more about the beneficiaries using each physician practice, and their travel and utilization patterns. We included beneficiaries with full, continuous part A and B coverage, 18–99 years of age, and residing in one of the 50 US states or Washington, DC. We excluded beneficiaries who turned 65 during 2017 due to inability to determine claims history. We adapted methods used by the Centers for Medicare and Medicaid Services’ Medicare Shared Savings Program to assign beneficiaries to the physician practice from IQVIA where they received the plurality of their outpatient evaluation and management visits. We prioritized visits to primary care clinicians over specialist clinicians in this assignment such that beneficiaries were only assigned to a specialist if they had no visits from a primary care clinician (family medicine, general internal medicine, geriatrics, preventive medicine, nurse practitioner, clinical nurse specialist, physician assistant). Only beneficiaries cared for by one of the OneKey identified physician practices were retained in our study population (86.5% of beneficiaries with evaluation and management visits).

NSHOS, fielded between June 2017 and August 2018, is a nationally representative set of surveys including a survey of physician practices with at least three adult primary care physicians.^25–29^ NSHOS surveyed a sample of the IQVIA OneKey identified physician practices. Adult primary care physicians included family medicine, geriatrics, internal medicine, and preventive medicine specialties. NSHOS collected information on practices’ structure, ownership, leadership, care delivery capabilities, and participation in delivery reform. NSHOS used a stratified-cluster sampling design that sampled both system-owned and independent physician practices. NSHOS targeted practice managers, physicians, or practice leadership as respondents. NSHOS surveyed 4,976 physician practices; 2,333 practices responded to the survey for a response rate of 46.9%. We excluded 143 practices due to item nonresponse for a total of 2,190 analyzed practices.

### Measures

Beneficiaries’ residence and practices were classified as isolated (<2,500 people; RUCA 10), small town (2,500-9,999 people; RUCA 7-9), micropolitan (10,000-49,999; RUCA 4-6), or metropolitan (>50,000 people; RUCA 1-3) using the Rural Urban Commuting Area (RUCA) codes. Isolated, small town, and micropolitan areas are commonly used to define “rural.”

Using the OneKey data, we described all US practices according to structural, workforce, and geographic characteristics (Table 1). For the NSHOS sample, we further characterized care delivery capabilities using composite scores summarizing the degree to which physician practices report engaging in care delivery processes on responses on payment and delivery reform participation.^29^ The standardized composites average component questions across various domains relating to patient screening, care activities, and practice engagement in reform activities, among other domains.

Using Medicare claims and U.S. Census data, we characterized beneficiaries across rural settings according to their (1) demographics, (2) clinical conditions using hierarchical condition categories, and (3) area-level measures from U.S. Census (Table 3).

We then compared utilization across rural settings including (1) inpatient stays, (2) emergency department visits, (3) outpatient visits, (4) diabetes quality metrics, (5) mammograms, (6) percentage that died, and (6) payments (Table 4).

**Figure 1:**
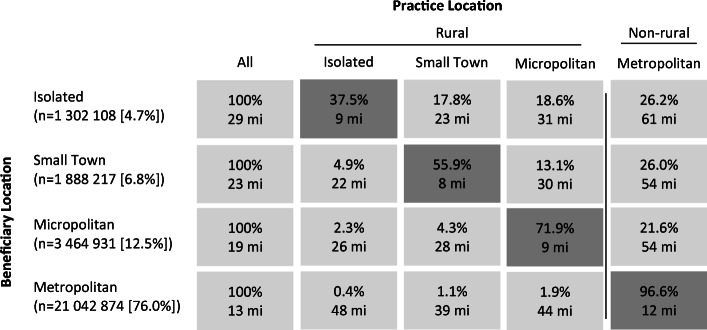
Adjusted practice assignment and distance by beneficiary location, 2017*. Note: Isolated represents RUCA category 10, small town 7-9, micropolitan 4-6, and metropolitan 1-3. *Adjusted for demographic, geographic, and clinical characteristics.

Finally, we considered the proportion of all beneficiaries residing within each rural setting that were cared for by practices located across settings (Figure 1). We computed the straight-line distance in miles between beneficiaries’ ZIP code to the ZIP code of their assigned practice.

#### Statistical Analysis

We first conducted an unadjusted, descriptive analysis of practices to assess organizational differences between isolated, small town, micropolitan, and metropolitan settings (Table 1). To characterize the capabilities of the NSHOS sample of physician practices, we performed unadjusted, descriptive analyses using NSHOS (Table 2). NSHOS analyses used probability weights so that the estimated means and proportions accounted for sampling and non-response and corresponded to the practices included in OneKey. We used *t*-tests and chi-square tests to assess significance.

Next, we performed unadjusted, descriptive analyses to characterize the demographic, clinical, and area-level characteristics of beneficiaries cared for by these practices (Table 3). To compare beneficiary hospital and outpatient utilization across settings, we performed linear and logistic regression modeling to adjust for beneficiary-level demographics, clinical characteristics, and hospital referral regions (adjusted means shown in Table 4; detailed regression output shown in the Appendix).

To estimate the adjusted proportions of beneficiaries residing within each rural setting that were assigned to isolated, small town, micropolitan, and metropolitan practices, we used multinomial logistic models (Figure 1; detailed output shown in the Appendix).

Models were adjusted for the beneficiary demographic (e.g., age, under 65, over 85, sex, race/ethnicity, disabled, dual eligible for Medicaid), clinical (number of chronic conditions, coronary artery disease, congestive heart failure, diabetes, cancer, chronic obstructive pulmonary disease, frail, nursing home resident, death), and area-level characteristics (household income, poverty level, hospital referral regions) shown in Table 3 (detailed measure definitions are shown in the Appendix).

Measures were created using SAS and analyses were conducted using Stata.

## RESULTS

### Practice Characteristics

Our study sample included a total of 116,879 physician practices. Of these, 2,185 (1.9%) were located in isolated settings, 4,502 (3.9%) in small town settings, 11,022 (9.4%) in micropolitan settings, and 99,170 (84.8%) in metropolitan settings (Table 1). Compared to metropolitan practices, isolated practices cared for more attributed Medicare beneficiaries per practice while isolated practices also typically had fewer physicians. Practices in more rural settings had a lower proportion of physicians specializing in internal medicine and a greater proportion in family medicine. Practices in isolated and small town settings were more primary care centric than those in micropolitan and metropolitan settings as evidenced by being less likely to have specialist physicians. Practices in rural settings were more likely to include a nurse practitioner, physician assistance, or clinical nurse specialist (30.5% of isolated practices compared with 20.5% of metropolitan practices). Isolated practices were more commonly owned by a health care system (45.6%) than small town (37.0%), micropolitan (33.4%), and metropolitan (34.2%) practices (Table 1).

**Table 1:** Unadjusted Characteristics of Physician Practices by Rurality of Practice, 2017

	Rural			Non-rural
Characteristics	**Isolated** **(*n*=2185 [1.9%])**	**Small town** **(*n*=4502 [3.9%])**	**Micropolitan (*n*=11,022 [9.4%])**	**Metropolitan (*n*=99,170 [84.8%])**
Median attributed beneficiaries per practice (IQR)	206 (78–412)	189 (29–458)	81 (13–321)	49 (<11*–204)
Physicians, no. (%)				
Solo	1144 (52.4)	2188 (48.6)	5167 (46.9)	39,287 (39.6)
2-5	771 (35.3)	1745 (38.8)	4496 (40.8)	40,811 (41.2)
6–10	56 (2.56)	261 (5.8)	661 (6.0)	11,025 (11.1)
>10	4 (0.2)	14 (0.3)	80 (0.7)	1742 (1.8)
Physician composition (SD)				
Mean % internal medicine	11.6 (28.9)	13.9 (31.4)	14.1 (31.5)	18.5 (35.0)
Mean % family medicine	61.4 (44.7)	45.9 (45.8)	29.6 (42.7)	22.2 (38.4)
Mean % geriatric medicine	0.1 (3.2)	0.1 (3.3)	0.1 (2.9)	0.3 (4.5)
Mean % other primary care^#^	5.1 (20.6)	4.3 (18.4)	2.7 (14.4)	2.7 (14.2)
Mean % specialist	21.8 (38.1)	35.8 (44.6)	53.5 (47.1)	56.3 (47.1)
Primary care physician only, no. (%)	1435 (65.7)	2392 (53.1)	4228 (38.4)	35,406 (35.7)
At least one nurse practitioner, physician assistant, clinical nurse specialist, no. (%)	667 (30.5)	1242 (27.6)	2719 (24.7)	20,358 (20.5)
Ownership, no. (%)				
Independent	1189 (54.4)	2836 (63.0)	7335 (66.6)	65,274 (65.8)
Complex integrated systems^†^	428 (19.6)	756 (16.8)	1557 (14.1)	15,876 (16.0)
Simple integrated systems^‡^	365 (16.7)	513 (11.4)	1 147 (10.4)	5 720 (5.8)
Medical groups^§^	200 (9.2)	394 (8.8)	975 (8.9)	12,229 (12.3)
Census region, no. (%)				
Northeast	354 (16.2)	573 (12.7)	1315 (11.9)	22,604 (22.8)
Midwest	840 (38.4)	1341 (29.8)	2873 (26.1)	18,160 (18.3)
South	675 (30.9)	1995 (44.3)	4913 (44.6)	38,195 (38.5)
West	316 (14.5)	593 (13.2)	1921 (17.4)	20,211 (20.4)

Isolated represents RUCA category 10, small town 7-9, micropolitan 4-6, and metropolitan 1-3

^*^Suppressed beneficiary information

^#^Other primary care: general practice, pediatric, preventive, hospice and palliative, and osteopathic manipulative

^†^Organizations that own at least one physician practice, hospital, and owner subsidiary

^‡^Organizations that own at least one physician practice, at least one hospital, no owner subsidiaries, and not owned by a larger organization

^§^Organizations that own at least two physician practices, no hospitals, and not owned by a larger organization

Of these, 2,189 practices with three or more physicians responded to NSHOS with a similar distribution across rural settings. Regardless of setting, multi-physician practices reported similar capabilities for clinical screenings, managing complex patients, use of evidence-based guidelines, use of electronic health records for decision-making, use of registries, and approaches to physician management. Multi-physician practices in isolated and in micropolitan settings reported significantly less engagement in quality-focused payment programs than practices in metropolitan settings. Differences in quality-focused payment program participation was driven by lower self-reported engagement of isolated practices in accountable care organization (ACO) contracts (Table 2). Significantly fewer isolated (22%) and small town (29%) practices reported an anticipated majority of patients being covered by total cost of care contracts in 5 years, compared to micropolitan (52%) and metropolitan practices (43%; Table 2).

**Table 2:** Physician Practices’ NSHOS Reported Care Delivery Capabilities and Participation in Care Delivery Reform

	Rural			Non-rural
	**Isolated (*n*=42 [1.9%])**	**Small town (*n*=115 [5.3%])**	**Micropolitan (*n*=178 [8.1%])**	**Metropolitan (*n*=1854 [84.7%])**
Mean composite score (SD)^†^				
Clinical screening	83 (25)	81 (23)	82 (25)	81 (24)
Social screening	50 (34)	43 (34)	37 (27)	37 (35)
Complex patients	43 (19)	40 (22)	38 (23)	42 (23)
Depression and anxiety	34 (25)^a^	24 (22)^a^	26 (27)	26 (28)
EHR-based decision making	49 (40)	45 (42)	46 (42)	55 (41)
Use of evidence-based guidelines	65 (32)	58 (39)	57 (43)	60 (40)
Patient engagement	41 (22)	39 (22)	41 (22)^f^	43 (22) ^f^
Physician management	34 (24)	36 (20)	33 (21)	39 (22)
Engagement in quality-focused payment programs	34 (28) ^c^	44 (30)	38 (30) ^f^	46 (29) ^c f^
Use of registries	50 (40)	44 (38)	42 (40)	48 (41)
Current participant in reform, %				
Bundled or episode-based payments	26	30	31	27
Primary care improvement and support programs	66	57	45	53
Pay-for-performance programs	67	71^d^	51^df^	67^f^
Capitated contracts with commercial health plans	24	37	29^f^	48^f^
Medicare ACO, upside-only	20^c^	41	30^f^	41^cf^
Medicare ACO, risk bearing	6^abc^	21^a^	21^b^	28^c^
Medicaid ACO	23	38	35	34
Commercial ACO	16^abc^	37^a^	36^b^	45^c^
In 5 years, majority of patients anticipated to be covered by total cost of care accountability contracts, no. (%)	22^bc^	29^de^	52^bd^	43^ce^

Isolated represents RUCA category 10, small town 7-9, micropolitan 4-6, and metropolitan 1-3

^*^Percentages are adjusted with survey weights

^†^Composite scores range from 0 to 100 and summarize the degree to which physician practices report engaging in various care delivery processes in each of these domains

Significance at *p*<0.05 level: a = isolated vs. small town; b = isolated vs. micro; c = isolated vs. metro; d = small town vs. micro; e = small town vs. metro; f = micro vs. metro

### Beneficiary Characteristics

Comparing between practice settings, beneficiaries cared for by more rural practices were more often White, more likely to be disabled, dually eligible for Medicare and Medicaid, and from areas with greater poverty. Beneficiaries cared for by metropolitan practices were more likely to have cancer, end-stage renal disease, frailty, and nursing facility care use (Table 3).

**Table 3:** Unadjusted Characteristics of Medicare Beneficiaries by Rurality of Assigned Practice, 2017

	Rural			Non-rural
Characteristics	**Isolated (*n*=743,196 [2.7%])**	**Small town (*n*=1,662,480 [6.0%])**	**Micropolitan (*n*=3,384,913 [12.2%])**	**Metropolitan (*n*=21,926,378 [79.1%])**
Race, no. (%)				
White	676 284 (91.0)^abc^	1 492 898 (89.8)^ade^	3 031 561 (89.6)^bdf^	18 158 880 (82.8)^cef^
Black	27 649 (3.7)^abc^	104 487 (6.3)^ade^	206 896 (6.1)^bdf^	2 076 923 (9.5)^cef^
Hispanic	3 664 (0.5)^abc^	11 667 (0.7)^ade^	35 694 (1.1)^bdf^	433 805 (2.0)^cef^
Other	35 599 (4.8)^abc^	53 428 (3.2)^ade^	110 762 (3.3)^bdf^	1 256 770 (5.7)^cef^
Age, no. (%)				
<65	125 986 (17.0)^abc^	292 302 (17.6)^ade^	574 740 (17.0)^bdf^	3 170 749 (14.5)^cef^
65–69	180 328 (24.3)^abc^	400 288 (24.1)^ade^	847 499 (25.0)^bdf^	5 681 353 (25.9)^cef^
70–74	149 141 (20.1)^abc^	330 576 (19.9)^ade^	690 772 (20.4)^bdf^	4 646 083 (21.2)^cef^
75–79	114 883 (15.5)^abc^	256 864 (15.5)^ade^	522 817 (15.5)^bdf^	3 393 573 (15.5)^cef^
80–84	82 659 (11.1)^abc^	184 996 (11.1)^ade^	368 475 (10.9)^bdf^	2 394 567 (10.9)^cef^
>85	90 199 (12.1)^abc^	197 454 (11.9)^ade^	380 610 (11.2)^bdf^	2 640 053 (12.0)^cef^
Female, no. (%)	412 404 (55.5)^abc^	935 605 (56.3)^ae^	1 903 452 (56.2)^bf^	12 421 259 (56.7)^cef^
Disabled, no. (%)	203 111 (27.3)^abc^	469 196 (28.2)^ade^	907 686 (26.8)^bdf^	4 871 285 (22.2)^cef^
Dual eligibility, no. (%)^‡^	135 020 (18.2)^abc^	294 372 (17.7)^ade^	545 350 (16.1)^bdf^	3 320 780 (15.2)^cef^
Area-level characteristics				
High poverty, no. (%)*	182 578 (24.6)^abc^	524 872 (31.6)^ade^	946 366 (28.0)^bdf^	3 766 341 (17.2)^cef^
Mean household income (SD)^$†^	43 373 (12 492)^abc^	42 244 (12 403)^ade^	44 877 (14 014)^bdf^	60 899 (26 933)^cef^
Hierarchical condition categories, no. (%)				
0	309 929 (41.7)^abc^	662 808 (39.9)^ade^	1 322 182 (39.1)^bdf^	8 381 514 (38.2)^cef^
1–2	295 130 (39.7)^abc^	674 426 (40.6)^ade^	1 380 155 (40.8)^bdf^	8 874 590 (40.5)^cef^
3–5	101 960 (13.7)^abc^	238 999 (14.4)^ade^	495 707 (14.6)^bdf^	3 300 920 (15.1)^cef^
>6	36 177 (4.9)^abc^	86 247 (5.2)^ade^	186 869 (5.5)^bdf^	1 369 354 (6.3)^cef^
Clinical conditions, no. (%)				
Congestive heart failure	73 797 (9.9)^abc^	171 832 (10.3)^ade^	344 879 (10.2)^bdf^	2 246 046 (10.2)^cef^
Coronary artery disease	33 861 (4.6)^abc^	81 045 (4.9)^ade^	171 305 (5.1)^bdf^	1 091 265 (5.0)^cef^
Diabetes	184 525 (24.8)^abc^	432 568 (26.0)^ade^	870 855 (25.7)^bdf^	5 364 110 (24.5)^cef^
Cancer	53 953 (7.3)^abc^	125 539 (7.6)^ade^	280 250 (8.3)^bdf^	2 223 660 (10.1)^cef^
Chronic obstructive pulmonary disease	91 633 (12.3)^abc^	210 896 (12.7)^ade^	412 711 (12.2)^bdf^	2 186 411 (10.0)^cef^
End-stage renal disease	5 298 (0.7)^abc^	14 086 (0.9)^ade^	32 806 (1.0)^bdf^	290 126 (1.3)^cef^
Any nursing facility care, no. (%)	26 577 (3.6)^abc^	65 139 (3.9)^ade^	139 517 (4.1)^bdf^	1 067 458 (4.9)^cef^
Frail, no. (%)^§^	24 862 (3.4)^abc^	60 584 (3.6)^ade^	126 315 (3.7)^bdf^	1 126 651 (5.1)^cef^

Isolated represents RUCA category 10, small town 7-9, micropolitan 4-6, and metropolitan 1-3

*>20% of the population falls under the federal poverty level at census tract level

^†^Median household income at zip code level

^‡^Dual eligible for Medicare and Medicaid

^§^Beneficiaries who are >64 years and meet at least two of the following conditions or services: gait abnormality, cachexia, debility, durable medical equipment use, difficulty walking, failure to thrive, history of falling, fatigue, malnutrition, muscle wasting, muscle weakness, nursing services, senility, ulcer

Significance of difference at *p*<0.05 level: a = isolated vs. small town; b = isolated vs. micro; c = isolated vs. metro; d = small town vs. micro; e = small town vs. metro; f = micro vs. metro

### Utilization and Quality Measures

There were few substantive, clinically meaningful differences after risk adjustment across practice settings for most utilization measures including inpatient stays, emergency department visits, emergency department visits by necessity, and patterns of outpatient visits. Readmission measures were a noteworthy exception with beneficiaries in isolated and small town practices having higher 30-day readmission rates than those cared for in practices in micropolitan and metropolitan areas including for all-cause medical discharges, surgical discharges, heart failure discharges, acute myocardial infarction, and pneumonia discharges.

Fewer beneficiaries with diabetes cared for in rural practices had an annual eye exam (64.9% in isolated vs. 69.0% in metropolitan), yet similar proportions of beneficiaries with diabetes had a blood lipids and hemoglobin A1c tests. Fewer beneficiaries in isolated rural practices received a recommended mammogram (59.8%) compared with other settings (64.6% small town, 64.6% micropolitan, 65.3% in metropolitan).

Payments for beneficiaries cared for in isolated practices cost, on average (per beneficiary), $725 more than beneficiaries cared for in metropolitan practices after risk adjustment, with much of the difference due to higher payments for acute care in isolated settings (Table 4).

**Table 4:** Adjusted Beneficiary Utilization by Rurality of Assigned Practice, 2017*

	Rural			Non-rural
	**Isolated (*n*=743,196 [2.7%])**	**Small town (*n*=1 662,480 [6.0%])**	**Micropolitan (*n*=3,384,913 [12.2%])**	**Metropolitan (*n*=21,926,378 [79.1%])**
Inpatient stays				
Mean number stays per beneficiary	0.3 (0.3–0.3)^abc^	0.3 (0.3–0.3)^ade^	0.3 (0.3–0.3)^bdf^	0.3 (0.3–0.3)^cef^
% of beneficiaries with multiple stays	7.1 (7.0–7.1)^abc^	6.9 (6.9–7.0)^ade^	6.6 (6.5–6.6)^bdf^	6.4 (6.4–6.4)^cef^
% with a potentially avoidable stay				
Acute stays	2.2 (2.2–2.2)^abc^	2.1 (2.1–2.2)^ade^	1.8 (1.8–1.8)^bdf^	1.6 (1.6–1.7)^cef^
Chronic stays	2.8 (2.8–2.8)^abc^	2.7 (2.7–2.8)^ade^	2.7 (2.7–2.7)^bd^	2.7 (2.7–2.7)^ce^
Readmissions				
30-day all-cause for medical discharges	20.1 (20.5–21.0)^abc^	20.3 (20.1–20.5)^ade^	18.1 (18–18.2)^bd^	18.0 (18.0–18.0)^ce^
30-day all-cause for surgical discharges	13.2 (12.9–13.5)^bc^	13.5 (13.2–13.7)^de^	12.6 (12.4–12.7)^bdf^	12.3 (12.2–12.3)^cef^
30-day all-cause for acute myocardial infarction discharges	16.9 (15.8–18)^bc^	16.7 (16–17.4)^de^	15.2 (14.7–15.7)^bd^	15.6 (15.4–15.8)^ce^
30-day all-cause for congestive heart failure discharges	25.7 (24.7–26.6)^bc^	25.2 (24.6–25.8)^de^	23.7 (23.2–24.1)^bd^	23.1 (23.1–23.4)^ce^
30-day all-cause for pneumonia discharges	21.4 (20.6–22.1)^abc^	19.9 (19.4–20.3)^ade^	17.3 (16.9–17.6)^bd^	16.9 (16.8–17.1)^ce^
Emergency department visits				
Mean number of visits per beneficiaries, discharged	0.5 (0.5–0.5)^abc^	0.6 (0.6–0.6)^ade^	0.5 (0.5–0.5)^bdf^	0.5 (0.5–0.5)^cef^
% with multiple visits discharged	15.9 (15.9–16.0)^abc^	16.7 (16.6–16.7)^ade^	16.3 (16.3–16.4)^bdf^	15.0 (15.0–15.0)^cef^
Mean % visits that were†				
Necessary, but preventable	10.8 (10.7–10.9)^bc^	10.7 (10.7–10.8)^de^	10.2 (10.1–10.3)^bdf^	10.0 (9.9–10.0)^cef^
Unnecessary, but emergent	33.5 (33.3–33.7)^ac^	33.9 (33.7–34.0)^ade^	33.4 (33.3–33.5)^df^	32.5 (32.5–32.6)^cef^
Unnecessary and nonemergent	25.0 (24.8–25.2)^c^	25.0 (24.9–25.1)^e^	25.0 (24.9–25.1)^f^	25.2 (25.2–25.2)^cef^
% died	4.0 (4.0–4.1)^bc^	4.0 (4.0–4.0)^de^	3.9 (3.9–4.0)^bdf^	3.9 (3.9–3.9)^cef^
Quality metrics				
Diabetics who had a blood lipid test	76.3 (76.1–76.4)^abc^	76.5 (76.3–76.6)^ade^	76.9 (76.8–76.9)^bd^	76.8 (76.8–76.8)^ce^
Diabetics who had an eye exam	64.9 (64.7–65.1)^abc^	66.3 (66.2–66.4)^ade^	67.8 (67.3–67.9)^bdf^	69.0 (69.0–69.1)^cef^
Diabetics who had hemoglobin A1c test	86.0 (85.9–86.2)^a^	86.3 (86.2–86.4)^ade^	86.1 (86.1–86.2)^df^	86.0 (85.9–86.0)^ef^
Mammogram, aged 50–74	59.8 (59.6–60.1)^abc^	64.6 (61.8–62.1)^ade^	64.6 (64.5–64.7)^bdf^	65.3 (65.3–65.4)^cef^
Access Measures				
Primary care clinician visit within 14 days of stay	67.1 (66.7–67.5)^abc^	65.3 (65.1–65.6)^ade^	62.1 (61.9–62.3)^bdf^	58.1 (58.1–58.2)^cef^
Follow-up within 30 days of mental health stay	70.2 (68.8–71.6)^c^	71.1 (70.2–72)^e^	70.5 (69.9–71.1)^f^	67.9 (68–68.2)^cef^
Follow-up within 7 days of mental health stay	39.7 (38.2–41.2)	40.3 (39.4–41.3)^de^	39.1 (38.4–39.8)^d^	38.8 (38.5–39)^e^
Mean number of outpatient visits				
Overall	11.0 (11.0–11.0)^abc^	10.8 (10.8–10.8)^ade^	11.1 (11.1–11.1)^bdf^	11.3 (11.3–11.3)^cef^
Family medicine	2.6 (2.6–2.6)^abc^	2.5 (2.5–2.5)^ade^	2.1 (2.1–2.1)^bdf^	1.8 (1.8–1.8)^cef^
Internist	1.2 (1.2–1.2)^abc^	1.4 (1.4–1.4)^ade^	1.8 (1.8–1.8)^bdf^	2.0 (2.0–2.0)^cef^
Geriatrician	0.02 (0.02–0.02)^c^	0.02 (0.02–0.02)^e^	0.02 (0.02–0.02)^f^	0.06 (0.06–0.06)^cef^
Other primary care	0.2 (0.2–0.2)^abc^	0.2 (0.2–0.2)^ade^	0.1 (0.1–0.1)^bdf^	0.1 (0.1–0.1)^cef^
Specialist	4.3 (4.3–4.3)^abc^	4.3 (4.34.3)^ade^	4.6 (4.6–4.6)^bdf^	5.0 (5.0–5.0)^cef^
Nurse practitioner, physician assistant, clinical nurse specialist	1.8 (1.8–1.8)^abc^	1.5 (1.5–1.5)^ade^	1.8 (1.8–1.8)^bdf^	1.7 (1.7–1.7)^cef^
Mean number of providers encountered				
Primary care physician	1.0 (1.0–1.0)^abc^	1.1 (1.1–1.1)^ade^	1.1 (1.1–1.1)^bdf^	1.2 (1.2–1.2)^cef^
Specialist	2.0 (2.0–2.0)^abc^	2.0 (2.0–2.0)^ade^	2.1 (2.1–2.1)^bdf^	2.3 (2.3–2.3)^cef^
Nurse practitioner, physician assistant, clinical nurse specialist	0.7 (0.7–0.7)^abc^	0.6 (0.6–0.6)^ade^	0.8 (0.8–0.8)^bdf^	0.8 (0.8–0.8)^cef^
Payments, $				
Total	11,501 (11,461–11,541)^abc^	11,248 (11,221–11,275)^ade^	10,784 (10,764–10,804)^bd^	10,776 (10,769–10,784)^ce^
Acute care	3862 (3839–3886)^abc^	3758 (3742–3774)^ade^	3541 (3530–3552)^bdf^	3407 (3403–3412)^cef^
Procedures	1568 (1561–1575)^c^	1571 (1567–1576)^e^	1571 (1568–1574)^f^	1583 (1582–1584)^cef^
Evaluation and management	1206 (1199–1212)^abc^	1233 (1229–1238)^ade^	1267 (1264–1270)^bdf^	1336 (1335–1338)^cef^
Other	4211 (4187–4234)^b^	4183 (4167–4199)^d^	4108 (4096–4119)^bdf^	4197 (4192–4201)^f^

Isolated represents RUCA category 10, small town 7-9, micropolitan 4-6, and metropolitan 1-3

^*^Adjusted for demographic, geographic, and clinical characteristics

^†^NYU ED visit algorithm: necessary, but preventable = probability >0.5 visit requires ED and is potentially preventable through primary care; unnecessary, but emergent = probability >0.5 visit is emergent but does not require ED; unnecessary and nonemergent = probability >0.5 visit is nonemergent

Significance of difference at *p*<0.05 level: a = isolated vs. small town; b = isolated vs. micro; c = isolated vs. metro; d = small town vs. micro; e = small town vs. metro; f = micro vs. metro

### Access to Outpatient Care

Patterns of adjusted outpatient visits with primary care clinicians were strikingly similar between rural and non-rural settings. The number of visits per year with primary care clinicians was similar regardless of practice setting. While the overall visit rate was similar across settings, the share of visits with family medicine versus internists varied by rurality. Beneficiaries cared for by metropolitan practices had slightly more visits to specialist physicians than those cared for by isolated practices (5.0 vs. 4.3). Beneficiaries cared for in rural practices were more likely to have a follow-up outpatient visit after an inpatient stay than those cared for in non-rural practices.

Beneficiaries living in more rural areas were less likely to receive primary care in the setting where they resided: 37.5% of beneficiaries residing in isolated settings are cared for by practices within the same setting, compared to 55.9% of small town beneficiaries, 71.9% of micropolitan beneficiaries, and 96.6% of metropolitan beneficiaries (Figure 1). Beneficiaries traveled farther as they were cared for by practices in more populated settings than which they resided. Over a quarter (26.2%) of isolated beneficiaries were cared for by practices greater than 60 miles from their residence.

## DISCUSSION

We found that rural practices have fewer physicians, are more primary care and family medicine oriented, have more nurse practitioners, and are more likely to be owned by a health care system. Despite concerns about rural practices’ capabilities, in part due to workforce shortages,^30^ we found that rural and non-rural multi-physician practices report similar care delivery capabilities. Patients in rural practices have similar utilization patterns for most measures with few noteworthy differences: rural practices have both higher adjusted readmission rates and greater follow-up visit rates after a hospital stay. Rural patients have similar access to care in terms of number of outpatient visits, yet they may experience greater challenges reaching care.

We found that the structure and configuration of physician practices varied among rural settings with practices in micropolitan areas more similar to those in metropolitan areas in terms of number of physicians, composition of physicians, and ownership. With fewer specialist clinicians available, primary care physicians in isolated areas face different challenges than physicians in micropolitan areas and may therefore benefit from different solutions. For example, isolated primary care clinicians may benefit more from virtual access to specialists (through, for example, the ECHO model^31^) while micropolitan primary care clinicians may need more robust referral networks in metropolitan areas.

We found that rural patients tend to travel farther for their care with a quarter of isolated patients traveling more than 60 miles. The increased travel burden among rural patients likely drives perceptions of inaccessibility. A recent survey found that one in four rural residents report not being able to access health care when they needed it—22% of those said it was too far or difficult to get care.^22^ Increased access to telehealth could relieve some of the travel burden that rural patients experience. Despite challenges accessing care, the number of outpatient encounters does not vary much across rural settings, indicating that patients may be getting the care they need, despite the increased burden.

Our findings suggest that, despite challenges, Medicare patients may be able to adequately access primary care services in rural settings. This finding aligns with and advances prior research showing adults under 65 report comparable access to office-based visits between rural and urban settings regardless of insurance status.^32^ Our study advances this prior research by disaggregating rurality into isolated, small town, and micropolitan settings and by examining access at the practice level. Yet, our finding that practices in rural settings have two to four times as many attributed Medicare beneficiaries (despite having, on average, fewer clinicians) than practices in urban settings lends support to concerns that the supply of primary care clinicians is inadequate in rural settings. The growth of nurse practitioners and other non-physician clinicians in rural settings has likely helped ensure patients can access care.^33,34^ Considering the rural health care workforce is aging and fewer new physicians join rural practices, policymakers should remain focused on ensuring there is a sufficient primary care workforce pipeline in rural settings.^35^ There has been concern that lack of access to primary care in rural settings could result in delays in seeking care which could exacerbate chronic conditions and/or greater utilization at more costly, hospital-based settings.^9^

Further, our findings on care for patients with diabetes and mammograms suggest that rural practices may find it challenging to coordinate with ancillary services that happen outside of clinic walls (e.g., imaging, ophthalmologists) because access to other facilities is limited in rural areas. This lack of specialist and facility-based services has likely made rural primary care particularly adept at delivering more comprehensive care (i.e., through more office-based procedures and a broader set of conditions managed) within their clinic walls.^17,18,21,36^ More comprehensive primary care—where primary care meets the majority of a patient’s physical and common mental health needs^17,37,38^—likely improves patients’ outcomes overall.^16,38^ Primary care could use further support developing adequate, robust networks of specialists; policymakers and payers could incentivize virtual networks such as visiting specialist or mobile clinics^39^ for services that primary care cannot deliver.

In terms of rural primary care, much of the focus by policymakers has been on access to care and care delivery capabilities. Policymakers have worried that small, rural practices will be under-resourced with fewer staff and technology solutions available to coordinate care which may impact their ability to participate in value-based care.^19,40^ We do not find striking urban-rural disparities in the care delivery capabilities of multi-physician practices. However, we found self-reported participation in payment reform, especially among isolated rural practices in ACO contracts, was lower for rural multi-physician practices. Care delivery capabilities in rural multi-physician practices may be driven, in part, by system ownership. System-ownership typically accelerates practices’ participation in risk-based contracting, but ownership may not be a sufficient motivator for practices in isolated settings given other barriers.^29,41^ Rural practices may be particularly at risk for acquisition by health systems, especially hospital-based systems,^42^ as a way to improve financial viability for both the practice and the hospital. The risk for acquisition is likely amplified by the ongoing pandemic with rural practices particularly vulnerable in the face of reduced visit rates and likely with fewer resources to weather-sustained losses.

This study has key limitations. First, we assigned patients to practices based on Medicare billing using outpatient evaluation and management visits as a measure of primary care. Second, this study may not be representative to patients covered by other payers or, especially, those that are uninsured. Medicare policy can have an outsized impact on rural areas because rural populations are aging faster than their urban counterparts.^43^ Third, our findings on care delivery capabilities were limited to practices with three or more physicians while nearly half of rural primary care practices have a single physician.

Despite challenges in rural primary care related to clinician supply and complex patient populations, our study finds relatively few utilization differences between Medicare patients cared for by rural and non-rural practices. Yet, rural primary care clinicians may be stressed given they deliver care for larger Medicare patient panels, with a dwindling workforce, compared to their urban counterparts. Further, for Medicare patients, there is often considerable travel burden associated with accessing primary care. Policymakers can ease challenges faced by clinicians and patients by ensuring there is a sufficient ongoing rural primary care workforce, by supporting coordination with specialist networks, and by considering sustainable payment strategies to support team-based comprehensive care that can survive without acquisition by larger health systems.

## Supplementary Information

ESM 1 (DOCX 271 kb)

## Declarations

### Disclaimer

The statements, findings, conclusions, views, and opinions contained and expressed herein are not necessarily those of IQVIA Inc. or AHRQ or any of its affiliated or subsidiary entities.
