# Supplementary material for: Configuration and Delivery of Primary Care in Rural and Urban Settings
Source: J Gen Intern Med. 2022 Mar 9;37(12):3045–53. doi: 10.1007/s11606-022-07472-x (PMC9485295; doi:10.1007/s11606-022-07472-x)
Supplement: Supplementary file 1 — (DOCX 271 kb) [file 11606_2022_7472_MOESM1_ESM.docx]

**Supplemental Material**

**Qualifying evaluation and management codes for attribution**

| **HCPCS code** | **Description** |
| --- | --- |
| 99201–99205 | New patient, office, or other outpatient visit |
| 99211–99215 | Established patient, office, or other outpatient visit |
| 99304–99306 | New patient, nursing facility care |
| 99307–99310 | Established patient, nursing facility care |
| 99315–99316 | Established patient, discharge day management service |
| 99318 | Established patient, other nursing facility service |
| 99324–99328 | New patient, domiciliary or rest home visit |
| 99334–99337 | Established patient, domiciliary or rest home visit |
| 99339–99340 | Established patient, physician supervision of patient (patient not present) in home, domiciliary, or rest home |
| 99341–99345 | New patient, home visit |
| 99347–99350 | Established patient, home visit |
| G0402 | Initial Medicare visit |
| G0438-G0439 | Annual wellness visit, initial or subsequent |
| G0463 | Hospital outpatient clinic visit (Electing Teaching Amendment hospitals only) |

| **Measure Name** | **Description** |
| --- | --- |
| Primary care physicians | Family practice, general practitioner, internal medicine, geriatrician. Defined using MD-PPAS |
| Nurse practitioner | Defined using MD-PPAS |
| Specialist physicians | Any physician (MD/DO) who is not a primary care physician |
| **Demographics** |  |
| Age | Integer age at beginning of year |
| Median household income | Median household income for beneficiary ZIP Code using US Census Bureau's American Community Survey |
| Residents under poverty level | Percent of population under the federal poverty level by census track. Defined by linking beneficiary ZIP Code with US Census Bureau's American Community Survey |
| Race | Defined from Master Beneficiary Summary Base |
| Disabled | Indicates beneficiary's original reason for Medicare entitlement due to disability |
| Dual eligible for Medicaid | Indicate dual eligibility for Medicare/Medicaid during year |
| Rurality | Defined using rural urban commuting codes (RUCA) as isolated rural, small town, micropolitan, or metropolitan. isolated rural, small town, and micropolitan are typically considered "rural" |
| Died | Died in the calendar year |
| **Clinical Characteristics** |  |
| Hierarchical condition categories | Defined using CMS-HCC grouper v22 |
| Coronary artery disease | Derived from HCCs 86, 87, or 88 |
| Congestive heart failure | Derived from HCCs 85 |
| Diabetes | Derived from HCCs 17, 18, 19, or 122 |
| Cancer | Derived from HCCs 8, 9, 10, 11, 12 |
| Chronic obstructive pulmonary disease | Derived from HCC 111 |
| Frail | Beneficiaries who are >64 years and meet at least two of the following conditions or services: gait abnormality, cachexia, debility, durable medical equipment use, difficulty walking, failure to thrive, history of falling, fatigue, malnutrition, muscle wasting, muscle weakness, nursing services, senility, ulcer |
| Nursing home care | If the beneficiary used nursing facility care |
| End-stage renal disease | Indicator derived from the denominator file |
| **Inpatient and Outpatient Utilization** |  |
| Inpatient stays | Number of admissions/inpatient stays to acute care/critical access hospitals per beneficiary |
| Discharges for potentially avoidable stays conditions | Defined using Agency for Healthcare Quality and Research's Prevention Quality Indicators, acute and chronic composites |
| Readmissions | Number of unplanned hospital readmissions within 30 days of index stay for given condition. Follows Yale-CMS methods. |
| Emergency department visits discharged to home | Number of emergency department visits from outpatient claims and/or MEDPAR claims per beneficiary |
| Emergency department visits that were necessary, but preventable; unnecessary, but emergent, or unnecessary and nonemergent | We used the New York University’s ED profiling algorithm to classify visits as: (1) necessary, but preventable; (2) unnecessary, but emergent; or (3) unnecessary and nonemergent. Visits were assigned when the initial complaint had a greater than 50% probability of falling into one of the three classifications. |
| Diabetics who had a blood lipids test | Diabetic beneficiaries age 18-99 who had blood lipids test.  Blood lipids testing: CPT codes 80061, 83700, 83701, 83704, 83715, 83716, 83721; CPT II codes 3048F, 3049F, 3050F. |
| Diabetics who had an eye exam | Diabetic beneficiaries age 18-99 who had eye exam. |
| Diabetics who had hemoglobin A1c test | Diabetic patient age 18-99 who had hemoglobin A1c test. |
| Mammogram, aged 50-74 | Mammogram indicated - female beneficiary age 50-74 |
| Primary care clinician visit within 14-days of stay | Beneficiary has at least one ambulatory outpatient visit within 14 days of discharge. |
| Follow-up after a mental health stay | Proportion of beneficiaries with a qualifying mental health stay who have a 14 or 30-day visit for mental health. |
| Outpatient visits, by clinician type | Number of outpatient visits (qualifying evaluation and management visits as part of attribution) by type of clinician. |
| Number of clinicians encountered, by clinician type | Number of clinicians encountered in the year. |
| **Payment Categories** |  |
| Total payments | Defined using Berenson-Eggers Type of Services (BETOS) codes. Sum of all codes |
| Acute care hospital payments | Defined using Berenson-Eggers Type of Services (BETOS) codes |
| Other inpatient payments | Defined using Berenson-Eggers Type of Services (BETOS) codes. Combined long term care and skilled nursing facility payments |
| Other payments | Defined using Berenson-Eggers Type of Services (BETOS) codes. Durable medical equipment, imaging, evaluation and management visits, procedures, tests, outpatient facilities, home health agency, hospice, and other/exceptions/unclassified payments |
| **NSHOS Composite Measures** | 10 composite measures measure care delivery processes and are detailed in a recent article by Fisher et al. |

| **Inpatient stays** |  |  |  |  |  |  |
| --- | --- | --- | --- | --- | --- | --- |
| N | 27,710,872 |  |  |  |  |  |
| R^2^ | 0.502 |  |  |  |  |  |
|  | **Coefficient** | **SE** | **t** | **P-value** | **LL CI** | **UL CI** |
| Small Town vs. Isolated | -0.007 | 0.001 | -8.150 | 0.000 | -0.008 | -0.005 |
| Micropolitan vs. Isolated | -0.022 | 0.001 | -28.890 | 0.000 | -0.024 | -0.021 |
| Metropolitan vs. Isolated | -0.031 | 0.001 | -42.930 | 0.000 | -0.032 | -0.030 |
| Frail | 0.206 | 0.001 | 368.450 | 0.000 | 0.205 | 0.207 |
| Mean age | -0.004 | 0.000 | -233.370 | 0.000 | -0.004 | -0.004 |
| Under 65 | -0.016 | 0.001 | -25.920 | 0.000 | -0.017 | -0.015 |
| Over 85 | -0.018 | 0.000 | -36.560 | 0.000 | -0.019 | -0.017 |
| Female | 0.024 | 0.000 | 104.320 | 0.000 | 0.023 | 0.024 |
| White vs. Unknown | -0.015 | 0.001 | -15.920 | 0.000 | -0.016 | -0.013 |
| Black vs. Unknown | 0.006 | 0.001 | 5.600 | 0.000 | 0.004 | 0.008 |
| Other vs. Unknown | 0.002 | 0.001 | 1.420 | 0.155 | -0.001 | 0.004 |
| Asian vs. Unknown | 0.020 | 0.001 | 15.500 | 0.000 | 0.017 | 0.022 |
| Hispanic vs. Unknown | 0.041 | 0.001 | 32.110 | 0.000 | 0.038 | 0.043 |
| North American Native vs. Unknown | 0.045 | 0.002 | 25.310 | 0.000 | 0.041 | 0.048 |
| Disabled (original reason for Medicare eligibility) | -0.070 | 0.000 | -164.880 | 0.000 | -0.071 | -0.069 |
| Dual eligible for Medicaid | -0.072 | 0.000 | -210.530 | 0.000 | -0.072 | -0.071 |
| Nursing home | 0.249 | 0.000 | 531.630 | 0.000 | 0.248 | 0.250 |
| Died in the year | 0.138 | 0.001 | 221.380 | 0.000 | 0.137 | 0.139 |
| Median household income (area) | 0.000 | 0.000 | -2.030 | 0.042 | 0.000 | 0.000 |
| Residents under poverty level | 0.000 | 0.000 | 24.210 | 0.000 | 0.000 | 0.000 |
| Number of hierarchical condition categories | 0.278 | 0.000 | 3,021.230 | 0.000 | 0.278 | 0.278 |
| Coronary artery disease | 0.102 | 0.001 | 184.110 | 0.000 | 0.101 | 0.103 |
| Congestive heart failure | -0.022 | 0.000 | -47.300 | 0.000 | -0.022 | -0.021 |
| Diabetes | -0.259 | 0.000 | -884.430 | 0.000 | -0.259 | -0.258 |
| Cancer | -0.139 | 0.000 | -346.210 | 0.000 | -0.140 | -0.139 |
| Chronic obstructive pulmonary disease | -0.073 | 0.000 | -177.170 | 0.000 | -0.073 | -0.072 |
| End stage renal disease | 0.030 | 0.001 | 28.680 | 0.000 | 0.028 | 0.033 |
| Midwest vs. Northeast | 0.018 | 0.003 | 6.800 | 0.000 | 0.013 | 0.024 |
| South vs. Northeast | 0.013 | 0.003 | 4.770 | 0.000 | 0.008 | 0.018 |
| West vs. Northeast | -0.002 | 0.004 | -0.530 | 0.593 | -0.009 | 0.005 |
| Hospital referral region (suppressed) |  |  |  |  |  |  |
| Constant | 0.281 | 0.003 | 81.130 | 0.000 | 0.275 | 0.288 |
| **Practice Location** | **Margin** | **SE** | **t** | **P-value** | **LL CI** | **UL CI** |
| Isolated Rural | 0.335 | 0.001 | 476.100 | 0.000 | 0.334 | 0.337 |
| Small Town | 0.329 | 0.000 | 686.190 | 0.000 | 0.328 | 0.330 |
| Micropolitan | 0.313 | 0.000 | 910.850 | 0.000 | 0.313 | 0.314 |
| Metropolitan | 0.304 | 0.000 | 2348.980 | 0.000 | 0.304 | 0.305 |
| **Practice Location** | **Contrast** | **SE** | **t** | **P-value** |  |  |
| Small town vs. Isolated Rural | -0.007 | 0.001 | -8.150 | 0.000 |  |  |
| Micropolitan vs. Isolated Rural | -0.022 | 0.001 | -28.890 | 0.000 |  |  |
| Metropolitan vs. Isolated rural | -0.031 | 0.001 | -42.930 | 0.000 |  |  |
| Micropolitan vs. Small Town | -0.015 | 0.001 | -27.130 | 0.000 |  |  |
| Metropolitan vs. Small Town | -0.024 | 0.001 | -48.110 | 0.000 |  |  |
| Metropolitan vs. Micropolitan | -0.009 | 0.000 | -23.540 | 0.000 |  |  |

| **Multiple inpatient stays** |  |  |  |  |  |  |
| --- | --- | --- | --- | --- | --- | --- |
| N | 27,710,872 |  |  |  |  |  |
| R^2^ | 0.471 |  |  |  |  |  |
|  | **OR** | **SE** | **Z Score** | **P-value** | **LL CI** | **UL CI** |
| Small Town vs. Isolated | 0.965 | 0.007 | -4.650 | 0.000 | 0.951 | 0.980 |
| Micropolitan vs. Isolated | 0.868 | 0.006 | -20.060 | 0.000 | 0.856 | 0.880 |
| Metropolitan vs. Isolated | 0.820 | 0.005 | -30.000 | 0.000 | 0.809 | 0.830 |
| Frail | 1.926 | 0.006 | 203.740 | 0.000 | 1.914 | 1.938 |
| Mean age | 0.990 | 0.000 | -61.090 | 0.000 | 0.989 | 0.990 |
| Under 65 | 1.255 | 0.006 | 44.230 | 0.000 | 1.242 | 1.268 |
| Over 85 | 1.151 | 0.005 | 36.030 | 0.000 | 1.142 | 1.160 |
| Female | 1.146 | 0.002 | 63.610 | 0.000 | 1.142 | 1.151 |
| White vs. Unknown | 1.006 | 0.011 | 0.570 | 0.570 | 0.985 | 1.028 |
| Black vs. Unknown | 1.061 | 0.012 | 5.080 | 0.000 | 1.037 | 1.085 |
| Other vs. Unknown | 0.992 | 0.015 | -0.510 | 0.609 | 0.964 | 1.022 |
| Asian vs. Unknown | 1.049 | 0.015 | 3.310 | 0.001 | 1.020 | 1.080 |
| Hispanic vs. Unknown | 1.302 | 0.018 | 19.600 | 0.000 | 1.268 | 1.337 |
| North American Native vs. Unknown | 1.400 | 0.023 | 20.200 | 0.000 | 1.355 | 1.446 |
| Disabled (original reason for Medicare eligibility) | 0.890 | 0.003 | -33.790 | 0.000 | 0.884 | 0.896 |
| Dual eligible for Medicaid | 0.786 | 0.002 | -86.910 | 0.000 | 0.781 | 0.790 |
| Nursing home | 2.019 | 0.005 | 259.810 | 0.000 | 2.009 | 2.030 |
| Died in the year | 1.171 | 0.004 | 47.160 | 0.000 | 1.163 | 1.178 |
| Median household income (area) | 1.000 | 0.000 | -13.800 | 0.000 | 1.000 | 1.000 |
| Residents under poverty level | 1.002 | 0.000 | 10.410 | 0.000 | 1.001 | 1.002 |
| Number of hierarchical condition categories | 2.080 | 0.001 | 1206.370 | 0.000 | 2.078 | 2.083 |
| Coronary artery disease | 1.328 | 0.004 | 92.520 | 0.000 | 1.320 | 1.336 |
| Congestive heart failure | 1.091 | 0.003 | 33.510 | 0.000 | 1.085 | 1.096 |
| Diabetes | 0.602 | 0.001 | -214.860 | 0.000 | 0.599 | 0.605 |
| Cancer | 1.100 | 0.003 | 33.860 | 0.000 | 1.094 | 1.106 |
| Chronic obstructive pulmonary disease | 1.013 | 0.003 | 4.960 | 0.000 | 1.008 | 1.018 |
| End stage renal disease | 0.716 | 0.004 | -58.810 | 0.000 | 0.708 | 0.724 |
| Midwest vs. Northeast | 1.059 | 0.025 | 2.470 | 0.014 | 1.012 | 1.109 |
| South vs. Northeast | 1.011 | 0.024 | 0.480 | 0.630 | 0.966 | 1.060 |
| West vs. Northeast | 0.872 | 0.030 | -4.000 | 0.000 | 0.816 | 0.933 |
| Hospital referral region (suppressed) |  |  |  |  |  |  |
| Constant | 0.018 | 0.001 | -127.580 | 0.000 | 0.017 | 0.019 |
| **Practice Location** | **Margin** | **SE** | **t** | **P-value** | **LL CI** | **UL CI** |
| Isolated Rural | 0.071 | 0.000 | 302.210 | 0.000 | 0.070 | 0.071 |
| Small Town | 0.069 | 0.000 | 446.710 | 0.000 | 0.069 | 0.070 |
| Micropolitan | 0.066 | 0.000 | 606.920 | 0.000 | 0.065 | 0.066 |
| Metropolitan | 0.064 | 0.000 | 1601.730 | 0.000 | 0.064 | 0.064 |
| **Practice Location** | **Contrast** | **SE** | **t** | **P-value** |  |  |
| Small town vs. Isolated Rural | -0.035 | 0.008 | -4.650 | 0.000 |  |  |
| Micropolitan vs. Isolated Rural | -0.141 | 0.007 | -20.060 | 0.000 |  |  |
| Metropolitan vs. Isolated rural | -0.199 | 0.007 | -30.000 | 0.000 |  |  |
| Micropolitan vs. Small Town | -0.106 | 0.005 | -20.360 | 0.000 |  |  |
| Metropolitan vs. Small Town | -0.164 | 0.005 | -35.470 | 0.000 |  |  |
| Metropolitan vs. Micropolitan | -0.058 | 0.004 | -16.290 | 0.000 |  |  |

| **Potentially avoidable stay, acute composite** |  |  |  |  |  |  |
| --- | --- | --- | --- | --- | --- | --- |
| N | 27,710,872 |  |  |  |  |  |
| R^2^ | 0.192 |  |  |  |  |  |
|  | **OR** | **SE** | **Z Score** | **P-value** | **LL CI** | **UL CI** |
| Small Town vs. Isolated | 0.965 | 0.010 | -3.510 | 0.000 | 0.946 | 0.984 |
| Micropolitan vs. Isolated | 0.797 | 0.008 | -23.500 | 0.000 | 0.782 | 0.812 |
| Metropolitan vs. Isolated | 0.722 | 0.007 | -36.120 | 0.000 | 0.709 | 0.734 |
| Frail | 1.561 | 0.007 | 103.760 | 0.000 | 1.548 | 1.574 |
| Mean age | 1.045 | 0.000 | 151.090 | 0.000 | 1.044 | 1.045 |
| Under 65 | 1.675 | 0.014 | 63.470 | 0.000 | 1.648 | 1.702 |
| Over 85 | 1.119 | 0.006 | 20.500 | 0.000 | 1.107 | 1.132 |
| Female | 1.274 | 0.004 | 74.640 | 0.000 | 1.266 | 1.282 |
| White vs. Unknown | 1.245 | 0.026 | 10.390 | 0.000 | 1.195 | 1.298 |
| Black vs. Unknown | 1.218 | 0.026 | 9.100 | 0.000 | 1.168 | 1.271 |
| Other vs. Unknown | 1.164 | 0.030 | 5.790 | 0.000 | 1.105 | 1.225 |
| Asian vs. Unknown | 1.109 | 0.028 | 4.040 | 0.000 | 1.055 | 1.165 |
| Hispanic vs. Unknown | 1.384 | 0.034 | 13.410 | 0.000 | 1.320 | 1.452 |
| North American Native vs. Unknown | 1.769 | 0.049 | 20.480 | 0.000 | 1.675 | 1.868 |
| Disabled (original reason for Medicare eligibility) | 1.234 | 0.006 | 42.320 | 0.000 | 1.222 | 1.246 |
| Dual eligible for Medicaid | 1.105 | 0.004 | 25.350 | 0.000 | 1.097 | 1.114 |
| Nursing home | 1.933 | 0.008 | 162.270 | 0.000 | 1.918 | 1.949 |
| Died in the year | 1.043 | 0.005 | 9.170 | 0.000 | 1.033 | 1.052 |
| Median household income (area) | 1.000 | 0.000 | -12.510 | 0.000 | 1.000 | 1.000 |
| Residents under poverty level | 1.001 | 0.000 | 5.590 | 0.000 | 1.001 | 1.002 |
| Number of hierarchical condition categories | 1.351 | 0.001 | 413.500 | 0.000 | 1.349 | 1.352 |
| Coronary artery disease | 0.762 | 0.004 | -54.460 | 0.000 | 0.754 | 0.769 |
| Congestive heart failure | 1.100 | 0.004 | 23.330 | 0.000 | 1.091 | 1.109 |
| Diabetes | 1.020 | 0.004 | 5.640 | 0.000 | 1.013 | 1.027 |
| Cancer | 1.130 | 0.005 | 28.630 | 0.000 | 1.120 | 1.139 |
| Chronic obstructive pulmonary disease | 1.081 | 0.004 | 20.130 | 0.000 | 1.073 | 1.089 |
| End stage renal disease | 0.061 | 0.001 | -132.600 | 0.000 | 0.059 | 0.064 |
| Midwest vs. Northeast | 1.094 | 0.037 | 2.680 | 0.007 | 1.025 | 1.169 |
| South vs. Northeast | 1.037 | 0.035 | 1.080 | 0.280 | 0.970 | 1.109 |
| West vs. Northeast | 0.694 | 0.034 | -7.510 | 0.000 | 0.631 | 0.764 |
| Hospital referral region (suppressed) |  |  |  |  |  |  |
| Constant | 0.000 | 0.000 | -170.700 | 0.000 | 0.000 | 0.000 |
| **Practice Location** | **Margin** | **SE** | **t** | **P-value** | **LL CI** | **UL CI** |
| Isolated Rural | 0.022 | 0.000 | 130.720 | 0.000 | 0.022 | 0.022 |
| Small Town | 0.021 | 0.000 | 192.570 | 0.000 | 0.021 | 0.022 |
| Micropolitan | 0.018 | 0.000 | 242.390 | 0.000 | 0.018 | 0.018 |
| Metropolitan | 0.016 | 0.000 | 614.020 | 0.000 | 0.016 | 0.017 |
| **Practice Location** | **Contrast** | **SE** | **t** | **P-value** |  |  |
| Small town vs. Isolated Rural | -0.036 | 0.010 | -3.510 | 0.000 |  |  |
| Micropolitan vs. Isolated Rural | -0.227 | 0.010 | -23.500 | 0.000 |  |  |
| Metropolitan vs. Isolated rural | -0.326 | 0.009 | -36.120 | 0.000 |  |  |
| Micropolitan vs. Small Town | -0.191 | 0.007 | -26.490 | 0.000 |  |  |
| Metropolitan vs. Small Town | -0.291 | 0.006 | -45.900 | 0.000 |  |  |
| Metropolitan vs. Micropolitan | -0.100 | 0.005 | -19.390 | 0.000 |  |  |

| **Potentially avoidable stay, chronic composite** |  |  |  |  |  |  |
| --- | --- | --- | --- | --- | --- | --- |
| N | 27,710,872 |  |  |  |  |  |
| R^2^ | 0.361 |  |  |  |  |  |
|  | **OR** | **SE** | **Z Score** | **P-value** | **LL CI** | **UL CI** |
| Small Town vs. Isolated | 0.975 | 0.010 | -2.520 | 0.012 | 0.956 | 0.994 |
| Micropolitan vs. Isolated | 0.941 | 0.009 | -6.630 | 0.000 | 0.924 | 0.958 |
| Metropolitan vs. Isolated | 0.949 | 0.008 | -6.040 | 0.000 | 0.933 | 0.965 |
| Frail | 1.045 | 0.004 | 10.190 | 0.000 | 1.036 | 1.054 |
| Mean age | 1.011 | 0.000 | 47.270 | 0.000 | 1.011 | 1.012 |
| Under 65 | 1.510 | 0.010 | 63.930 | 0.000 | 1.491 | 1.530 |
| Over 85 | 1.264 | 0.006 | 46.460 | 0.000 | 1.252 | 1.277 |
| Female | 1.093 | 0.003 | 31.850 | 0.000 | 1.087 | 1.099 |
| White vs. Unknown | 1.115 | 0.019 | 6.440 | 0.000 | 1.079 | 1.152 |
| Black vs. Unknown | 1.449 | 0.025 | 21.430 | 0.000 | 1.401 | 1.499 |
| Other vs. Unknown | 1.151 | 0.024 | 6.630 | 0.000 | 1.104 | 1.200 |
| Asian vs. Unknown | 1.192 | 0.025 | 8.490 | 0.000 | 1.145 | 1.241 |
| Hispanic vs. Unknown | 1.467 | 0.029 | 19.610 | 0.000 | 1.412 | 1.524 |
| North American Native vs. Unknown | 1.439 | 0.034 | 15.540 | 0.000 | 1.375 | 1.507 |
| Disabled (original reason for Medicare eligibility) | 1.097 | 0.005 | 22.170 | 0.000 | 1.088 | 1.106 |
| Dual eligible for Medicaid | 0.941 | 0.003 | -17.380 | 0.000 | 0.935 | 0.948 |
| Nursing home | 1.004 | 0.004 | 1.020 | 0.307 | 0.997 | 1.011 |
| Died in the year | 1.120 | 0.005 | 27.680 | 0.000 | 1.111 | 1.129 |
| Median household income (area) | 1.000 | 0.000 | -16.930 | 0.000 | 1.000 | 1.000 |
| Residents under poverty level | 1.001 | 0.000 | 4.310 | 0.000 | 1.000 | 1.001 |
| Number of hierarchical condition categories | 1.262 | 0.001 | 354.590 | 0.000 | 1.260 | 1.264 |
| Coronary artery disease | 1.164 | 0.004 | 41.370 | 0.000 | 1.155 | 1.172 |
| Congestive heart failure | 6.013 | 0.020 | 531.220 | 0.000 | 5.973 | 6.053 |
| Diabetes | 1.523 | 0.004 | 143.010 | 0.000 | 1.514 | 1.532 |
| Cancer | 0.758 | 0.003 | -67.070 | 0.000 | 0.752 | 0.765 |
| Chronic obstructive pulmonary disease | 4.174 | 0.012 | 481.580 | 0.000 | 4.150 | 4.198 |
| End stage renal disease | 1.276 | 0.008 | 39.000 | 0.000 | 1.260 | 1.292 |
| Midwest vs. Northeast | 1.021 | 0.030 | 0.700 | 0.486 | 0.964 | 1.081 |
| South vs. Northeast | 1.038 | 0.031 | 1.250 | 0.212 | 0.979 | 1.100 |
| West vs. Northeast | 0.862 | 0.039 | -3.330 | 0.001 | 0.789 | 0.941 |
| Hospital referral region (suppressed) |  |  |  |  |  |  |
| Constant | 0.001 | 0.000 | -156.590 | 0.000 | 0.001 | 0.002 |
| **Practice Location** | **Margin** | **SE** | **t** | **P-value** | **LL CI** | **UL CI** |
| Isolated Rural | 0.028 | 0.000 | 161.870 | 0.000 | 0.028 | 0.028 |
| Small Town | 0.027 | 0.000 | 241.680 | 0.000 | 0.027 | 0.028 |
| Micropolitan | 0.027 | 0.000 | 329.980 | 0.000 | 0.027 | 0.027 |
| Metropolitan | 0.027 | 0.000 | 862.370 | 0.000 | 0.027 | 0.027 |
| **Practice Location** | **Contrast** | **SE** | **t** | **P-value** |  |  |
| Small town vs. Isolated Rural | -0.025 | 0.010 | -2.520 | 0.012 |  |  |
| Micropolitan vs. Isolated Rural | -0.061 | 0.009 | -6.630 | 0.000 |  |  |
| Metropolitan vs. Isolated rural | -0.053 | 0.009 | -6.040 | 0.000 |  |  |
| Micropolitan vs. Small Town | -0.036 | 0.007 | -5.360 | 0.000 |  |  |
| Metropolitan vs. Small Town | -0.028 | 0.006 | -4.600 | 0.000 |  |  |
| Metropolitan vs. Micropolitan | 0.009 | 0.005 | 1.880 | 0.060 |  |  |

| **30-day all-cause readmissions for medical discharges** |  |  |  |  |  |  |
| --- | --- | --- | --- | --- | --- | --- |
| N | 3,182,363 |  |  |  |  |  |
| R^2^ | 0.127 |  |  |  |  |  |
|  | **OR** | **SE** | **Z Score** | **P-value** | **LL CI** | **UL CI** |
| Small Town vs. Isolated | 0.970 | 0.011 | -2.750 | 0.006 | 0.949 | 0.991 |
| Micropolitan vs. Isolated | 0.822 | 0.009 | -18.870 | 0.000 | 0.806 | 0.839 |
| Metropolitan vs. Isolated | 0.816 | 0.008 | -20.940 | 0.000 | 0.800 | 0.831 |
| Frail | 1.138 | 0.005 | 30.280 | 0.000 | 1.129 | 1.148 |
| Mean age | 0.989 | 0.000 | -41.940 | 0.000 | 0.988 | 0.989 |
| Under 65 | 1.055 | 0.008 | 7.020 | 0.000 | 1.039 | 1.071 |
| Over 85 | 1.042 | 0.006 | 7.370 | 0.000 | 1.030 | 1.053 |
| Female | 1.046 | 0.003 | 14.010 | 0.000 | 1.039 | 1.053 |
| White vs. Unknown | 1.057 | 0.022 | 2.700 | 0.007 | 1.015 | 1.101 |
| Black vs. Unknown | 1.091 | 0.023 | 4.110 | 0.000 | 1.046 | 1.137 |
| Other vs. Unknown | 1.087 | 0.028 | 3.260 | 0.001 | 1.034 | 1.143 |
| Asian vs. Unknown | 1.092 | 0.027 | 3.510 | 0.000 | 1.040 | 1.147 |
| Hispanic vs. Unknown | 1.145 | 0.027 | 5.760 | 0.000 | 1.094 | 1.199 |
| North American Native vs. Unknown | 1.130 | 0.031 | 4.430 | 0.000 | 1.071 | 1.193 |
| Disabled (original reason for Medicare eligibility) | 1.005 | 0.005 | 1.070 | 0.284 | 0.996 | 1.015 |
| Dual eligible for Medicaid | 0.984 | 0.004 | -4.210 | 0.000 | 0.976 | 0.991 |
| Nursing home | 1.556 | 0.006 | 120.570 | 0.000 | 1.545 | 1.566 |
| Died in the year | 1.956 | 0.008 | 173.450 | 0.000 | 1.941 | 1.971 |
| Median household income (area) | 1.000 | 0.000 | -3.290 | 0.001 | 1.000 | 1.000 |
| Residents under poverty level | 1.001 | 0.000 | 3.590 | 0.000 | 1.000 | 1.001 |
| Number of hierarchical condition categories | 1.225 | 0.001 | 288.100 | 0.000 | 1.223 | 1.226 |
| Coronary artery disease | 0.977 | 0.004 | -5.460 | 0.000 | 0.969 | 0.985 |
| Congestive heart failure | 1.192 | 0.004 | 47.650 | 0.000 | 1.183 | 1.200 |
| Diabetes | 0.879 | 0.003 | -37.630 | 0.000 | 0.873 | 0.885 |
| Cancer | 0.976 | 0.004 | -5.730 | 0.000 | 0.968 | 0.984 |
| Chronic obstructive pulmonary disease | 1.068 | 0.004 | 18.700 | 0.000 | 1.061 | 1.075 |
| End stage renal disease | 1.145 | 0.008 | 20.380 | 0.000 | 1.130 | 1.160 |
| Midwest vs. Northeast | 0.964 | 0.032 | -1.100 | 0.273 | 0.903 | 1.029 |
| South vs. Northeast | 0.957 | 0.032 | -1.290 | 0.196 | 0.895 | 1.023 |
| West vs. Northeast | 0.809 | 0.040 | -4.260 | 0.000 | 0.734 | 0.892 |
| Hospital referral region (suppressed) |  |  |  |  |  |  |
| Constant | 0.156 | 0.007 | -38.670 | 0.000 | 0.142 | 0.171 |
| **Practice Location** | **Margin** | **SE** | **Z Score** | **P-value** | **LL CI** | **UL CI** |
| Isolated Rural | 0.207 | 0.001 | 155.600 | 0.000 | 0.205 | 0.210 |
| Small Town | 0.203 | 0.001 | 227.890 | 0.000 | 0.201 | 0.205 |
| Micropolitan | 0.181 | 0.001 | 289.010 | 0.000 | 0.180 | 0.182 |
| Metropolitan | 0.180 | 0.000 | 772.860 | 0.000 | 0.180 | 0.180 |
| **Practice Location** | **Contrast** | **SE** | **Z Score** | **P-value** |  |  |
| Small town vs. Isolated Rural | -0.030 | 0.011 | -2.750 | 0.006 |  |  |
| Micropolitan vs. Isolated Rural | -0.196 | 0.010 | -18.870 | 0.000 |  |  |
| Metropolitan vs. Isolated rural | -0.204 | 0.010 | -20.940 | 0.000 |  |  |
| Micropolitan vs. Small Town | -0.165 | 0.008 | -21.370 | 0.000 |  |  |
| Metropolitan vs. Small Town | -0.173 | 0.007 | -25.470 | 0.000 |  |  |
| Metropolitan vs. Micropolitan | -0.008 | 0.005 | -1.460 | 0.144 |  |  |

| **30-day all-cause readmissions for surgical discharges** |  |  |  |  |  |  |
| --- | --- | --- | --- | --- | --- | --- |
| N | 1,805,805 |  |  |  |  |  |
| R^2^ | 0.126 |  |  |  |  |  |
|  | **OR** | **SE** | **Z Score** | **P-value** | **LL CI** | **UL CI** |
| Small Town vs. Isolated | 1.035 | 0.019 | 1.840 | 0.065 | 0.998 | 1.073 |
| Micropolitan vs. Isolated | 0.938 | 0.016 | -3.730 | 0.000 | 0.907 | 0.970 |
| Metropolitan vs. Isolated | 0.912 | 0.015 | -5.680 | 0.000 | 0.884 | 0.941 |
| Frail | 1.061 | 0.007 | 8.640 | 0.000 | 1.047 | 1.075 |
| Mean age | 0.997 | 0.000 | -7.270 | 0.000 | 0.996 | 0.998 |
| Under 65 | 1.099 | 0.013 | 8.080 | 0.000 | 1.074 | 1.124 |
| Over 85 | 1.039 | 0.010 | 3.950 | 0.000 | 1.019 | 1.059 |
| Female | 0.965 | 0.005 | -7.280 | 0.000 | 0.955 | 0.974 |
| White vs. Unknown | 1.023 | 0.026 | 0.870 | 0.387 | 0.972 | 1.076 |
| Black vs. Unknown | 1.049 | 0.028 | 1.770 | 0.077 | 0.995 | 1.106 |
| Other vs. Unknown | 1.069 | 0.037 | 1.920 | 0.054 | 0.999 | 1.143 |
| Asian vs. Unknown | 1.109 | 0.039 | 2.960 | 0.003 | 1.036 | 1.187 |
| Hispanic vs. Unknown | 1.141 | 0.036 | 4.160 | 0.000 | 1.072 | 1.214 |
| North American Native vs. Unknown | 1.136 | 0.044 | 3.300 | 0.001 | 1.053 | 1.225 |
| Disabled (original reason for Medicare eligibility) | 1.041 | 0.008 | 5.280 | 0.000 | 1.025 | 1.056 |
| Dual eligible for Medicaid | 1.004 | 0.007 | 0.540 | 0.589 | 0.991 | 1.016 |
| Nursing home | 1.455 | 0.009 | 63.960 | 0.000 | 1.439 | 1.470 |
| Died in the year | 1.587 | 0.011 | 66.250 | 0.000 | 1.565 | 1.609 |
| Median household income (area) | 1.000 | 0.000 | -2.590 | 0.010 | 1.000 | 1.000 |
| Residents under poverty level | 1.000 | 0.000 | 1.050 | 0.294 | 1.000 | 1.001 |
| Number of hierarchical condition categories | 1.227 | 0.001 | 193.810 | 0.000 | 1.225 | 1.230 |
| Coronary artery disease | 0.995 | 0.006 | -0.780 | 0.433 | 0.982 | 1.008 |
| Congestive heart failure | 1.150 | 0.007 | 23.020 | 0.000 | 1.137 | 1.164 |
| Diabetes | 0.888 | 0.005 | -22.030 | 0.000 | 0.878 | 0.897 |
| Cancer | 0.986 | 0.006 | -2.280 | 0.023 | 0.974 | 0.998 |
| Chronic obstructive pulmonary disease | 0.942 | 0.006 | -9.940 | 0.000 | 0.931 | 0.953 |
| End stage renal disease | 1.172 | 0.011 | 16.470 | 0.000 | 1.150 | 1.194 |
| Midwest vs. Northeast | 1.096 | 0.058 | 1.740 | 0.083 | 0.988 | 1.215 |
| South vs. Northeast | 1.074 | 0.057 | 1.330 | 0.184 | 0.967 | 1.193 |
| West vs. Northeast | 0.982 | 0.075 | -0.240 | 0.812 | 0.845 | 1.141 |
| Hospital referral region (suppressed) |  |  |  |  |  |  |
| Constant | 0.064 | 0.005 | -37.610 | 0.000 | 0.056 | 0.074 |
| **Practice Location** | **Margin** | **SE** | **Z Score** | **P-value** | **LL CI** | **UL CI** |
| Isolated Rural | 0.132 | 0.002 | 83.090 | 0.000 | 0.129 | 0.135 |
| Small Town | 0.135 | 0.001 | 126.910 | 0.000 | 0.133 | 0.137 |
| Micropolitan | 0.126 | 0.001 | 172.160 | 0.000 | 0.124 | 0.127 |
| Metropolitan | 0.123 | 0.000 | 467.570 | 0.000 | 0.122 | 0.123 |
| **Practice Location** | **Contrast** | **SE** | **Z Score** | **P-value** |  |  |
| Small Town vs. Isolated Rural | 0.034 | 0.018 | 1.840 | 0.065 |  |  |
| Micropolitan vs. Isolated Rural | -0.064 | 0.017 | -3.730 | 0.000 |  |  |
| Metropolitan vs. Isolated Rural | -0.092 | 0.016 | -5.680 | 0.000 |  |  |
| Micropolitan vs. Small Town | -0.098 | 0.012 | -7.910 | 0.000 |  |  |
| Metropolitan vs. Small Town | -0.126 | 0.011 | -11.500 | 0.000 |  |  |
| Metropolitan vs. Micropolitan | -0.028 | 0.008 | -3.370 | 0.001 |  |  |

| **30-day all-cause readmissions, acute myocardial infarction** |  |  |  |  |  |  |
| --- | --- | --- | --- | --- | --- | --- |
| N | 156,964 |  |  |  |  |  |
| R^2^ | 0.113 |  |  |  |  |  |
|  | **OR** | **SE** | **Z Score** | **P-value** | **LL CI** | **UL CI** |
| Small Town vs. Isolated | 0.983 | 0.050 | -0.350 | 0.729 | 0.890 | 1.085 |
| Micropolitan vs. Isolated | 0.864 | 0.041 | -3.070 | 0.002 | 0.788 | 0.949 |
| Metropolitan vs. Isolated | 0.892 | 0.040 | -2.560 | 0.011 | 0.817 | 0.974 |
| Frail | 1.034 | 0.023 | 1.550 | 0.121 | 0.991 | 1.080 |
| Mean age | 1.004 | 0.001 | 2.700 | 0.007 | 1.001 | 1.000 |
| Under 65 | 1.157 | 0.042 | 3.990 | 0.000 | 1.077 | 1.242 |
| Over 85 | 0.968 | 0.027 | -1.150 | 0.250 | 0.916 | 1.023 |
| Female | 1.069 | 0.016 | 4.410 | 0.000 | 1.038 | 1.102 |
| White vs. Unknown | 1.278 | 0.130 | 2.420 | 0.016 | 1.048 | 1.559 |
| Black vs. Unknown | 1.251 | 0.130 | 2.150 | 0.031 | 1.020 | 1.535 |
| Other vs. Unknown | 1.353 | 0.164 | 2.500 | 0.012 | 1.067 | 1.716 |
| Asian vs. Unknown | 1.400 | 0.165 | 2.850 | 0.004 | 1.111 | 1.765 |
| Hispanic vs. Unknown | 1.518 | 0.175 | 3.620 | 0.000 | 1.211 | 1.902 |
| North American Native vs. Unknown | 1.173 | 0.157 | 1.190 | 0.233 | 0.902 | 1.525 |
| Disabled (original reason for Medicare eligibility) | 1.028 | 0.023 | 1.240 | 0.215 | 0.984 | 1.073 |
| Dual eligible for Medicaid | 1.023 | 0.019 | 1.190 | 0.235 | 0.985 | 1.061 |
| Nursing home | 1.370 | 0.025 | 17.370 | 0.000 | 1.322 | 1.420 |
| Died in the year | 1.567 | 0.029 | 24.280 | 0.000 | 1.511 | 1.625 |
| Median household income (area) | 1.000 | 0.000 | 1.100 | 0.272 | 1.000 | 1.000 |
| Residents under poverty level | 1.003 | 0.001 | 3.000 | 0.003 | 1.001 | 1.005 |
| Number of hierarchical condition categories | 1.183 | 0.004 | 47.080 | 0.000 | 1.174 | 1.191 |
| Coronary artery disease | 0.748 | 0.014 | -15.040 | 0.000 | 0.720 | 0.777 |
| Congestive heart failure | 1.584 | 0.031 | 23.800 | 0.000 | 1.525 | 1.645 |
| Diabetes | 0.975 | 0.016 | -1.510 | 0.131 | 0.944 | 1.007 |
| Cancer | 0.911 | 0.020 | -4.160 | 0.000 | 0.871 | 0.952 |
| Chronic obstructive pulmonary disease | 1.062 | 0.018 | 3.500 | 0.000 | 1.027 | 1.098 |
| End stage renal disease | 1.112 | 0.031 | 3.810 | 0.000 | 1.053 | 1.175 |
| Midwest vs. Northeast | 1.065 | 0.157 | 0.430 | 0.670 | 0.798 | 1.420 |
| South vs. Northeast | 1.113 | 0.164 | 0.730 | 0.465 | 0.834 | 1.486 |
| West vs. Northeast | 1.057 | 0.237 | 0.250 | 0.804 | 0.681 | 1.641 |
| Hospital referral region (suppressed) |  |  |  |  |  |  |
| Constant | 0.308 | 0.007 | -15.390 | 0.000 | 0.020 | 0.048 |
| **Practice Location** | **Margin** | **SE** | **Z Score** | **P-value** | **LL CI** | **UL CI** |
| Isolated Rural | 0.169 | 0.005 | 31.440 | 0.000 | 0.159 | 0.180 |
| Small Town | 0.167 | 0.004 | 46.290 | 0.000 | 0.160 | 0.174 |
| Micropolitan | 0.152 | 0.003 | 60.290 | 0.000 | 0.147 | 0.157 |
| Metropolitan | 0.156 | 0.001 | 151.950 | 0.000 | 0.154 | 0.158 |
| **Practice Location** | **Contrast** | **SE** | **Z Score** | **P-value** |  |  |
| Small Town vs. Isolated Rural | -0.018 | 0.051 | -0.350 | 0.729 |  |  |
| Micropolitan vs. Isolated Rural | -0.146 | 0.047 | -3.070 | 0.002 |  |  |
| Metropolitan vs. Isolated Rural | -0.114 | 0.045 | -2.560 | 0.011 |  |  |
| Micropolitan vs. Small Town | -0.128 | 0.035 | -3.640 | 0.000 |  |  |
| Metropolitan vs. Small Town | -0.097 | 0.031 | -3.100 | 0.002 |  |  |
| Metropolitan vs. Micropolitan | 0.032 | 0.024 | 1.290 | 0.197 |  |  |

| **30-day all-cause readmissions, congestive heart failure** |  |  |  |  |  |  |
| --- | --- | --- | --- | --- | --- | --- |
| N | 312,353 |  |  |  |  |  |
| R^2^ | 0.087 |  |  |  |  |  |
|  | **OR** | **SE** | **Z Score** | **P-value** | **LL CI** | **UL CI** |
| Small Town vs. Isolated | 0.971 | 0.032 | -0.880 | 0.381 | 0.910 | 1.037 |
| Micropolitan vs. Isolated | 0.887 | 0.028 | -3.850 | 0.000 | 0.835 | 0.943 |
| Metropolitan vs. Isolated | 0.863 | 0.025 | -5.040 | 0.000 | 0.815 | 0.914 |
| Frail | 1.053 | 0.013 | 4.350 | 0.000 | 1.029 | 1.078 |
| Mean age | 0.991 | 0.001 | -10.300 | 0.000 | 0.990 | 0.993 |
| Under 65 | 1.042 | 0.024 | 1.820 | 0.068 | 0.997 | 1.089 |
| Over 85 | 0.992 | 0.015 | -0.500 | 0.618 | 0.962 | 1.023 |
| Female | 1.014 | 0.009 | 1.540 | 0.124 | 0.996 | 1.033 |
| White vs. Unknown | 1.158 | 0.082 | 2.080 | 0.037 | 1.009 | 1.330 |
| Black vs. Unknown | 1.255 | 0.090 | 3.180 | 0.001 | 1.091 | 1.444 |
| Other vs. Unknown | 1.187 | 0.098 | 2.070 | 0.038 | 1.009 | 1.396 |
| Asian vs. Unknown | 1.184 | 0.096 | 2.090 | 0.037 | 1.010 | 1.389 |
| Hispanic vs. Unknown | 1.265 | 0.099 | 3.020 | 0.003 | 1.086 | 1.474 |
| North American Native vs. Unknown | 1.398 | 0.125 | 3.760 | 0.000 | 1.174 | 1.665 |
| Disabled (original reason for Medicare eligibility) | 1.007 | 0.013 | 0.570 | 0.571 | 0.982 | 1.034 |
| Dual eligible for Medicaid | 1.014 | 0.011 | 1.240 | 0.216 | 0.992 | 1.036 |
| Nursing home | 1.420 | 0.015 | 34.320 | 0.000 | 1.392 | 1.448 |
| Died in the year | 1.948 | 0.019 | 68.250 | 0.000 | 1.911 | 1.985 |
| Median household income (area) | 1.000 | 0.000 | -0.390 | 0.693 | 1.000 | 1.000 |
| Residents under poverty level | 1.002 | 0.001 | 3.480 | 0.000 | 1.001 | 1.003 |
| Number of hierarchical condition categories | 1.161 | 0.002 | 72.740 | 0.000 | 1.156 | 1.165 |
| Coronary artery disease | 1.108 | 0.011 | 10.120 | 0.000 | 1.086 | 1.130 |
| Congestive heart failure | 0.926 | 0.022 | -3.170 | 0.002 | 0.883 | 0.971 |
| Diabetes | 0.948 | 0.009 | -5.350 | 0.000 | 0.930 | 0.967 |
| Cancer | 0.892 | 0.012 | -8.660 | 0.000 | 0.869 | 0.915 |
| Chronic obstructive pulmonary disease | 1.166 | 0.011 | 15.880 | 0.000 | 1.144 | 1.189 |
| End stage renal disease | 1.121 | 0.017 | 7.610 | 0.000 | 1.089 | 1.155 |
| Midwest vs. Northeast | 1.036 | 0.096 | 0.390 | 0.698 | 0.865 | 1.242 |
| South vs. Northeast | 1.037 | 0.098 | 0.380 | 0.703 | 0.862 | 1.247 |
| West vs. Northeast | 0.835 | 0.122 | -1.230 | 0.217 | 0.627 | 1.112 |
| Hospital referral region (suppressed) |  |  |  |  |  |  |
| Constant | 0.128 | 0.019 | -13.960 | 0.000 | 0.096 | 0.171 |
| **Practice Location** | **Margin** | **SE** | **Z Score** | **P-value** | **LL CI** | **UL CI** |
| Isolated Rural | 0.257 | 0.005 | 52.540 | 0.000 | 0.247 | 0.266 |
| Small Town | 0.252 | 0.003 | 78.670 | 0.000 | 0.246 | 0.258 |
| Micropolitan | 0.237 | 0.002 | 103.950 | 0.000 | 0.232 | 0.241 |
| Metropolitan | 0.232 | 0.001 | 279.760 | 0.000 | 0.231 | 0.234 |
| **Practice Location** | **Contrast** | **SE** | **Z Score** | **P-value** |  |  |
| Small Town vs. Isolated Rural | -0.029 | 0.033 | -0.880 | 0.381 |  |  |
| Micropolitan vs. Isolated Rural | -0.120 | 0.031 | -3.850 | 0.000 |  |  |
| Metropolitan vs. Isolated Rural | -0.148 | 0.029 | -5.040 | 0.000 |  |  |
| Micropolitan vs. Small Town | -0.091 | 0.023 | -4.010 | 0.000 |  |  |
| Metropolitan vs. Small Town | -0.119 | 0.020 | -5.930 | 0.000 |  |  |
| Metropolitan vs. Micropolitan | -0.028 | 0.015 | -1.820 | 0.069 |  |  |

| **30-day all-cause readmissions, pneumonia** |  |  |  |  |  |  |
| --- | --- | --- | --- | --- | --- | --- |
| N | 375,263 |  |  |  |  |  |
| R^2^ | 0.099 |  |  |  |  |  |
|  | **OR** | **SE** | **Z Score** | **P-value** | **LL CI** | **UL CI** |
| Small Town vs. Isolated | 0.901 | 0.026 | -3.590 | 0.000 | 0.851 | 0.954 |
| Micropolitan vs. Isolated | 0.746 | 0.021 | -10.650 | 0.000 | 0.707 | 0.787 |
| Metropolitan vs. Isolated | 0.726 | 0.019 | -12.480 | 0.000 | 0.690 | 0.763 |
| Frail | 1.107 | 0.013 | 8.510 | 0.000 | 1.081 | 1.133 |
| Mean age | 0.989 | 0.001 | -13.520 | 0.000 | 0.988 | 0.991 |
| Under 65 | 1.045 | 0.023 | 1.960 | 0.050 | 1.000 | 1.091 |
| Over 85 | 0.995 | 0.016 | -0.300 | 0.766 | 0.965 | 1.026 |
| Female | 1.013 | 0.009 | 1.430 | 0.154 | 0.995 | 1.032 |
| White vs. Unknown | 1.010 | 0.065 | 0.150 | 0.881 | 0.890 | 1.145 |
| Black vs. Unknown | 1.064 | 0.070 | 0.950 | 0.343 | 0.936 | 1.210 |
| Other vs. Unknown | 1.096 | 0.085 | 1.190 | 0.235 | 0.942 | 1.276 |
| Asian vs. Unknown | 1.138 | 0.085 | 1.730 | 0.083 | 0.983 | 1.318 |
| Hispanic vs. Unknown | 1.132 | 0.081 | 1.730 | 0.084 | 0.984 | 1.303 |
| North American Native vs. Unknown | 1.007 | 0.081 | 0.080 | 0.935 | 0.859 | 1.180 |
| Disabled (original reason for Medicare eligibility) | 1.032 | 0.014 | 2.340 | 0.020 | 1.005 | 1.060 |
| Dual eligible for Medicaid | 0.967 | 0.011 | -3.050 | 0.002 | 0.947 | 0.988 |
| Nursing home | 1.472 | 0.016 | 36.310 | 0.000 | 1.441 | 1.503 |
| Died in the year | 1.803 | 0.018 | 57.930 | 0.000 | 1.767 | 1.839 |
| Median household income (area) | 1.000 | 0.000 | -3.970 | 0.000 | 1.000 | 1.000 |
| Residents under poverty level | 0.999 | 0.001 | -1.030 | 0.301 | 0.998 | 1.001 |
| Number of hierachical condition categories | 1.165 | 0.002 | 81.450 | 0.000 | 1.161 | 1.169 |
| Coronary artery disease | 0.990 | 0.012 | -0.800 | 0.425 | 0.966 | 1.014 |
| Congestive heart failure | 1.218 | 0.013 | 18.520 | 0.000 | 1.193 | 1.244 |
| Diabetes | 0.883 | 0.009 | -12.440 | 0.000 | 0.866 | 0.900 |
| Cancer | 1.011 | 0.012 | 0.880 | 0.378 | 0.987 | 1.034 |
| Chronic obstructive pulmonary disease | 1.115 | 0.011 | 11.080 | 0.000 | 1.094 | 1.136 |
| End stage renal disease | 1.024 | 0.019 | 1.280 | 0.200 | 0.988 | 1.061 |
| Midwest vs. Northeast | 1.106 | 0.105 | 1.070 | 0.286 | 0.919 | 1.331 |
| South vs. Northeast | 1.064 | 0.102 | 0.650 | 0.514 | 0.882 | 1.284 |
| West vs. Northeast | 0.896 | 0.125 | -0.790 | 0.430 | 0.681 | 1.177 |
| Hospital referral region (suppressed) |  |  |  |  |  |  |
| Constant | 0.154 | 0.022 | -13.300 | 0.000 | 0.117 | 0.203 |
| **Practice Location** | **Margin** | **SE** | **Z Score** | **P-value** | **LL CI** | **UL CI** |
| Isolated Rural | 0.214 | 0.004 | 57.800 | 0.000 | 0.206 | 0.221 |
| Small Town | 0.199 | 0.002 | 81.050 | 0.000 | 0.194 | 0.203 |
| Micropolitan | 0.173 | 0.002 | 98.400 | 0.000 | 0.169 | 0.176 |
| Metropolitan | 0.169 | 0.001 | 244.860 | 0.000 | 0.168 | 0.171 |
| **Practice Location** | **Contrast** | **SE** | **Z Score** | **P-value** |  |  |
| Small Town vs. Isolated Rural | -0.104 | 0.029 | -3.590 | 0.000 |  |  |
| Micropolitan vs. Isolated Rural | -0.293 | 0.027 | -10.650 | 0.000 |  |  |
| Metropolitan vs. Isolated Rural | -0.320 | 0.026 | -12.480 | 0.000 |  |  |
| Micropolitan vs. Small Town | -0.189 | 0.021 | -8.960 | 0.000 |  |  |
| Metropolitan vs. Small Town | -0.217 | 0.019 | -11.680 | 0.000 |  |  |
| Metropolitan vs. Micropolitan | -0.028 | 0.015 | -1.820 | 0.069 |  |  |

| **Emergency department visits discharged** |  |  |  |  |  |  |
| --- | --- | --- | --- | --- | --- | --- |
| N | 27,710,872 |  |  |  |  |  |
| R^2^ | 0.093 |  |  |  |  |  |
|  | **Coefficient** | **SE** | **t** | **P-value** | **LL CI** | **UL CI** |
| Small Town vs. Isolated | 0.035 | 0.002 | 19.660 | 0.000 | 0.031 | 0.038 |
| Micropolitan vs. Isolated | -0.009 | 0.002 | -5.430 | 0.000 | -0.012 | -0.006 |
| Metropolitan vs. Isolated | -0.081 | 0.002 | -52.440 | 0.000 | -0.084 | -0.078 |
| Frail | 0.203 | 0.001 | 169.270 | 0.000 | 0.201 | 0.205 |
| Mean age | -0.005 | 0.000 | -141.420 | 0.000 | -0.006 | -0.005 |
| Under 65 | 0.201 | 0.001 | 154.760 | 0.000 | 0.199 | 0.204 |
| Over 85 | 0.192 | 0.001 | 185.960 | 0.000 | 0.190 | 0.194 |
| Female | 0.069 | 0.000 | 141.880 | 0.000 | 0.068 | 0.070 |
| White vs. Unknown | 0.071 | 0.002 | 35.740 | 0.000 | 0.067 | 0.075 |
| Black vs. Unknown | 0.202 | 0.002 | 93.790 | 0.000 | 0.198 | 0.206 |
| Other vs. Unknown | 0.008 | 0.003 | 2.790 | 0.005 | 0.002 | 0.013 |
| Asian vs. Unknown | -0.091 | 0.003 | -33.140 | 0.000 | -0.096 | -0.085 |
| Hispanic vs. Unknown | 0.070 | 0.003 | 25.830 | 0.000 | 0.065 | 0.075 |
| North American Native vs. Unknown | 0.094 | 0.004 | 24.990 | 0.000 | 0.087 | 0.102 |
| Disabled (original reason for Medicare eligibility) | 0.071 | 0.001 | 77.610 | 0.000 | 0.069 | 0.073 |
| Dual eligible for Medicaid | 0.164 | 0.001 | 225.010 | 0.000 | 0.163 | 0.166 |
| Nursing home | -0.017 | 0.001 | -16.560 | 0.000 | -0.019 | -0.015 |
| Died in the year | -0.239 | 0.001 | -178.580 | 0.000 | -0.241 | -0.236 |
| Median household income (area) | 0.000 | 0.000 | -8.830 | 0.000 | 0.000 | 0.000 |
| Residents under poverty level | 0.001 | 0.000 | 34.030 | 0.000 | 0.001 | 0.001 |
| Number of hierarchical condition categories | 0.156 | 0.000 | 786.640 | 0.000 | 0.155 | 0.156 |
| Coronary artery disease | 0.091 | 0.001 | 76.520 | 0.000 | 0.089 | 0.093 |
| Congestive heart failure | -0.049 | 0.001 | -50.380 | 0.000 | -0.051 | -0.047 |
| Diabetes | -0.094 | 0.001 | -149.580 | 0.000 | -0.095 | -0.093 |
| Cancer | -0.089 | 0.001 | -103.220 | 0.000 | -0.091 | -0.088 |
| Chronic obstructive pulmonary disease | 0.087 | 0.001 | 98.420 | 0.000 | 0.085 | 0.088 |
| End stage renal disease | -0.017 | 0.002 | -7.520 | 0.000 | -0.022 | -0.013 |
| Midwest vs. Northeast | 0.146 | 0.006 | 25.140 | 0.000 | 0.135 | 0.157 |
| South vs. Northeast | 0.118 | 0.006 | 20.180 | 0.000 | 0.107 | 0.130 |
| West vs. Northeast | 0.178 | 0.008 | 22.340 | 0.000 | 0.163 | 0.194 |
| Hospital referral region (suppressed) |  |  |  |  |  |  |
| Constant | 0.334 | 0.007 | 44.810 | 0.000 | 0.319 | 0.348 |
| **Practice Location** | **Margin** | **SE** | **t** | **P-value** | **LL CI** | **UL CI** |
| Isolated Rural | 0.545 | 0.002 | 360.310 | 0.000 | 0.542 | 0.548 |
| Small Town | 0.580 | 0.001 | 563.940 | 0.000 | 0.578 | 0.582 |
| Micropolitan | 0.536 | 0.001 | 725.940 | 0.000 | 0.534 | 0.537 |
| Metropolitan | 0.463 | 0.000 | 1666.410 | 0.000 | 0.463 | 0.464 |
| **Practice Location** | **Contrast** | **SE** | **t** | **P-value** |  |  |
| Small town vs. Isolated Rural | 0.035 | 0.002 | 19.660 | 0.000 |  |  |
| Micropolitan vs. Isolated Rural | -0.009 | 0.002 | -5.430 | 0.000 |  |  |
| Metropolitan vs. Isolated rural | -0.081 | 0.002 | -52.440 | 0.000 |  |  |
| Micropolitan vs. Small Town | -0.044 | 0.001 | -36.010 | 0.000 |  |  |
| Metropolitan vs. Small Town | -0.116 | 0.001 | -107.290 | 0.000 |  |  |
| Metropolitan vs. Micropolitan | -0.072 | 0.001 | -89.200 | 0.000 |  |  |

| **Multiple emergency department visits, discharged** |  |  |  |  |  |  |
| --- | --- | --- | --- | --- | --- | --- |
| N | 27,710,872 |  |  |  |  |  |
| R^2^ | 0.251 |  |  |  |  |  |
|  | **OR** | **SE** | **Z Score** | **P-value** | **LL CI** | **UL CI** |
| Small Town vs. Isolated | 1.075 | 0.005 | 16.280 | 0.000 | 1.066 | 1.084 |
| Micropolitan vs. Isolated | 1.039 | 0.004 | 9.240 | 0.000 | 1.031 | 1.047 |
| Metropolitan vs. Isolated | 0.902 | 0.004 | -26.270 | 0.000 | 0.895 | 0.909 |
| Frail | 1.939 | 0.005 | 272.100 | 0.000 | 1.930 | 1.949 |
| Mean age | 0.999 | 0.000 | -15.340 | 0.000 | 0.998 | 0.999 |
| Under 65 | 1.606 | 0.005 | 156.750 | 0.000 | 1.596 | 1.615 |
| Over 85 | 1.587 | 0.004 | 191.980 | 0.000 | 1.579 | 1.594 |
| Female | 1.228 | 0.002 | 159.850 | 0.000 | 1.225 | 1.231 |
| White vs. Unknown | 1.281 | 0.008 | 37.920 | 0.000 | 1.264 | 1.297 |
| Black vs. Unknown | 1.736 | 0.012 | 81.260 | 0.000 | 1.713 | 1.760 |
| Other vs. Unknown | 1.093 | 0.010 | 10.140 | 0.000 | 1.075 | 1.112 |
| Asian vs. Unknown | 0.928 | 0.008 | -8.660 | 0.000 | 0.912 | 0.944 |
| Hispanic vs. Unknown | 1.584 | 0.012 | 58.910 | 0.000 | 1.560 | 1.609 |
| North American Native vs. Unknown | 1.311 | 0.013 | 26.820 | 0.000 | 1.285 | 1.337 |
| Disabled (original reason for Medicare eligibility) | 1.234 | 0.003 | 97.380 | 0.000 | 1.228 | 1.239 |
| Dual eligible for Medicaid | 1.218 | 0.002 | 118.000 | 0.000 | 1.214 | 1.222 |
| Nursing home | 1.358 | 0.003 | 151.480 | 0.000 | 1.352 | 1.363 |
| Died in the year | 1.055 | 0.003 | 19.610 | 0.000 | 1.049 | 1.061 |
| Median household income (area) | 1.000 | 0.000 | -31.610 | 0.000 | 1.000 | 1.000 |
| Residents under poverty level | 1.002 | 0.000 | 20.560 | 0.000 | 1.002 | 1.002 |
| Number of hierarchical condition categories | 1.682 | 0.001 | 1164.590 | 0.000 | 1.681 | 1.683 |
| Coronary artery disease | 1.291 | 0.003 | 106.340 | 0.000 | 1.284 | 1.297 |
| Congestive heart failure | 0.985 | 0.002 | -7.750 | 0.000 | 0.981 | 0.989 |
| Diabetes | 0.724 | 0.001 | -215.210 | 0.000 | 0.722 | 0.726 |
| Cancer | 0.864 | 0.002 | -73.200 | 0.000 | 0.860 | 0.867 |
| Chronic obstructive pulmonary disease | 1.114 | 0.002 | 60.070 | 0.000 | 1.110 | 1.118 |
| End stage renal disease | 0.687 | 0.003 | -82.100 | 0.000 | 0.681 | 0.693 |
| Midwest vs. Northeast | 1.334 | 0.019 | 20.230 | 0.000 | 1.298 | 1.372 |
| South vs. Northeast | 1.297 | 0.019 | 17.950 | 0.000 | 1.261 | 1.334 |
| West vs. Northeast | 1.523 | 0.031 | 20.960 | 0.000 | 1.464 | 1.584 |
| Hospital referral region (suppressed) |  |  |  |  |  |  |
| Constant | 0.028 | 0.001 | -188.200 | 0.000 | 0.027 | 0.029 |
| **Practice Location** | **Margin** | **SE** | **t** | **P-value** | **LL CI** | **UL CI** |
| Isolated Rural | 0.159 | 0.000 | 430.370 | 0.000 | 0.159 | 0.160 |
| Small Town | 0.167 | 0.000 | 655.660 | 0.000 | 0.166 | 0.167 |
| Micropolitan | 0.163 | 0.000 | 898.180 | 0.000 | 0.163 | 0.164 |
| Metropolitan | 0.150 | 0.000 | 2221.080 | 0.000 | 0.150 | 0.150 |
| **Practice Location** | **Contrast** | **SE** | **t** | **P-value** |  |  |
| Small town vs. Isolated Rural | 0.072 | 0.004 | 16.280 | 0.000 |  |  |
| Micropolitan vs. Isolated Rural | 0.038 | 0.004 | 9.240 | 0.000 |  |  |
| Metropolitan vs. Isolated rural | -0.103 | 0.004 | -26.270 | 0.000 |  |  |
| Micropolitan vs. Small Town | -0.034 | 0.003 | -11.350 | 0.000 |  |  |
| Metropolitan vs. Small Town | -0.175 | 0.003 | -65.520 | 0.000 |  |  |
| Metropolitan vs. Micropolitan | -0.141 | 0.002 | -69.380 | 0.000 |  |  |

| **Emergency department visits that were necessary, but preventable** |  |  |  |  |  |  |
| --- | --- | --- | --- | --- | --- | --- |
| N | 6,222,441 |  |  |  |  |  |
| R^2^ | 0.037 |  |  |  |  |  |
|  | **Coefficient** | **SE** | **t** | **P-value** | **LL CI** | **UL CI** |
| Small Town vs. Isolated | -0.001 | 0.001 | -0.910 | 0.363 | -0.002 | 0.001 |
| Micropolitan vs. Isolated | -0.006 | 0.001 | -8.620 | 0.000 | -0.008 | -0.005 |
| Metropolitan vs. Isolated | -0.009 | 0.001 | -12.620 | 0.000 | -0.010 | -0.007 |
| Frail | -0.009 | 0.000 | -20.650 | 0.000 | -0.010 | -0.008 |
| Mean age | 0.000 | 0.000 | 16.000 | 0.000 | 0.000 | 0.000 |
| Under 65 | 0.006 | 0.001 | 10.870 | 0.000 | 0.005 | 0.007 |
| Over 85 | 0.015 | 0.000 | 38.140 | 0.000 | 0.015 | 0.016 |
| Female | -0.007 | 0.000 | -30.940 | 0.000 | -0.007 | -0.006 |
| White vs. Unknown | 0.004 | 0.001 | 3.700 | 0.000 | 0.002 | 0.006 |
| Black vs. Unknown | 0.002 | 0.001 | 1.610 | 0.106 | 0.000 | 0.004 |
| Other vs. Unknown | 0.010 | 0.002 | 6.580 | 0.000 | 0.007 | 0.013 |
| Asian vs. Unknown | 0.015 | 0.001 | 10.220 | 0.000 | 0.012 | 0.018 |
| Hispanic vs. Unknown | 0.006 | 0.001 | 4.560 | 0.000 | 0.003 | 0.009 |
| North American Native vs. Unknown | 0.012 | 0.002 | 6.750 | 0.000 | 0.008 | 0.015 |
| Disabled (original reason for Medicare eligibility) | 0.005 | 0.000 | 12.750 | 0.000 | 0.004 | 0.005 |
| Dual eligible for Medicaid | 0.004 | 0.000 | 14.660 | 0.000 | 0.004 | 0.005 |
| Nursing home | 0.008 | 0.000 | 23.350 | 0.000 | 0.007 | 0.009 |
| Died in the year | 0.037 | 0.000 | 85.260 | 0.000 | 0.036 | 0.038 |
| Median household income (area) | 0.000 | 0.000 | -0.970 | 0.333 | 0.000 | 0.000 |
| Residents under poverty level | 0.000 | 0.000 | 2.360 | 0.018 | 0.000 | 0.000 |
| Number of hierarchical condition categories | 0.001 | 0.000 | 18.220 | 0.000 | 0.001 | 0.001 |
| Coronary artery disease | -0.019 | 0.000 | -46.350 | 0.000 | -0.020 | -0.018 |
| Congestive heart failure | 0.079 | 0.000 | 237.100 | 0.000 | 0.078 | 0.080 |
| Diabetes | 0.044 | 0.000 | 171.690 | 0.000 | 0.043 | 0.044 |
| Cancer | -0.008 | 0.000 | -23.050 | 0.000 | -0.009 | -0.007 |
| Chronic obstructive pulmonary disease | 0.035 | 0.000 | 110.270 | 0.000 | 0.034 | 0.035 |
| End stage renal disease | -0.001 | 0.001 | -1.050 | 0.294 | -0.002 | 0.001 |
| Midwest vs. Northeast | 0.000 | 0.002 | 0.030 | 0.977 | -0.005 | 0.005 |
| South vs. Northeast | 0.001 | 0.002 | 0.480 | 0.630 | -0.004 | 0.006 |
| West vs. Northeast | -0.001 | 0.003 | -0.330 | 0.744 | -0.008 | 0.006 |
| Hospital referral region (suppressed) |  |  |  |  |  |  |
| Constant | 0.040 | 0.003 | 12.590 | 0.000 | 0.034 | 0.047 |
| **Practice Location** | **Margin** | **SE** | **t** | **P-value** | **LL CI** | **UL CI** |
| Isolated Rural | 0.108 | 0.001 | 164.200 | 0.000 | 0.107 | 0.109 |
| Small Town | 0.107 | 0.000 | 247.080 | 0.000 | 0.107 | 0.108 |
| Micropolitan | 0.102 | 0.000 | 323.010 | 0.000 | 0.101 | 0.103 |
| Metropolitan | 0.100 | 0.000 | 795.790 | 0.000 | 0.099 | 0.100 |
| **Practice Location** | **Contrast** | **SE** | **t** | **P-value** |  |  |
| Small town vs. Isolated Rural | -0.001 | 0.001 | -0.910 | 0.363 |  |  |
| Micropolitan vs. Isolated Rural | -0.006 | 0.001 | -8.620 | 0.000 |  |  |
| Metropolitan vs. Isolated rural | -0.009 | 0.001 | -12.620 | 0.000 |  |  |
| Micropolitan vs. Small Town | -0.005 | 0.001 | -10.560 | 0.000 |  |  |
| Metropolitan vs. Small Town | -0.008 | 0.000 | -17.000 | 0.000 |  |  |
| Metropolitan vs. Micropolitan | -0.002 | 0.000 | -6.790 | 0.000 |  |  |

| **Emergency department visits that were unnecessary, but emergent** |  |  |  |  |  |  |
| --- | --- | --- | --- | --- | --- | --- |
| N | 6,222,441 |  |  |  |  |  |
| R^2^ | 0.008 |  |  |  |  |  |
|  | **Coefficient** | **SE** | **t** | **P-value** | **LL CI** | **UL CI** |
| Small Town vs. Isolated | 0.004 | 0.001 | 3.460 | 0.001 | 0.002 | 0.007 |
| Micropolitan vs. Isolated | 0.000 | 0.001 | -0.160 | 0.873 | -0.002 | 0.002 |
| Metropolitan vs. Isolated | -0.009 | 0.001 | -8.710 | 0.000 | -0.012 | -0.007 |
| Frail | 0.009 | 0.001 | 12.940 | 0.000 | 0.007 | 0.010 |
| Mean age | -0.001 | 0.000 | -38.450 | 0.000 | -0.001 | -0.001 |
| Under 65 | 0.001 | 0.001 | 1.230 | 0.219 | -0.001 | 0.003 |
| Over 85 | 0.013 | 0.001 | 19.510 | 0.000 | 0.011 | 0.014 |
| Female | 0.021 | 0.000 | 61.370 | 0.000 | 0.021 | 0.022 |
| White vs. Unknown | 0.009 | 0.002 | 4.980 | 0.000 | 0.005 | 0.013 |
| Black vs. Unknown | -0.009 | 0.002 | -4.730 | 0.000 | -0.013 | -0.005 |
| Other vs. Unknown | 0.001 | 0.002 | 0.550 | 0.582 | -0.003 | 0.006 |
| Asian vs. Unknown | -0.006 | 0.002 | -2.390 | 0.017 | -0.010 | -0.001 |
| Hispanic vs. Unknown | 0.019 | 0.002 | 8.710 | 0.000 | 0.014 | 0.023 |
| North American Native vs. Unknown | 0.017 | 0.003 | 6.150 | 0.000 | 0.012 | 0.022 |
| Disabled (original reason for Medicare eligibility) | 0.014 | 0.001 | 25.330 | 0.000 | 0.013 | 0.015 |
| Dual eligible for Medicaid | 0.014 | 0.000 | 31.590 | 0.000 | 0.013 | 0.015 |
| Nursing home | -0.006 | 0.001 | -11.740 | 0.000 | -0.007 | -0.005 |
| Died in the year | -0.014 | 0.001 | -19.740 | 0.000 | -0.015 | -0.012 |
| Median household income (area) | 0.000 | 0.000 | -7.370 | 0.000 | 0.000 | 0.000 |
| Residents under poverty level | 0.000 | 0.000 | -2.520 | 0.012 | 0.000 | 0.000 |
| Number of hierarchical condition categories | -0.004 | 0.000 | -34.960 | 0.000 | -0.004 | -0.004 |
| Coronary artery disease | 0.015 | 0.001 | 23.070 | 0.000 | 0.014 | 0.016 |
| Congestive heart failure | -0.037 | 0.001 | -70.150 | 0.000 | -0.038 | -0.036 |
| Diabetes | -0.004 | 0.000 | -9.750 | 0.000 | -0.005 | -0.003 |
| Cancer | 0.015 | 0.001 | 26.640 | 0.000 | 0.014 | 0.016 |
| Chronic obstructive pulmonary disease | -0.006 | 0.000 | -11.200 | 0.000 | -0.007 | -0.005 |
| End stage renal disease | -0.050 | 0.001 | -47.280 | 0.000 | -0.052 | -0.048 |
| Midwest vs. Northeast | 0.006 | 0.004 | 1.490 | 0.137 | -0.002 | 0.013 |
| South vs. Northeast | 0.002 | 0.004 | 0.460 | 0.646 | -0.006 | 0.009 |
| West vs. Northeast | -0.010 | 0.005 | -1.800 | 0.072 | -0.020 | 0.001 |
| Hospital referral region (suppressed) |  |  |  |  |  |  |
| Constant | 0.407 | 0.005 | 79.890 | 0.000 | 0.397 | 0.417 |
| **Practice Location** | **Margin** | **SE** | **t** | **P-value** | **LL CI** | **UL CI** |
| Isolated Rural | 0.335 | 0.001 | 319.040 | 0.000 | 0.333 | 0.337 |
| Small Town | 0.339 | 0.001 | 489.300 | 0.000 | 0.337 | 0.340 |
| Micropolitan | 0.334 | 0.001 | 665.210 | 0.000 | 0.333 | 0.335 |
| Metropolitan | 0.325 | 0.000 | 1631.750 | 0.000 | 0.325 | 0.326 |
| **Practice Location** | **Contrast** | **SE** | **t** | **P-value** |  |  |
| Small town vs. Isolated Rural | 0.004 | 0.001 | 3.460 | 0.001 |  |  |
| Micropolitan vs. Isolated Rural | 0.000 | 0.001 | -0.160 | 0.873 |  |  |
| Metropolitan vs. Isolated rural | -0.009 | 0.001 | -8.710 | 0.000 |  |  |
| Micropolitan vs. Small Town | -0.004 | 0.001 | -5.340 | 0.000 |  |  |
| Metropolitan vs. Small Town | -0.014 | 0.001 | -18.530 | 0.000 |  |  |
| Metropolitan vs. Micropolitan | -0.009 | 0.001 | -16.520 | 0.000 |  |  |

| **Emergency department visits that were unnecessary and nonemergent** |  |  |  |  |  |  |
| --- | --- | --- | --- | --- | --- | --- |
| N | 6,222,441 |  |  |  |  |  |
| R^2^ | 0.021 |  |  |  |  |  |
|  | **Coefficient** | **SE** | **t** | **P-value** | **LL CI** | **UL CI** |
| Small Town vs. Isolated | 0.000 | 0.001 | 0.130 | 0.898 | -0.002 | 0.002 |
| Micropolitan vs. Isolated | 0.000 | 0.001 | 0.100 | 0.922 | -0.002 | 0.002 |
| Metropolitan vs. Isolated | 0.002 | 0.001 | 2.000 | 0.046 | 0.000 | 0.004 |
| Frail | 0.021 | 0.001 | 33.500 | 0.000 | 0.020 | 0.022 |
| Mean age | -0.001 | 0.000 | -32.180 | 0.000 | -0.001 | -0.001 |
| Under 65 | 0.021 | 0.001 | 27.610 | 0.000 | 0.020 | 0.023 |
| Over 85 | 0.004 | 0.001 | 7.610 | 0.000 | 0.003 | 0.006 |
| Female | 0.035 | 0.000 | 109.200 | 0.000 | 0.034 | 0.036 |
| White vs. Unknown | 0.003 | 0.002 | 1.650 | 0.099 | -0.001 | 0.006 |
| Black vs. Unknown | 0.060 | 0.002 | 34.680 | 0.000 | 0.057 | 0.063 |
| Other vs. Unknown | 0.016 | 0.002 | 7.410 | 0.000 | 0.012 | 0.021 |
| Asian vs. Unknown | 0.019 | 0.002 | 8.580 | 0.000 | 0.014 | 0.023 |
| Hispanic vs. Unknown | 0.027 | 0.002 | 13.640 | 0.000 | 0.023 | 0.031 |
| North American Native vs. Unknown | 0.009 | 0.003 | 3.500 | 0.000 | 0.004 | 0.014 |
| Disabled (original reason for Medicare eligibility) | 0.006 | 0.001 | 10.730 | 0.000 | 0.005 | 0.007 |
| Dual eligible for Medicaid | 0.009 | 0.000 | 20.990 | 0.000 | 0.008 | 0.010 |
| Nursing home | -0.020 | 0.001 | -40.490 | 0.000 | -0.021 | -0.019 |
| Died in the year | -0.027 | 0.001 | -42.120 | 0.000 | -0.028 | -0.025 |
| Median household income (area) | 0.000 | 0.000 | -6.180 | 0.000 | 0.000 | 0.000 |
| Residents under poverty level | 0.000 | 0.000 | 8.770 | 0.000 | 0.000 | 0.000 |
| Number of hierarchical condition categories | -0.009 | 0.000 | -91.660 | 0.000 | -0.009 | -0.009 |
| Coronary artery disease | -0.008 | 0.001 | -14.250 | 0.000 | -0.010 | -0.007 |
| Congestive heart failure | -0.026 | 0.000 | -53.400 | 0.000 | -0.027 | -0.025 |
| Diabetes | -0.009 | 0.000 | -24.650 | 0.000 | -0.010 | -0.008 |
| Cancer | -0.001 | 0.001 | -2.810 | 0.005 | -0.002 | 0.000 |
| Chronic obstructive pulmonary disease | -0.013 | 0.000 | -27.700 | 0.000 | -0.014 | -0.012 |
| End stage renal disease | 0.023 | 0.001 | 23.960 | 0.000 | 0.021 | 0.025 |
| Midwest vs. Northeast | 0.007 | 0.003 | 2.080 | 0.037 | 0.000 | 0.014 |
| South vs. Northeast | 0.000 | 0.004 | 0.100 | 0.920 | -0.007 | 0.007 |
| West vs. Northeast | -0.008 | 0.005 | -1.630 | 0.102 | -0.018 | 0.002 |
| Hospital referral region (suppressed) |  |  |  |  |  |  |
| Constant | 0.290 | 0.005 | 61.890 | 0.000 | 0.281 | 0.299 |
| **Practice Location** | **Margin** | **SE** | **t** | **P-value** | **LL CI** | **UL CI** |
| Isolated Rural | 0.250 | 0.001 | 258.710 | 0.000 | 0.248 | 0.252 |
| Small Town | 0.250 | 0.001 | 392.060 | 0.000 | 0.249 | 0.251 |
| Micropolitan | 0.250 | 0.000 | 539.930 | 0.000 | 0.249 | 0.251 |
| Metropolitan | 0.252 | 0.000 | 1372.190 | 0.000 | 0.252 | 0.252 |
| **Practice Location** | **Contrast** | **SE** | **t** | **P-value** |  |  |
| Small town vs. Isolated Rural | 0.000 | 0.001 | 0.130 | 0.898 |  |  |
| Micropolitan vs. Isolated Rural | 0.000 | 0.001 | 0.100 | 0.922 |  |  |
| Metropolitan vs. Isolated rural | 0.002 | 0.001 | 2.000 | 0.046 |  |  |
| Micropolitan vs. Small Town | 0.000 | 0.001 | -0.060 | 0.956 |  |  |
| Metropolitan vs. Small Town | 0.002 | 0.001 | 2.710 | 0.007 |  |  |
| Metropolitan vs. Micropolitan | 0.002 | 0.001 | 3.660 | 0.000 |  |  |

| **Died** |  |  |  |  |  |  |
| --- | --- | --- | --- | --- | --- | --- |
| N | 27,710,872 |  |  |  |  |  |
| R^2^ | 0.283 |  |  |  |  |  |
|  | **OR** | **SE** | **Z Score** | **P-value** | **LL CI** | **UL CI** |
| Small Town vs. Isolated | 0.995 | 0.008 | -0.600 | 0.548 | 0.980 | 1.011 |
| Micropolitan vs. Isolated | 0.973 | 0.007 | -3.740 | 0.000 | 0.959 | 0.987 |
| Metropolitan vs. Isolated | 0.961 | 0.007 | -5.720 | 0.000 | 0.948 | 0.974 |
| Frail | 0.598 | 0.002 | -144.000 | 0.000 | 0.594 | 0.602 |
| Mean age | 1.074 | 0.000 | 329.410 | 0.000 | 1.073 | 1.074 |
| Under 65 | 1.621 | 0.010 | 77.950 | 0.000 | 1.601 | 1.640 |
| Over 85 | 1.361 | 0.005 | 79.580 | 0.000 | 1.351 | 1.372 |
| Female | 0.802 | 0.002 | -97.140 | 0.000 | 0.798 | 0.806 |
| White vs. Unknown | 1.167 | 0.017 | 10.510 | 0.000 | 1.134 | 1.202 |
| Black vs. Unknown | 1.022 | 0.016 | 1.400 | 0.161 | 0.992 | 1.053 |
| Other vs. Unknown | 1.062 | 0.019 | 3.290 | 0.001 | 1.024 | 1.100 |
| Asian vs. Unknown | 0.974 | 0.017 | -1.500 | 0.135 | 0.941 | 1.008 |
| Hispanic vs. Unknown | 0.979 | 0.017 | -1.200 | 0.231 | 0.947 | 1.013 |
| North American Native vs. Unknown | 1.298 | 0.027 | 12.660 | 0.000 | 1.247 | 1.352 |
| Disabled (original reason for Medicare eligibility) | 1.265 | 0.005 | 63.770 | 0.000 | 1.256 | 1.274 |
| Dual eligible for Medicaid | 1.449 | 0.004 | 130.040 | 0.000 | 1.441 | 1.457 |
| Nursing home | 2.744 | 0.008 | 367.450 | 0.000 | 2.730 | 2.759 |
| Median household income (area) | 1.000 | 0.000 | -13.780 | 0.000 | 1.000 | 1.000 |
| Residents under poverty level | 1.000 | 0.000 | 0.450 | 0.652 | 1.000 | 1.000 |
| Number of hierarchical condition categories | 1.451 | 0.001 | 681.270 | 0.000 | 1.449 | 1.452 |
| Coronary artery disease | 0.832 | 0.003 | -50.950 | 0.000 | 0.826 | 0.838 |
| Congestive heart failure | 1.001 | 0.003 | 0.280 | 0.777 | 0.995 | 1.007 |
| Diabetes | 0.574 | 0.002 | -209.920 | 0.000 | 0.571 | 0.577 |
| Cancer | 2.078 | 0.006 | 263.910 | 0.000 | 2.066 | 2.089 |
| Chronic obstructive pulmonary disease | 0.860 | 0.002 | -52.180 | 0.000 | 0.855 | 0.865 |
| End stage renal disease | 1.160 | 0.007 | 23.120 | 0.000 | 1.145 | 1.174 |
| Midwest vs. Northeast | 0.954 | 0.024 | -1.820 | 0.068 | 0.908 | 1.004 |
| South vs. Northeast | 0.992 | 0.026 | -0.300 | 0.761 | 0.943 | 1.044 |
| West vs. Northeast | 0.962 | 0.034 | -1.090 | 0.277 | 0.897 | 1.032 |
| Hospital referral region (suppressed) |  |  |  |  |  |  |
| Constant | 0.000 | 0.000 | -265.730 | 0.000 | 0.000 | 0.000 |
| **Practice Location** | **Margin** | **SE** | **t** | **P-value** | **LL CI** | **UL CI** |
| Isolated Rural | 0.040 | 0.000 | 196.360 | 0.000 | 0.040 | 0.041 |
| Small Town | 0.040 | 0.000 | 288.700 | 0.000 | 0.040 | 0.040 |
| Micropolitan | 0.039 | 0.000 | 391.790 | 0.000 | 0.039 | 0.040 |
| Metropolitan | 0.039 | 0.000 | 1017.990 | 0.000 | 0.039 | 0.039 |
| **Practice Location** | **Contrast** | **SE** | **t** | **P-value** |  |  |
| Small town vs. Isolated Rural | -0.005 | 0.008 | -0.600 | 0.548 |  |  |
| Micropolitan vs. Isolated Rural | -0.027 | 0.007 | -3.740 | 0.000 |  |  |
| Metropolitan vs. Isolated rural | -0.039 | 0.007 | -5.720 | 0.000 |  |  |
| Micropolitan vs. Small Town | -0.023 | 0.005 | -4.170 | 0.000 |  |  |
| Metropolitan vs. Small Town | -0.035 | 0.005 | -7.190 | 0.000 |  |  |
| Metropolitan vs. Micropolitan | -0.012 | 0.004 | -3.240 | 0.001 |  |  |

| **Diabetics who had a blood lipids test** |  |  |  |  |  |  |
| --- | --- | --- | --- | --- | --- | --- |
| N | 7,523,294 |  |  |  |  |  |
| R^2^ | 0.095 |  |  |  |  |  |
|  | **OR** | **SE** | **Z Score** | **P-value** | **LL CI** | **UL CI** |
| Small Town vs. Isolated | 1.013 | 0.007 | 1.980 | 0.047 | 1.000 | 1.026 |
| Micropolitan vs. Isolated | 1.038 | 0.006 | 6.250 | 0.000 | 1.026 | 1.051 |
| Metropolitan vs. Isolated | 1.034 | 0.006 | 5.850 | 0.000 | 1.022 | 1.045 |
| Frail | 1.023 | 0.004 | 6.000 | 0.000 | 1.015 | 1.031 |
| Mean age | 0.996 | 0.000 | -26.630 | 0.000 | 0.995 | 0.996 |
| Under 65 | 0.786 | 0.003 | -55.780 | 0.000 | 0.779 | 0.793 |
| Over 85 | 0.602 | 0.002 | -132.160 | 0.000 | 0.597 | 0.606 |
| Female | 1.071 | 0.002 | 36.480 | 0.000 | 1.067 | 1.075 |
| White vs. Unknown | 0.941 | 0.009 | -6.500 | 0.000 | 0.924 | 0.959 |
| Black vs. Unknown | 0.747 | 0.007 | -30.290 | 0.000 | 0.733 | 0.761 |
| Other vs. Unknown | 1.049 | 0.012 | 4.120 | 0.000 | 1.026 | 1.074 |
| Asian vs. Unknown | 1.111 | 0.013 | 9.240 | 0.000 | 1.086 | 1.136 |
| Hispanic vs. Unknown | 0.890 | 0.010 | -10.730 | 0.000 | 0.871 | 0.909 |
| North American Native vs. Unknown | 0.277 | 0.003 | -101.880 | 0.000 | 0.271 | 0.284 |
| Disabled (original reason for Medicare eligibility) | 0.831 | 0.002 | -64.430 | 0.000 | 0.826 | 0.836 |
| Dual eligible for Medicaid | 0.936 | 0.002 | -27.470 | 0.000 | 0.931 | 0.940 |
| Nursing home | 0.531 | 0.002 | -213.550 | 0.000 | 0.528 | 0.534 |
| Died in the year | 0.261 | 0.001 | -361.210 | 0.000 | 0.259 | 0.263 |
| Median household income (area) | 1.000 | 0.000 | 4.890 | 0.000 | 1.000 | 1.000 |
| Residents under poverty level | 0.997 | 0.000 | -27.110 | 0.000 | 0.996 | 0.997 |
| Number of hierarchical condition categories | 0.945 | 0.001 | -97.180 | 0.000 | 0.944 | 0.946 |
| Coronary artery disease | 1.303 | 0.005 | 74.170 | 0.000 | 1.294 | 1.313 |
| Congestive heart failure | 0.979 | 0.003 | -7.380 | 0.000 | 0.973 | 0.984 |
| Diabetes | 2.635 | 0.007 | 351.160 | 0.000 | 2.621 | 2.650 |
| Cancer | 0.905 | 0.003 | -32.720 | 0.000 | 0.899 | 0.910 |
| Chronic obstructive pulmonary disease | 1.003 | 0.003 | 1.140 | 0.255 | 0.998 | 1.009 |
| End stage renal disease | 0.727 | 0.004 | -61.790 | 0.000 | 0.720 | 0.735 |
| Midwest vs. Northeast | 0.845 | 0.018 | -8.050 | 0.000 | 0.811 | 0.880 |
| South vs. Northeast | 1.024 | 0.022 | 1.110 | 0.269 | 0.982 | 1.067 |
| West vs. Northeast | 0.785 | 0.022 | -8.470 | 0.000 | 0.742 | 0.830 |
| Hospital referral region (suppressed) |  |  |  |  |  |  |
| Constant | 4.055 | 0.115 | 49.500 | 0.000 | 3.836 | 4.286 |
| **Practice Location** | **Margin** | **SE** | **Z Score** | **P-value** | **LL CI** | **UL CI** |
| Isolated Rural | 0.763 | 0.001 | 863.090 | 0.000 | 0.761 | 0.764 |
| Small Town | 0.765 | 0.001 | 1294.890 | 0.000 | 0.763 | 0.766 |
| Micropolitan | 0.769 | 0.000 | 1796.390 | 0.000 | 0.768 | 0.769 |
| Metropolitan | 0.768 | 0.000 | 4473.990 | 0.000 | 0.768 | 0.768 |
| **Practice Location** | **Contrast** | **SE** | **Z Score** | **P-value** |  |  |
| Small Town vs. Isolated Rural | 0.013 | 0.006 | 1.980 | 0.047 |  |  |
| Micropolitan vs. Isolated Rural | 0.038 | 0.006 | 6.250 | 0.000 |  |  |
| Metropolitan vs. Isolated Rural | 0.033 | 0.006 | 5.850 | 0.000 |  |  |
| Micropolitan vs. Small Town | 0.025 | 0.004 | 5.600 | 0.000 |  |  |
| Metropolitan vs. Small Town | 0.020 | 0.004 | 5.190 | 0.000 |  |  |
| Metropolitan vs. Micropolitan | -0.004 | 0.003 | -1.430 | 0.152 |  |  |

| **Diabetics who had an eye exam** |  |  |  |  |  |  |
| --- | --- | --- | --- | --- | --- | --- |
| N | 7,523,294 |  |  |  |  |  |
| R^2^ | 0.050 |  |  |  |  |  |
|  | **OR** | **SE** | **Z Score** | **P-value** | **LL CI** | **UL CI** |
| Small Town vs. Isolated | 1.067 | 0.006 | 11.140 | 0.000 | 1.055 | 1.079 |
| Micropolitan vs. Isolated | 1.148 | 0.006 | 25.410 | 0.000 | 1.136 | 1.161 |
| Metropolitan vs. Isolated | 1.219 | 0.006 | 38.410 | 0.000 | 1.207 | 1.231 |
| Frail | 1.013 | 0.004 | 3.720 | 0.000 | 1.006 | 1.020 |
| Mean age | 1.027 | 0.000 | 188.540 | 0.000 | 1.027 | 1.027 |
| Under 65 | 0.906 | 0.003 | -25.900 | 0.000 | 0.899 | 0.913 |
| Over 85 | 0.702 | 0.003 | -95.770 | 0.000 | 0.697 | 0.707 |
| Female | 1.300 | 0.002 | 158.360 | 0.000 | 1.295 | 1.304 |
| White vs. Unknown | 0.960 | 0.007 | -5.340 | 0.000 | 0.946 | 0.974 |
| Black vs. Unknown | 0.874 | 0.007 | -16.850 | 0.000 | 0.861 | 0.888 |
| Other vs. Unknown | 0.997 | 0.010 | -0.360 | 0.718 | 0.978 | 1.015 |
| Asian vs. Unknown | 0.917 | 0.009 | -9.250 | 0.000 | 0.900 | 0.934 |
| Hispanic vs. Unknown | 0.870 | 0.008 | -15.370 | 0.000 | 0.854 | 0.885 |
| North American Native vs. Unknown | 1.214 | 0.014 | 16.470 | 0.000 | 1.187 | 1.243 |
| Disabled (original reason for Medicare eligibility) | 0.833 | 0.002 | -71.890 | 0.000 | 0.829 | 0.837 |
| Dual eligible for Medicaid | 0.926 | 0.002 | -35.530 | 0.000 | 0.922 | 0.930 |
| Nursing home | 0.739 | 0.002 | -105.010 | 0.000 | 0.735 | 0.743 |
| Died in the year | 0.508 | 0.002 | -187.340 | 0.000 | 0.504 | 0.512 |
| Median household income (area) | 1.000 | 0.000 | 47.510 | 0.000 | 1.000 | 1.000 |
| Residents under poverty level | 0.999 | 0.000 | -11.540 | 0.000 | 0.998 | 0.999 |
| Number of hierarchical condition categories | 1.022 | 0.001 | 40.920 | 0.000 | 1.021 | 1.024 |
| Coronary artery disease | 0.998 | 0.003 | -0.710 | 0.475 | 0.992 | 1.004 |
| Congestive heart failure | 0.900 | 0.002 | -39.420 | 0.000 | 0.895 | 0.904 |
| Diabetes | 2.152 | 0.006 | 297.260 | 0.000 | 2.141 | 2.163 |
| Cancer | 1.084 | 0.003 | 28.200 | 0.000 | 1.078 | 1.090 |
| Chronic obstructive pulmonary disease | 0.863 | 0.002 | -57.310 | 0.000 | 0.858 | 0.867 |
| End stage renal disease | 1.101 | 0.005 | 19.430 | 0.000 | 1.091 | 1.112 |
| Midwest vs. Northeast | 0.796 | 0.015 | -12.370 | 0.000 | 0.767 | 0.825 |
| South vs. Northeast | 0.776 | 0.015 | -13.540 | 0.000 | 0.748 | 0.805 |
| West vs. Northeast | 0.771 | 0.020 | -10.160 | 0.000 | 0.734 | 0.811 |
| Hospital referral region (suppressed) |  |  |  |  |  |  |
| Constant | 0.136 | 0.003 | -80.780 | 0.000 | 0.130 | 0.143 |
| **Practice Location** | **Margin** | **SE** | **Z Score** | **P-value** | **LL CI** | **UL CI** |
| Isolated Rural | 0.649 | 0.001 | 608.530 | 0.000 | 0.647 | 0.651 |
| Small Town | 0.663 | 0.001 | 953.860 | 0.000 | 0.662 | 0.664 |
| Micropolitan | 0.678 | 0.000 | 1367.310 | 0.000 | 0.677 | 0.679 |
| Metropolitan | 0.690 | 0.000 | 3599.200 | 0.000 | 0.690 | 0.691 |
| **Practice Location** | **Contrast** | **SE** | **Z Score** | **P-value** |  |  |
| Small Town vs. Isolated Rural | 0.065 | 0.006 | 11.140 | 0.000 |  |  |
| Micropolitan vs. Isolated Rural | 0.138 | 0.005 | 25.410 | 0.000 |  |  |
| Metropolitan vs. Isolated Rural | 0.198 | 0.005 | 38.410 | 0.000 |  |  |
| Micropolitan vs. Small Town | 0.073 | 0.004 | 18.520 | 0.000 |  |  |
| Metropolitan vs. Small Town | 0.133 | 0.004 | 37.670 | 0.000 |  |  |
| Metropolitan vs. Micropolitan | 0.060 | 0.003 | 22.160 | 0.000 |  |  |

| **Diabetics who had a hemoglobin A1c test** |  |  |  |  |  |  |
| --- | --- | --- | --- | --- | --- | --- |
| N | 7,523,294 |  |  |  |  |  |
| R^2^ | 0.151 |  |  |  |  |  |
|  | **OR** | **SE** | **Z Score** | **P-value** | **LL CI** | **UL CI** |
| Small Town vs. Isolated | 1.028 | 0.009 | 3.330 | 0.001 | 1.011 | 1.045 |
| Micropolitan vs. Isolated | 1.009 | 0.008 | 1.140 | 0.252 | 0.994 | 1.024 |
| Metropolitan vs. Isolated | 0.992 | 0.007 | -1.080 | 0.282 | 0.978 | 1.006 |
| Frail | 1.062 | 0.005 | 13.520 | 0.000 | 1.053 | 1.072 |
| Mean age | 0.996 | 0.000 | -22.170 | 0.000 | 0.995 | 0.996 |
| Under 65 | 0.774 | 0.004 | -47.650 | 0.000 | 0.766 | 0.782 |
| Over 85 | 0.661 | 0.003 | -88.750 | 0.000 | 0.655 | 0.667 |
| Female | 1.177 | 0.003 | 69.660 | 0.000 | 1.171 | 1.182 |
| White vs. Unknown | 0.904 | 0.011 | -8.310 | 0.000 | 0.883 | 0.926 |
| Black vs. Unknown | 0.764 | 0.010 | -21.590 | 0.000 | 0.746 | 0.783 |
| Other vs. Unknown | 1.047 | 0.016 | 3.040 | 0.002 | 1.016 | 1.078 |
| Asian vs. Unknown | 0.993 | 0.014 | -0.470 | 0.639 | 0.965 | 1.022 |
| Hispanic vs. Unknown | 0.908 | 0.013 | -6.910 | 0.000 | 0.883 | 0.933 |
| North American Native vs. Unknown | 0.165 | 0.002 | -119.650 | 0.000 | 0.160 | 0.170 |
| Disabled (original reason for Medicare eligibility) | 0.825 | 0.003 | -53.990 | 0.000 | 0.820 | 0.831 |
| Dual eligible for Medicaid | 1.061 | 0.003 | 19.710 | 0.000 | 1.055 | 1.067 |
| Nursing home | 0.553 | 0.002 | -168.250 | 0.000 | 0.549 | 0.556 |
| Died in the year | 0.275 | 0.001 | -329.430 | 0.000 | 0.273 | 0.277 |
| Median household income (area) | 1.000 | 0.000 | 12.740 | 0.000 | 1.000 | 1.000 |
| Residents under poverty level | 0.997 | 0.000 | -18.370 | 0.000 | 0.997 | 0.997 |
| Number of hierarchical condition categories | 0.925 | 0.001 | -115.860 | 0.000 | 0.924 | 0.926 |
| Coronary artery disease | 1.039 | 0.004 | 9.360 | 0.000 | 1.031 | 1.048 |
| Congestive heart failure | 0.944 | 0.003 | -16.570 | 0.000 | 0.937 | 0.950 |
| Diabetes | 7.593 | 0.022 | 685.350 | 0.000 | 7.549 | 7.637 |
| Cancer | 0.905 | 0.003 | -27.150 | 0.000 | 0.898 | 0.911 |
| Chronic obstructive pulmonary disease | 0.876 | 0.003 | -39.370 | 0.000 | 0.870 | 0.882 |
| End stage renal disease | 1.299 | 0.008 | 40.580 | 0.000 | 1.283 | 1.316 |
| Midwest vs. Northeast | 0.851 | 0.022 | -6.360 | 0.000 | 0.810 | 0.894 |
| South vs. Northeast | 0.869 | 0.022 | -5.500 | 0.000 | 0.826 | 0.913 |
| West vs. Northeast | 0.888 | 0.031 | -3.350 | 0.001 | 0.829 | 0.952 |
| Hospital referral region (suppressed) |  |  |  |  |  |  |
| Constant | 3.279 | 0.114 | 34.270 | 0.000 | 3.064 | 3.509 |
| **Practice Location** | **Margin** | **SE** | **Z Score** | **P-value** | **LL CI** | **UL CI** |
| Isolated Rural | 0.860 | 0.001 | 1190.860 | 0.000 | 0.859 | 0.862 |
| Small Town | 0.863 | 0.000 | 1802.870 | 0.000 | 0.862 | 0.864 |
| Micropolitan | 0.861 | 0.000 | 2448.880 | 0.000 | 0.861 | 0.862 |
| Metropolitan | 0.860 | 0.000 | 6304.220 | 0.000 | 0.859 | 0.860 |
| **Practice Location** | **Contrast** | **SE** | **Z Score** | **P-value** |  |  |
| Small Town vs. Isolated Rural | 0.028 | 0.008 | 3.330 | 0.001 |  |  |
| Micropolitan vs. Isolated Rural | 0.009 | 0.008 | 1.140 | 0.252 |  |  |
| Metropolitan vs. Isolated Rural | -0.008 | 0.007 | -1.080 | 0.282 |  |  |
| Micropolitan vs. Small Town | -0.019 | 0.006 | -3.300 | 0.001 |  |  |
| Metropolitan vs. Small Town | -0.036 | 0.005 | -7.020 | 0.000 |  |  |
| Metropolitan vs. Micropolitan | -0.017 | 0.004 | -4.350 | 0.000 |  |  |

| **Mammogram, aged 50-74** |  |  |  |  |  |  |
| --- | --- | --- | --- | --- | --- | --- |
| N | 7,361,125 |  |  |  |  |  |
| R^2^ | 0.047 |  |  |  |  |  |
|  | **OR** | **SE** | **Z Score** | **P-value** | **LL CI** | **UL CI** |
| Small Town vs. Isolated | 1.101 | 0.006 | 16.420 | 0.000 | 1.088 | 1.113 |
| Micropolitan vs. Isolated | 1.240 | 0.007 | 39.800 | 0.000 | 1.227 | 1.254 |
| Metropolitan vs. Isolated | 1.285 | 0.007 | 49.080 | 0.000 | 1.272 | 1.298 |
| Frail | 0.956 | 0.005 | -9.400 | 0.000 | 0.947 | 0.965 |
| Mean age | 0.988 | 0.000 | -43.070 | 0.000 | 0.987 | 0.988 |
| Under 65 | 0.770 | 0.004 | -56.570 | 0.000 | 0.763 | 0.777 |
| White vs. Unknown | 0.861 | 0.006 | -20.830 | 0.000 | 0.849 | 0.874 |
| Black vs. Unknown | 1.102 | 0.008 | 12.620 | 0.000 | 1.085 | 1.118 |
| Other vs. Unknown | 0.793 | 0.008 | -23.430 | 0.000 | 0.778 | 0.809 |
| Asian vs. Unknown | 0.674 | 0.006 | -42.390 | 0.000 | 0.661 | 0.686 |
| Hispanic vs. Unknown | 0.958 | 0.009 | -4.480 | 0.000 | 0.941 | 0.976 |
| North American Native vs. Unknown | 0.603 | 0.007 | -41.580 | 0.000 | 0.589 | 0.618 |
| Disabled (original reason for Medicare eligibility) | 0.703 | 0.002 | -127.850 | 0.000 | 0.699 | 0.706 |
| Dual eligible for Medicaid | 0.668 | 0.002 | -173.300 | 0.000 | 0.665 | 0.671 |
| Nursing home | 0.481 | 0.002 | -175.420 | 0.000 | 0.477 | 0.485 |
| Died in the year | 0.328 | 0.002 | -162.850 | 0.000 | 0.323 | 0.332 |
| Median household income (area) | 1.000 | 0.000 | 53.980 | 0.000 | 1.000 | 1.000 |
| Residents under poverty level | 0.998 | 0.000 | -13.870 | 0.000 | 0.998 | 0.999 |
| Number of hierarchical condition categories | 0.979 | 0.001 | -28.500 | 0.000 | 0.978 | 0.981 |
| Coronary artery disease | 0.987 | 0.005 | -2.660 | 0.008 | 0.978 | 0.997 |
| Congestive heart failure | 0.816 | 0.003 | -52.260 | 0.000 | 0.810 | 0.823 |
| Diabetes | 1.063 | 0.002 | 28.110 | 0.000 | 1.058 | 1.067 |
| Cancer | 1.493 | 0.005 | 122.510 | 0.000 | 1.484 | 1.503 |
| Chronic obstructive pulmonary disease | 0.794 | 0.002 | -77.120 | 0.000 | 0.789 | 0.799 |
| End stage renal disease | 0.866 | 0.007 | -17.430 | 0.000 | 0.852 | 0.880 |
| Midwest vs. Northeast | 0.870 | 0.017 | -7.210 | 0.000 | 0.838 | 0.904 |
| South vs. Northeast | 0.926 | 0.018 | -3.990 | 0.000 | 0.891 | 0.961 |
| West vs. Northeast | 1.110 | 0.029 | 3.960 | 0.000 | 1.054 | 1.168 |
| Hospital referral region (suppressed) |  |  |  |  |  |  |
| Constant | 4.314 | 0.130 | 48.490 | 0.000 | 4.067 | 4.577 |
| **Practice Location** | **Margin** | **SE** | **Z Score** | **P-value** | **LL CI** | **UL CI** |
| Isolated Rural | 0.598 | 0.001 | 532.720 | 0.000 | 0.596 | 0.601 |
| Small Town | 0.620 | 0.001 | 831.010 | 0.000 | 0.618 | 0.621 |
| Micropolitan | 0.646 | 0.001 | 1237.340 | 0.000 | 0.645 | 0.647 |
| Metropolitan | 0.653 | 0.000 | 3297.970 | 0.000 | 0.653 | 0.654 |
| **Practice Location** | **Contrast** | **SE** | **Z Score** | **P-value** |  |  |
| Small Town vs. Isolated Rural | 0.096 | 0.006 | 16.420 | 0.000 |  |  |
| Micropolitan vs. Isolated Rural | 0.215 | 0.005 | 39.800 | 0.000 |  |  |
| Metropolitan vs. Isolated Rural | 0.251 | 0.005 | 49.080 | 0.000 |  |  |
| Micropolitan vs. Small Town | 0.120 | 0.004 | 29.930 | 0.000 |  |  |
| Metropolitan vs. Small Town | 0.155 | 0.004 | 43.500 | 0.000 |  |  |
| Metropolitan vs. Micropolitan | 0.035 | 0.003 | 13.140 | 0.000 |  |  |

| **Primary care clinician visit within 14-days of stay** |  |  |  |  |  |  |
| --- | --- | --- | --- | --- | --- | --- |
| N | 2,268,980 |  |  |  |  |  |
| R^2^ | 0.028 |  |  |  |  |  |
|  | **OR** | **SE** | **Z Score** | **P-value** | **LL CI** | **UL CI** |
| Small Town vs. Isolated | 0.921 | 0.010 | -7.610 | 0.000 | 0.902 | 0.941 |
| Micropolitan vs. Isolated | 0.796 | 0.008 | -22.810 | 0.000 | 0.781 | 0.812 |
| Metropolitan vs. Isolated | 0.671 | 0.006 | -42.060 | 0.000 | 0.659 | 0.684 |
| Frail | 0.978 | 0.005 | -4.550 | 0.000 | 0.969 | 0.987 |
| Mean age | 1.005 | 0.000 | 24.890 | 0.000 | 1.005 | 1.005 |
| Under 65 | 0.924 | 0.006 | -11.940 | 0.000 | 0.912 | 0.936 |
| Over 85 | 0.757 | 0.004 | -54.470 | 0.000 | 0.749 | 0.764 |
| Female | 1.019 | 0.003 | 6.660 | 0.000 | 1.013 | 1.024 |
| White vs. Unknown | 1.032 | 0.015 | 2.130 | 0.033 | 1.003 | 1.063 |
| Black vs. Unknown | 0.825 | 0.013 | -12.400 | 0.000 | 0.801 | 0.851 |
| Other vs. Unknown | 1.001 | 0.020 | 0.070 | 0.948 | 0.964 | 1.040 |
| Asian vs. Unknown | 1.026 | 0.019 | 1.350 | 0.177 | 0.989 | 1.064 |
| Hispanic vs. Unknown | 0.989 | 0.017 | -0.620 | 0.537 | 0.956 | 1.024 |
| North American Native vs. Unknown | 0.883 | 0.019 | -5.720 | 0.000 | 0.846 | 0.922 |
| Disabled (original reason for Medicare eligibility) | 0.903 | 0.004 | -23.020 | 0.000 | 0.895 | 0.911 |
| Dual eligible for Medicaid | 0.836 | 0.003 | -48.990 | 0.000 | 0.830 | 0.842 |
| Nursing home | 0.623 | 0.003 | -111.300 | 0.000 | 0.618 | 0.628 |
| Died in the year | 0.805 | 0.004 | -46.950 | 0.000 | 0.798 | 0.812 |
| Median household income (area) | 1.000 | 0.000 | 5.000 | 0.000 | 1.000 | 1.000 |
| Residents under poverty level | 0.998 | 0.000 | -11.210 | 0.000 | 0.997 | 0.998 |
| Number of hierarchical condition categories | 1.030 | 0.001 | 40.350 | 0.000 | 1.029 | 1.032 |
| Coronary artery disease | 1.033 | 0.004 | 7.630 | 0.000 | 1.025 | 1.042 |
| Congestive heart failure | 1.091 | 0.004 | 24.430 | 0.000 | 1.083 | 1.098 |
| Diabetes | 1.075 | 0.003 | 23.220 | 0.000 | 1.068 | 1.082 |
| Cancer | 1.414 | 0.006 | 85.540 | 0.000 | 1.403 | 1.425 |
| Chronic obstructive pulmonary disease | 1.014 | 0.003 | 4.030 | 0.000 | 1.007 | 1.020 |
| End stage renal disease | 0.641 | 0.004 | -65.280 | 0.000 | 0.632 | 0.650 |
| Midwest vs. Northeast | 1.257 | 0.038 | 7.670 | 0.000 | 1.186 | 1.333 |
| South vs. Northeast | 0.987 | 0.030 | -0.420 | 0.678 | 0.930 | 1.048 |
| West vs. Northeast | 1.168 | 0.052 | 3.480 | 0.000 | 1.070 | 1.275 |
| Hospital referral region (suppressed) |  |  |  |  |  |  |
| Constant | 1.348 | 0.055 | 7.270 | 0.000 | 1.244 | 1.461 |
| **Practice Location** | **Margin** | **SE** | **Z Score** | **P-value** | **LL CI** | **UL CI** |
| Isolated Rural | 0.671 | 0.002 | 339.940 | 0.000 | 0.667 | 0.675 |
| Small Town | 0.653 | 0.001 | 487.190 | 0.000 | 0.651 | 0.656 |
| Micropolitan | 0.621 | 0.001 | 631.130 | 0.000 | 0.619 | 0.623 |
| Metropolitan | 0.581 | 0.000 | 1556.610 | 0.000 | 0.581 | 0.582 |
| **Practice Location** | **Contrast** | **SE** | **Z Score** | **P-value** |  |  |
| Small Town vs. Isolated Rural | -0.082 | 0.011 | -7.610 | 0.000 |  |  |
| Micropolitan vs. Isolated Rural | -0.228 | 0.010 | -22.810 | 0.000 |  |  |
| Metropolitan vs. Isolated Rural | -0.398 | 0.009 | -42.060 | 0.000 |  |  |
| Micropolitan vs. Small Town | -0.146 | 0.007 | -20.180 | 0.000 |  |  |
| Metropolitan vs. Small Town | -0.316 | 0.006 | -49.080 | 0.000 |  |  |
| Metropolitan vs. Micropolitan | -0.170 | 0.005 | -35.850 | 0.000 |  |  |

| **Follow-up within 30-days of mental health stay** |  |  |  |  |  |  |
| --- | --- | --- | --- | --- | --- | --- |
| N | 178,498 |  |  |  |  |  |
| R^2^ | 0.103 |  |  |  |  |  |
|  | **OR** | **SE** | **Z Score** | **P-value** | **LL CI** | **UL CI** |
| Small Town vs. Isolated | 0.009 | 0.008 | 1.150 | 0.251 | -0.007 | 0.025 |
| Micropolitan vs. Isolated | 0.003 | 0.008 | 0.420 | 0.676 | -0.012 | 0.018 |
| Metropolitan vs. Isolated | -0.023 | 0.007 | -3.150 | 0.002 | -0.037 | -0.009 |
| Frail | -0.028 | 0.004 | -6.280 | 0.000 | -0.037 | -0.019 |
| Mean age | 0.001 | 0.000 | 7.100 | 0.000 | 0.001 | 0.001 |
| Under 65 | 0.019 | 0.004 | 4.340 | 0.000 | 0.010 | 0.027 |
| Over 85 | -0.078 | 0.007 | -11.660 | 0.000 | -0.091 | -0.065 |
| Female | 0.071 | 0.002 | 34.060 | 0.000 | 0.067 | 0.075 |
| White vs. Unknown | 0.001 | 0.009 | 0.060 | 0.950 | -0.018 | 0.019 |
| Black vs. Unknown | -0.082 | 0.010 | -8.510 | 0.000 | -0.100 | -0.063 |
| Other vs. Unknown | 0.008 | 0.014 | 0.540 | 0.586 | -0.020 | 0.036 |
| Asian vs. Unknown | -0.022 | 0.013 | -1.690 | 0.091 | -0.048 | 0.004 |
| Hispanic vs. Unknown | -0.016 | 0.011 | -1.440 | 0.151 | -0.037 | 0.006 |
| North American Native vs. Unknown | -0.058 | 0.014 | -4.040 | 0.000 | -0.086 | -0.030 |
| Disabled (original reason for Medicare eligibility) | -0.017 | 0.004 | -4.310 | 0.000 | -0.025 | -0.009 |
| Dual eligible for Medicaid | -0.066 | 0.003 | -25.510 | 0.000 | -0.071 | -0.061 |
| Nursing home | -0.260 | 0.003 | -89.620 | 0.000 | -0.266 | -0.255 |
| Died in the year | -0.171 | 0.005 | -32.180 | 0.000 | -0.182 | -0.161 |
| Median household income (area) | 0.000 | 0.000 | 1.670 | 0.095 | 0.000 | 0.000 |
| Residents under poverty level | -0.001 | 0.000 | -6.450 | 0.000 | -0.001 | -0.001 |
| Number of hierarchical condition categories | 0.009 | 0.001 | 14.530 | 0.000 | 0.008 | 0.010 |
| Coronary artery disease | 0.030 | 0.005 | 6.270 | 0.000 | 0.020 | 0.039 |
| Congestive heart failure | 0.000 | 0.004 | 0.050 | 0.962 | -0.007 | 0.008 |
| Diabetes | 0.032 | 0.003 | 12.520 | 0.000 | 0.027 | 0.036 |
| Cancer | 0.064 | 0.005 | 13.860 | 0.000 | 0.055 | 0.074 |
| Chronic obstructive pulmonary disease | 0.007 | 0.003 | 2.530 | 0.011 | 0.002 | 0.013 |
| End stage renal disease | -0.024 | 0.012 | -2.060 | 0.040 | -0.046 | -0.001 |
| Midwest vs. Northeast | -0.028 | 0.024 | -1.150 | 0.248 | -0.075 | 0.019 |
| South vs. Northeast | -0.018 | 0.025 | -0.750 | 0.453 | -0.067 | 0.030 |
| West vs. Northeast | -0.053 | 0.036 | -1.450 | 0.148 | -0.124 | 0.019 |
| Hospital referral region (suppressed) |  |  |  |  |  |  |
| Constant | 0.674 | 0.030 | 22.290 | 0.000 | 0.614 | 0.733 |
| **Practice Location** | **Margin** | **SE** | **Z Score** | **P-value** | **LL CI** | **UL CI** |
| Isolated Rural | 0.702 | 0.007 | 100.120 | 0.000 | 0.688 | 0.716 |
| Small Town | 0.711 | 0.004 | 158.700 | 0.000 | 0.702 | 0.720 |
| Micropolitan | 0.705 | 0.003 | 223.270 | 0.000 | 0.699 | 0.711 |
| Metropolitan | 0.679 | 0.001 | 579.290 | 0.000 | 0.677 | 0.682 |
| **Practice Location** | **Contrast** | **SE** | **Z Score** | **P-value** |  |  |
| Small Town vs. Isolated Rural | 0.009 | 0.008 | 1.150 | 0.251 |  |  |
| Micropolitan vs. Isolated Rural | 0.003 | 0.008 | 0.420 | 0.676 |  |  |
| Metropolitan vs. Isolated Rural | -0.023 | 0.007 | -3.150 | 0.002 |  |  |
| Micropolitan vs. Small Town | -0.006 | 0.005 | -1.170 | 0.242 |  |  |
| Metropolitan vs. Small Town | -0.032 | 0.005 | -6.770 | 0.000 |  |  |
| Metropolitan vs. Micropolitan | -0.026 | 0.003 | -7.420 | 0.000 |  |  |

| **Follow-up within 7-days of mental health stay** |  |  |  |  |  |  |
| --- | --- | --- | --- | --- | --- | --- |
| N | 178,498 |  |  |  |  |  |
| R^2^ | 0.066 |  |  |  |  |  |
|  | **OR** | **SE** | **Z Score** | **P-value** | **LL CI** | **UL CI** |
| Small Town vs. Isolated | 0.006 | 0.009 | 0.700 | 0.481 | -0.011 | 0.023 |
| Micropolitan vs. Isolated | -0.006 | 0.008 | -0.760 | 0.448 | -0.022 | 0.010 |
| Metropolitan vs. Isolated | -0.010 | 0.008 | -1.240 | 0.214 | -0.025 | 0.005 |
| Frail | -0.020 | 0.005 | -4.190 | 0.000 | -0.029 | -0.011 |
| Mean age | 0.001 | 0.000 | 5.090 | 0.000 | 0.000 | 0.001 |
| Under 65 | 0.011 | 0.005 | 2.400 | 0.016 | 0.002 | 0.020 |
| Over 85 | -0.097 | 0.007 | -13.610 | 0.000 | -0.111 | -0.083 |
| Female | 0.054 | 0.002 | 24.370 | 0.000 | 0.050 | 0.058 |
| White vs. Unknown | -0.011 | 0.010 | -1.090 | 0.277 | -0.030 | 0.009 |
| Black vs. Unknown | -0.085 | 0.010 | -8.310 | 0.000 | -0.105 | -0.065 |
| Other vs. Unknown | -0.018 | 0.015 | -1.140 | 0.255 | -0.048 | 0.013 |
| Asian vs. Unknown | -0.036 | 0.014 | -2.540 | 0.011 | -0.063 | -0.008 |
| Hispanic vs. Unknown | -0.036 | 0.012 | -3.080 | 0.002 | -0.059 | -0.013 |
| North American Native vs. Unknown | -0.052 | 0.015 | -3.390 | 0.001 | -0.082 | -0.022 |
| Disabled (original reason for Medicare eligibility) | -0.015 | 0.004 | -3.430 | 0.001 | -0.023 | -0.006 |
| Dual eligible for Medicaid | -0.066 | 0.003 | -23.900 | 0.000 | -0.072 | -0.061 |
| Nursing home | -0.200 | 0.003 | -64.220 | 0.000 | -0.206 | -0.194 |
| Died in the year | -0.098 | 0.006 | -17.210 | 0.000 | -0.109 | -0.087 |
| Median household income (area) | 0.000 | 0.000 | 4.220 | 0.000 | 0.000 | 0.000 |
| Residents under poverty level | -0.001 | 0.000 | -5.000 | 0.000 | -0.001 | 0.000 |
| Number of hierarchical condition categories | 0.006 | 0.001 | 10.010 | 0.000 | 0.005 | 0.008 |
| Coronary artery disease | 0.020 | 0.005 | 3.960 | 0.000 | 0.010 | 0.030 |
| Congestive heart failure | -0.009 | 0.004 | -2.200 | 0.028 | -0.017 | -0.001 |
| Diabetes | 0.026 | 0.003 | 9.720 | 0.000 | 0.021 | 0.032 |
| Cancer | 0.045 | 0.005 | 9.100 | 0.000 | 0.036 | 0.055 |
| Chronic obstructive pulmonary disease | 0.000 | 0.003 | 0.020 | 0.983 | -0.006 | 0.006 |
| End stage renal disease | -0.076 | 0.012 | -6.130 | 0.000 | -0.100 | -0.051 |
| Midwest vs. Northeast | 0.014 | 0.026 | 0.540 | 0.586 | -0.036 | 0.065 |
| South vs. Northeast | -0.009 | 0.026 | -0.360 | 0.721 | -0.061 | 0.042 |
| West vs. Northeast | -0.039 | 0.039 | -1.000 | 0.318 | -0.115 | 0.037 |
| Hospital referral region (suppressed) |  |  |  |  |  |  |
| Constant | 0.361 | 0.032 | 11.180 | 0.000 | 0.298 | 0.425 |
| **Practice Location** | **Margin** | **SE** | **Z Score** | **P-value** | **LL CI** | **UL CI** |
| Isolated Rural | 0.397 | 0.008 | 52.930 | 0.000 | 0.382 | 0.412 |
| Small Town | 0.403 | 0.005 | 84.070 | 0.000 | 0.394 | 0.413 |
| Micropolitan | 0.391 | 0.003 | 115.680 | 0.000 | 0.384 | 0.398 |
| Metropolitan | 0.388 | 0.001 | 308.800 | 0.000 | 0.385 | 0.390 |
| **Practice Location** | **Contrast** | **SE** | **Z Score** | **P-value** |  |  |
| Small Town vs. Isolated Rural | 0.006 | 0.009 | 0.700 | 0.481 |  |  |
| Micropolitan vs. Isolated Rural | -0.006 | 0.008 | -0.760 | 0.448 |  |  |
| Metropolitan vs. Isolated Rural | -0.010 | 0.008 | -1.240 | 0.214 |  |  |
| Micropolitan vs. Small Town | -0.012 | 0.006 | -2.170 | 0.030 |  |  |
| Metropolitan vs. Small Town | -0.016 | 0.005 | -3.110 | 0.002 |  |  |
| Metropolitan vs. Micropolitan | -0.003 | 0.004 | -0.920 | 0.359 |  |  |

| **Number of outpatient visits** |  |  |  |  |  |  |
| --- | --- | --- | --- | --- | --- | --- |
| N | 27,710,872 |  |  |  |  |  |
| R^2^ | 0.320 |  |  |  |  |  |
|  | **Coefficient** | **SE** | **t** | **P-value** | **LL CI** | **UL CI** |
| Small Town vs. Isolated | -0.200 | 0.012 | -17.260 | 0.000 | -0.223 | -0.177 |
| Micropolitan vs. Isolated | 0.138 | 0.011 | 12.910 | 0.000 | 0.117 | 0.159 |
| Metropolitan vs. Isolated | 0.312 | 0.010 | 30.840 | 0.000 | 0.292 | 0.332 |
| Frail | 4.790 | 0.008 | 612.530 | 0.000 | 4.775 | 4.806 |
| Mean age | 0.063 | 0.000 | 251.250 | 0.000 | 0.063 | 0.064 |
| Under 65 | 0.939 | 0.008 | 110.650 | 0.000 | 0.923 | 0.956 |
| Over 85 | -1.181 | 0.007 | -175.600 | 0.000 | -1.195 | -1.168 |
| Female | 1.001 | 0.003 | 314.040 | 0.000 | 0.995 | 1.008 |
| White vs. Unknown | 0.386 | 0.013 | 29.850 | 0.000 | 0.360 | 0.411 |
| Black vs. Unknown | -0.646 | 0.014 | -46.010 | 0.000 | -0.674 | -0.619 |
| Other vs. Unknown | -0.827 | 0.018 | -45.280 | 0.000 | -0.863 | -0.791 |
| Asian vs. Unknown | -0.922 | 0.018 | -51.700 | 0.000 | -0.957 | -0.887 |
| Hispanic vs. Unknown | -0.381 | 0.018 | -21.550 | 0.000 | -0.416 | -0.347 |
| North American Native vs. Unknown | 1.935 | 0.025 | 78.620 | 0.000 | 1.887 | 1.983 |
| Disabled (original reason for Medicare eligibility) | 1.189 | 0.006 | 200.070 | 0.000 | 1.177 | 1.201 |
| Dual eligible for Medicaid | 0.733 | 0.005 | 154.220 | 0.000 | 0.724 | 0.743 |
| Nursing home | 2.859 | 0.007 | 436.140 | 0.000 | 2.846 | 2.871 |
| Died in the year | -9.586 | 0.009 | -1099.800 | 0.000 | -9.603 | -9.569 |
| Median household income (area) | 0.000 | 0.000 | 68.960 | 0.000 | 0.000 | 0.000 |
| Residents under poverty level | 0.004 | 0.000 | 17.020 | 0.000 | 0.003 | 0.004 |
| Number of hierarchical condition categories | 2.283 | 0.001 | 1770.730 | 0.000 | 2.280 | 2.285 |
| Coronary artery disease | -0.741 | 0.008 | -95.470 | 0.000 | -0.757 | -0.726 |
| Congestive heart failure | -0.606 | 0.006 | -95.140 | 0.000 | -0.618 | -0.593 |
| Diabetes | -0.099 | 0.004 | -24.210 | 0.000 | -0.107 | -0.091 |
| Cancer | 3.333 | 0.006 | 591.290 | 0.000 | 3.322 | 3.344 |
| Chronic obstructive pulmonary disease | -0.134 | 0.006 | -23.350 | 0.000 | -0.145 | -0.123 |
| End stage renal disease | -5.814 | 0.015 | -391.430 | 0.000 | -5.843 | -5.785 |
| Midwest vs. Northeast | 0.528 | 0.038 | 13.940 | 0.000 | 0.453 | 0.602 |
| South vs. Northeast | 0.844 | 0.038 | 22.060 | 0.000 | 0.769 | 0.919 |
| West vs. Northeast | 1.977 | 0.052 | 38.010 | 0.000 | 1.875 | 2.079 |
| Hospital referral region (suppressed) |  |  |  |  |  |  |
| Constant | -1.000 | 0.049 | -20.610 | 0.000 | -1.096 | -0.905 |
| **Practice Location** | **Margin** | **SE** | **t** | **P-value** | **LL CI** | **UL CI** |
| Isolated Rural | 10.987 | 0.010 | 1114.270 | 0.000 | 10.968 | 11.007 |
| Small Town | 10.787 | 0.007 | 1609.120 | 0.000 | 10.774 | 10.800 |
| Micropolitan | 11.126 | 0.005 | 2311.160 | 0.000 | 11.116 | 11.135 |
| Metropolitan | 11.299 | 0.002 | 6230.340 | 0.000 | 11.296 | 11.303 |
| **Practice Location** | **Contrast** | **SE** | **t** | **P-value** |  |  |
| Small town vs. Isolated Rural | -0.200 | 0.012 | -17.260 | 0.000 |  |  |
| Micropolitan vs. Isolated Rural | 0.138 | 0.011 | 12.910 | 0.000 |  |  |
| Metropolitan vs. Isolated rural | 0.312 | 0.010 | 30.840 | 0.000 |  |  |
| Micropolitan vs. Small Town | 0.338 | 0.008 | 42.610 | 0.000 |  |  |
| Metropolitan vs. Small Town | 0.512 | 0.007 | 72.430 | 0.000 |  |  |
| Metropolitan vs. Micropolitan | 0.174 | 0.005 | 32.780 | 0.000 |  |  |

| **Number of outpatient visits, family medicine** |  |  |  |  |  |  |
| --- | --- | --- | --- | --- | --- | --- |
| N | 27,710,872 |  |  |  |  |  |
| R^2^ | 0.071 |  |  |  |  |  |
|  | **Coefficient** | **SE** | **t** | **P-value** | **LL CI** | **UL CI** |
| Small Town vs. Isolated | -0.067 | 0.005 | -14.810 | 0.000 | -0.076 | -0.058 |
| Micropolitan vs. Isolated | -0.434 | 0.004 | -103.590 | 0.000 | -0.442 | -0.426 |
| Metropolitan vs. Isolated | -0.773 | 0.004 | -195.420 | 0.000 | -0.780 | -0.765 |
| Frail | 0.509 | 0.003 | 166.690 | 0.000 | 0.503 | 0.515 |
| Mean age | 0.009 | 0.000 | 91.340 | 0.000 | 0.009 | 0.009 |
| Under 65 | 0.086 | 0.003 | 25.850 | 0.000 | 0.079 | 0.092 |
| Over 85 | -0.103 | 0.003 | -39.150 | 0.000 | -0.108 | -0.098 |
| Female | 0.199 | 0.001 | 159.440 | 0.000 | 0.196 | 0.201 |
| White vs. Unknown | 0.072 | 0.005 | 14.250 | 0.000 | 0.062 | 0.082 |
| Black vs. Unknown | -0.164 | 0.005 | -29.970 | 0.000 | -0.175 | -0.154 |
| Other vs. Unknown | -0.099 | 0.007 | -13.920 | 0.000 | -0.113 | -0.085 |
| Asian vs. Unknown | -0.079 | 0.007 | -11.280 | 0.000 | -0.092 | -0.065 |
| Hispanic vs. Unknown | 0.186 | 0.007 | 26.890 | 0.000 | 0.172 | 0.199 |
| North American Native vs. Unknown | 0.749 | 0.010 | 77.940 | 0.000 | 0.731 | 0.768 |
| Disabled (original reason for Medicare eligibility) | 0.173 | 0.002 | 74.680 | 0.000 | 0.169 | 0.178 |
| Dual eligible for Medicaid | 0.298 | 0.002 | 160.670 | 0.000 | 0.295 | 0.302 |
| Nursing home | 0.602 | 0.003 | 235.270 | 0.000 | 0.597 | 0.607 |
| Died in the year | -1.264 | 0.003 | -371.160 | 0.000 | -1.270 | -1.257 |
| Median household income (area) | 0.000 | 0.000 | -36.780 | 0.000 | 0.000 | 0.000 |
| Residents under poverty level | -0.004 | 0.000 | -43.380 | 0.000 | -0.004 | -0.004 |
| Number of hierarchical condition categories | 0.216 | 0.001 | 428.080 | 0.000 | 0.215 | 0.217 |
| Coronary artery disease | -0.133 | 0.003 | -43.680 | 0.000 | -0.138 | -0.127 |
| Congestive heart failure | -0.066 | 0.002 | -26.350 | 0.000 | -0.070 | -0.061 |
| Diabetes | 0.285 | 0.002 | 178.580 | 0.000 | 0.282 | 0.289 |
| Cancer | -0.215 | 0.002 | -97.760 | 0.000 | -0.220 | -0.211 |
| Chronic obstructive pulmonary disease | 0.281 | 0.002 | 125.570 | 0.000 | 0.277 | 0.286 |
| End stage renal disease | -1.079 | 0.006 | -185.970 | 0.000 | -1.090 | -1.068 |
| Midwest vs. Northeast | 0.025 | 0.015 | 1.710 | 0.087 | -0.004 | 0.054 |
| South vs. Northeast | 0.112 | 0.015 | 7.500 | 0.000 | 0.083 | 0.141 |
| West vs. Northeast | 0.170 | 0.020 | 8.380 | 0.000 | 0.130 | 0.210 |
| Hospital referral region (suppressed) |  |  |  |  |  |  |
| Constant | 1.247 | 0.019 | 65.750 | 0.000 | 1.209 | 1.284 |
| **Practice Location** | **Margin** | **SE** | **t** | **P-value** | **LL CI** | **UL CI** |
| Isolated Rural | 2.574 | 0.004 | 668.330 | 0.000 | 2.567 | 2.582 |
| Small Town | 2.507 | 0.003 | 957.420 | 0.000 | 2.502 | 2.512 |
| Micropolitan | 2.140 | 0.002 | 1138.180 | 0.000 | 2.137 | 2.144 |
| Metropolitan | 1.802 | 0.001 | 2543.230 | 0.000 | 1.800 | 1.803 |
| **Practice Location** | **Contrast** | **SE** | **t** | **P-value** |  |  |
| Small town vs. Isolated Rural | -0.067 | 0.005 | -14.810 | 0.000 |  |  |
| Micropolitan vs. Isolated Rural | -0.434 | 0.004 | -103.590 | 0.000 |  |  |
| Metropolitan vs. Isolated rural | -0.773 | 0.004 | -195.420 | 0.000 |  |  |
| Micropolitan vs. Small Town | -0.367 | 0.003 | -118.310 | 0.000 |  |  |
| Metropolitan vs. Small Town | -0.705 | 0.003 | -255.500 | 0.000 |  |  |
| Metropolitan vs. Micropolitan | -0.339 | 0.002 | -163.650 | 0.000 |  |  |

| **Number of outpatient visits, internist** |  |  |  |  |  |  |
| --- | --- | --- | --- | --- | --- | --- |
| N | 27,710,872 |  |  |  |  |  |
| R^2^ | 0.120 |  |  |  |  |  |
|  | **Coefficient** | **SE** | **t** | **P-value** | **LL CI** | **UL CI** |
| Small Town vs. Isolated | 0.180 | 0.005 | 39.300 | 0.000 | 0.171 | 0.189 |
| Micropolitan vs. Isolated | 0.569 | 0.004 | 133.860 | 0.000 | 0.561 | 0.578 |
| Metropolitan vs. Isolated | 0.740 | 0.004 | 184.430 | 0.000 | 0.732 | 0.748 |
| Frail | 0.908 | 0.003 | 292.790 | 0.000 | 0.902 | 0.914 |
| Mean age | 0.029 | 0.000 | 289.930 | 0.000 | 0.029 | 0.029 |
| Under 65 | 0.156 | 0.003 | 46.340 | 0.000 | 0.149 | 0.163 |
| Over 85 | -0.084 | 0.003 | -31.680 | 0.000 | -0.090 | -0.079 |
| Female | 0.158 | 0.001 | 125.310 | 0.000 | 0.156 | 0.161 |
| White vs. Unknown | -0.025 | 0.005 | -4.850 | 0.000 | -0.035 | -0.015 |
| Black vs. Unknown | 0.152 | 0.006 | 27.230 | 0.000 | 0.141 | 0.163 |
| Other vs. Unknown | 0.175 | 0.007 | 24.100 | 0.000 | 0.160 | 0.189 |
| Asian vs. Unknown | 0.640 | 0.007 | 90.480 | 0.000 | 0.626 | 0.653 |
| Hispanic vs. Unknown | -0.104 | 0.007 | -14.890 | 0.000 | -0.118 | -0.091 |
| North American Native vs. Unknown | -0.057 | 0.010 | -5.890 | 0.000 | -0.077 | -0.038 |
| Disabled (original reason for Medicare eligibility) | 0.059 | 0.002 | 24.910 | 0.000 | 0.054 | 0.063 |
| Dual eligible for Medicaid | 0.158 | 0.002 | 84.040 | 0.000 | 0.155 | 0.162 |
| Nursing home | 1.094 | 0.003 | 420.850 | 0.000 | 1.089 | 1.099 |
| Died in the year | -1.643 | 0.003 | -475.490 | 0.000 | -1.650 | -1.636 |
| Median household income (area) | 0.000 | 0.000 | 22.940 | 0.000 | 0.000 | 0.000 |
| Residents under poverty level | 0.004 | 0.000 | 41.550 | 0.000 | 0.004 | 0.004 |
| Number of hierarchical condition categories | 0.319 | 0.001 | 624.460 | 0.000 | 0.318 | 0.320 |
| Coronary artery disease | -0.155 | 0.003 | -50.200 | 0.000 | -0.161 | -0.149 |
| Congestive heart failure | -0.024 | 0.003 | -9.460 | 0.000 | -0.029 | -0.019 |
| Diabetes | 0.175 | 0.002 | 108.140 | 0.000 | 0.172 | 0.179 |
| Cancer | -0.018 | 0.002 | -8.190 | 0.000 | -0.023 | -0.014 |
| Chronic obstructive pulmonary disease | 0.127 | 0.002 | 55.930 | 0.000 | 0.123 | 0.132 |
| End stage renal disease | -0.941 | 0.006 | -159.750 | 0.000 | -0.952 | -0.929 |
| Midwest vs. Northeast | -0.058 | 0.015 | -3.860 | 0.000 | -0.087 | -0.029 |
| South vs. Northeast | 0.034 | 0.015 | 2.220 | 0.026 | 0.004 | 0.063 |
| West vs. Northeast | 0.392 | 0.021 | 19.000 | 0.000 | 0.351 | 0.432 |
| Hospital referral region (suppressed) |  |  |  |  |  |  |
| Constant | -1.761 | 0.019 | -91.510 | 0.000 | -1.799 | -1.723 |
| **Practice Location** | **Margin** | **SE** | **t** | **P-value** | **LL CI** | **UL CI** |
| Isolated Rural | 1.218 | 0.004 | 311.520 | 0.000 | 1.210 | 1.226 |
| Small Town | 1.398 | 0.003 | 526.100 | 0.000 | 1.393 | 1.404 |
| Micropolitan | 1.787 | 0.002 | 936.290 | 0.000 | 1.783 | 1.791 |
| Metropolitan | 1.958 | 0.001 | 2722.830 | 0.000 | 1.957 | 1.959 |
| **Practice Location** | **Contrast** | **SE** | **t** | **P-value** |  |  |
| Small town vs. Isolated Rural | 0.180 | 0.005 | 39.300 | 0.000 |  |  |
| Micropolitan vs. Isolated Rural | 0.569 | 0.004 | 133.860 | 0.000 |  |  |
| Metropolitan vs. Isolated rural | 0.740 | 0.004 | 184.430 | 0.000 |  |  |
| Micropolitan vs. Small Town | 0.389 | 0.003 | 123.460 | 0.000 |  |  |
| Metropolitan vs. Small Town | 0.560 | 0.003 | 199.650 | 0.000 |  |  |
| Metropolitan vs. Micropolitan | 0.171 | 0.002 | 81.360 | 0.000 |  |  |

| **Number of outpatient visits, geriatrician** |  |  |  |  |  |  |
| --- | --- | --- | --- | --- | --- | --- |
| N | 27,710,872 |  |  |  |  |  |
| R^2^ | 0.022 |  |  |  |  |  |
|  | **Coefficient** | **SE** | **t** | **P-value** | **LL CI** | **UL CI** |
| Small Town vs. Isolated | 0.000 | 0.001 | -0.510 | 0.609 | -0.002 | 0.001 |
| Micropolitan vs. Isolated | 0.000 | 0.001 | 0.260 | 0.796 | -0.001 | 0.002 |
| Metropolitan vs. Isolated | 0.042 | 0.001 | 52.800 | 0.000 | 0.041 | 0.044 |
| Frail | 0.116 | 0.001 | 188.370 | 0.000 | 0.115 | 0.118 |
| Mean age | 0.002 | 0.000 | 87.470 | 0.000 | 0.002 | 0.002 |
| Under 65 | 0.016 | 0.001 | 23.400 | 0.000 | 0.014 | 0.017 |
| Over 85 | 0.034 | 0.001 | 64.160 | 0.000 | 0.033 | 0.035 |
| Female | 0.009 | 0.000 | 36.980 | 0.000 | 0.009 | 0.010 |
| White vs. Unknown | 0.000 | 0.001 | -0.140 | 0.890 | -0.002 | 0.002 |
| Black vs. Unknown | 0.018 | 0.001 | 15.900 | 0.000 | 0.015 | 0.020 |
| Other vs. Unknown | -0.001 | 0.001 | -0.400 | 0.686 | -0.003 | 0.002 |
| Asian vs. Unknown | 0.004 | 0.001 | 3.100 | 0.002 | 0.002 | 0.007 |
| Hispanic vs. Unknown | -0.010 | 0.001 | -7.370 | 0.000 | -0.013 | -0.008 |
| North American Native vs. Unknown | -0.008 | 0.002 | -4.240 | 0.000 | -0.012 | -0.004 |
| Disabled (original reason for Medicare eligibility) | -0.002 | 0.000 | -4.890 | 0.000 | -0.003 | -0.001 |
| Dual eligible for Medicaid | 0.012 | 0.000 | 32.140 | 0.000 | 0.011 | 0.013 |
| Nursing home | 0.189 | 0.001 | 364.590 | 0.000 | 0.188 | 0.190 |
| Died in the year | -0.055 | 0.001 | -79.360 | 0.000 | -0.056 | -0.053 |
| Median household income (area) | 0.000 | 0.000 | 50.080 | 0.000 | 0.000 | 0.000 |
| Residents under poverty level | 0.001 | 0.000 | 49.610 | 0.000 | 0.001 | 0.001 |
| Number of hierarchical condition categories | 0.012 | 0.000 | 117.000 | 0.000 | 0.012 | 0.012 |
| Coronary artery disease | -0.017 | 0.001 | -26.920 | 0.000 | -0.018 | -0.015 |
| Congestive heart failure | -0.004 | 0.001 | -7.940 | 0.000 | -0.005 | -0.003 |
| Diabetes | -0.012 | 0.000 | -35.770 | 0.000 | -0.012 | -0.011 |
| Cancer | -0.011 | 0.000 | -24.070 | 0.000 | -0.012 | -0.010 |
| Chronic obstructive pulmonary disease | -0.016 | 0.000 | -34.560 | 0.000 | -0.017 | -0.015 |
| End stage renal disease | -0.042 | 0.001 | -35.450 | 0.000 | -0.044 | -0.039 |
| Midwest vs. Northeast | -0.034 | 0.003 | -11.310 | 0.000 | -0.040 | -0.028 |
| South vs. Northeast | -0.016 | 0.003 | -5.300 | 0.000 | -0.022 | -0.010 |
| West vs. Northeast | 0.047 | 0.004 | 11.480 | 0.000 | 0.039 | 0.055 |
| Hospital referral region (suppressed) |  |  |  |  |  |  |
| Constant | -0.183 | 0.004 | -47.760 | 0.000 | -0.191 | -0.176 |
| **Practice Location** | **Margin** | **SE** | **t** | **P-value** | **LL CI** | **UL CI** |
| Isolated Rural | 0.020 | 0.001 | 25.990 | 0.000 | 0.019 | 0.022 |
| Small Town | 0.020 | 0.001 | 37.350 | 0.000 | 0.019 | 0.021 |
| Micropolitan | 0.020 | 0.000 | 53.810 | 0.000 | 0.020 | 0.021 |
| Metropolitan | 0.062 | 0.000 | 435.910 | 0.000 | 0.062 | 0.063 |
| **Practice Location** | **Contrast** | **SE** | **t** | **P-value** |  |  |
| Small town vs. Isolated Rural | 0.000 | 0.001 | -0.510 | 0.609 |  |  |
| Micropolitan vs. Isolated Rural | 0.000 | 0.001 | 0.260 | 0.796 |  |  |
| Metropolitan vs. Isolated rural | 0.042 | 0.001 | 52.800 | 0.000 |  |  |
| Micropolitan vs. Small Town | 0.001 | 0.001 | 1.090 | 0.274 |  |  |
| Metropolitan vs. Small Town | 0.043 | 0.001 | 76.420 | 0.000 |  |  |
| Metropolitan vs. Micropolitan | 0.042 | 0.000 | 100.360 | 0.000 |  |  |

| **Number of outpatient visits, other primary care** |  |  |  |  |  |  |
| --- | --- | --- | --- | --- | --- | --- |
| N | 27,710,872 |  |  |  |  |  |
| R^2^ | 0.013 |  |  |  |  |  |
|  | **Coefficient** | **SE** | **t** | **P-value** | **LL CI** | **UL CI** |
| Small Town vs. Isolated | 0.017 | 0.001 | 12.210 | 0.000 | 0.014 | 0.019 |
| Micropolitan vs. Isolated | -0.058 | 0.001 | -45.840 | 0.000 | -0.060 | -0.055 |
| Metropolitan vs. Isolated | -0.068 | 0.001 | -57.170 | 0.000 | -0.070 | -0.065 |
| Frail | 0.061 | 0.001 | 66.600 | 0.000 | 0.059 | 0.063 |
| Mean age | 0.001 | 0.000 | 18.120 | 0.000 | 0.000 | 0.001 |
| Under 65 | -0.002 | 0.001 | -2.360 | 0.019 | -0.004 | 0.000 |
| Over 85 | -0.015 | 0.001 | -19.500 | 0.000 | -0.017 | -0.014 |
| Female | 0.006 | 0.000 | 16.450 | 0.000 | 0.005 | 0.007 |
| White vs. Unknown | -0.020 | 0.002 | -13.320 | 0.000 | -0.023 | -0.017 |
| Black vs. Unknown | -0.031 | 0.002 | -19.050 | 0.000 | -0.035 | -0.028 |
| Other vs. Unknown | -0.023 | 0.002 | -10.730 | 0.000 | -0.027 | -0.019 |
| Asian vs. Unknown | 0.036 | 0.002 | 17.150 | 0.000 | 0.032 | 0.040 |
| Hispanic vs. Unknown | 0.062 | 0.002 | 29.800 | 0.000 | 0.058 | 0.066 |
| North American Native vs. Unknown | 0.038 | 0.003 | 13.120 | 0.000 | 0.032 | 0.043 |
| Disabled (original reason for Medicare eligibility) | 0.012 | 0.001 | 17.150 | 0.000 | 0.011 | 0.013 |
| Dual eligible for Medicaid | 0.086 | 0.001 | 154.570 | 0.000 | 0.085 | 0.087 |
| Nursing home | 0.068 | 0.001 | 88.370 | 0.000 | 0.066 | 0.069 |
| Died in the year | -0.080 | 0.001 | -78.820 | 0.000 | -0.082 | -0.078 |
| Median household income (area) | 0.000 | 0.000 | -35.610 | 0.000 | 0.000 | 0.000 |
| Residents under poverty level | 0.000 | 0.000 | 10.170 | 0.000 | 0.000 | 0.000 |
| Number of hierarchical condition categories | 0.013 | 0.000 | 83.290 | 0.000 | 0.012 | 0.013 |
| Coronary artery disease | -0.002 | 0.001 | -2.710 | 0.007 | -0.004 | -0.001 |
| Congestive heart failure | -0.012 | 0.001 | -15.850 | 0.000 | -0.013 | -0.010 |
| Diabetes | 0.012 | 0.000 | 25.060 | 0.000 | 0.011 | 0.013 |
| Cancer | -0.016 | 0.001 | -24.380 | 0.000 | -0.017 | -0.015 |
| Chronic obstructive pulmonary disease | 0.022 | 0.001 | 32.390 | 0.000 | 0.020 | 0.023 |
| End stage renal disease | -0.067 | 0.002 | -38.380 | 0.000 | -0.070 | -0.063 |
| Midwest vs. Northeast | -0.003 | 0.004 | -0.660 | 0.511 | -0.012 | 0.006 |
| South vs. Northeast | -0.028 | 0.004 | -6.140 | 0.000 | -0.036 | -0.019 |
| West vs. Northeast | 0.061 | 0.006 | 10.030 | 0.000 | 0.049 | 0.073 |
| Hospital referral region (suppressed) |  |  |  |  |  |  |
| Constant | 0.178 | 0.006 | 31.340 | 0.000 | 0.167 | 0.189 |
| **Practice Location** | **Margin** | **SE** | **t** | **P-value** | **LL CI** | **UL CI** |
| Isolated Rural | 0.173 | 0.001 | 149.370 | 0.000 | 0.170 | 0.175 |
| Small Town | 0.189 | 0.001 | 240.790 | 0.000 | 0.188 | 0.191 |
| Micropolitan | 0.115 | 0.001 | 203.830 | 0.000 | 0.114 | 0.116 |
| Metropolitan | 0.105 | 0.000 | 493.110 | 0.000 | 0.104 | 0.105 |
| **Practice Location** | **Contrast** | **SE** | **t** | **P-value** |  |  |
| Small town vs. Isolated Rural | 0.017 | 0.001 | 12.210 | 0.000 |  |  |
| Micropolitan vs. Isolated Rural | -0.058 | 0.001 | -45.840 | 0.000 |  |  |
| Metropolitan vs. Isolated rural | -0.068 | 0.001 | -57.170 | 0.000 |  |  |
| Micropolitan vs. Small Town | -0.074 | 0.001 | -79.720 | 0.000 |  |  |
| Metropolitan vs. Small Town | -0.084 | 0.001 | -101.850 | 0.000 |  |  |
| Metropolitan vs. Micropolitan | -0.010 | 0.001 | -16.410 | 0.000 |  |  |

| **Number of outpatient visits, specialist** |  |  |  |  |  |  |
| --- | --- | --- | --- | --- | --- | --- |
| N | 27,710,872 |  |  |  |  |  |
| R^2^ | 0.234 |  |  |  |  |  |
|  | **Coefficient** | **SE** | **t** | **P-value** | **LL CI** | **UL CI** |
| Small Town vs. Isolated | 0.038 | 0.008 | 5.010 | 0.000 | 0.023 | 0.053 |
| Micropolitan vs. Isolated | 0.292 | 0.007 | 41.850 | 0.000 | 0.278 | 0.306 |
| Metropolitan vs. Isolated | 0.685 | 0.007 | 103.990 | 0.000 | 0.672 | 0.698 |
| Frail | 1.429 | 0.005 | 280.800 | 0.000 | 1.419 | 1.439 |
| Mean age | 0.019 | 0.000 | 116.870 | 0.000 | 0.019 | 0.020 |
| Under 65 | 0.449 | 0.006 | 81.360 | 0.000 | 0.439 | 0.460 |
| Over 85 | -1.007 | 0.004 | -229.900 | 0.000 | -1.015 | -0.998 |
| Female | 0.229 | 0.002 | 110.320 | 0.000 | 0.225 | 0.233 |
| White vs. Unknown | 0.196 | 0.008 | 23.280 | 0.000 | 0.179 | 0.212 |
| Black vs. Unknown | -0.576 | 0.009 | -62.990 | 0.000 | -0.594 | -0.558 |
| Other vs. Unknown | -0.592 | 0.012 | -49.810 | 0.000 | -0.615 | -0.569 |
| Asian vs. Unknown | -0.861 | 0.012 | -74.210 | 0.000 | -0.884 | -0.838 |
| Hispanic vs. Unknown | -0.565 | 0.012 | -49.110 | 0.000 | -0.588 | -0.543 |
| North American Native vs. Unknown | -0.246 | 0.016 | -15.350 | 0.000 | -0.277 | -0.214 |
| Disabled (original reason for Medicare eligibility) | 0.641 | 0.004 | 165.660 | 0.000 | 0.633 | 0.648 |
| Dual eligible for Medicaid | -0.783 | 0.003 | -253.030 | 0.000 | -0.789 | -0.777 |
| Nursing home | -1.784 | 0.004 | -418.240 | 0.000 | -1.792 | -1.776 |
| Died in the year | -4.466 | 0.006 | -787.410 | 0.000 | -4.477 | -4.455 |
| Median household income (area) | 0.000 | 0.000 | 130.830 | 0.000 | 0.000 | 0.000 |
| Residents under poverty level | 0.001 | 0.000 | 3.530 | 0.000 | 0.000 | 0.001 |
| Number of hierarchical condition categories | 1.207 | 0.001 | 1438.560 | 0.000 | 1.205 | 1.208 |
| Coronary artery disease | 0.099 | 0.005 | 19.510 | 0.000 | 0.089 | 0.109 |
| Congestive heart failure | -0.170 | 0.004 | -40.980 | 0.000 | -0.178 | -0.162 |
| Diabetes | -0.658 | 0.003 | -247.060 | 0.000 | -0.663 | -0.653 |
| Cancer | 3.818 | 0.004 | 1040.730 | 0.000 | 3.811 | 3.825 |
| Chronic obstructive pulmonary disease | -0.378 | 0.004 | -101.410 | 0.000 | -0.386 | -0.371 |
| End stage renal disease | -2.094 | 0.010 | -216.640 | 0.000 | -2.113 | -2.075 |
| Midwest vs. Northeast | 0.162 | 0.025 | 6.590 | 0.000 | 0.114 | 0.211 |
| South vs. Northeast | 0.383 | 0.025 | 15.390 | 0.000 | 0.334 | 0.432 |
| West vs. Northeast | 0.155 | 0.034 | 4.570 | 0.000 | 0.088 | 0.221 |
| Hospital referral region (suppressed) |  |  |  |  |  |  |
| Constant | -0.191 | 0.032 | -6.040 | 0.000 | -0.253 | -0.129 |
| **Practice Location** | **Margin** | **SE** | **t** | **P-value** | **LL CI** | **UL CI** |
| Isolated Rural | 4.305 | 0.006 | 670.920 | 0.000 | 4.293 | 4.318 |
| Small Town | 4.343 | 0.004 | 995.490 | 0.000 | 4.335 | 4.352 |
| Micropolitan | 4.597 | 0.003 | 1467.500 | 0.000 | 4.591 | 4.604 |
| Metropolitan | 4.990 | 0.001 | 4228.060 | 0.000 | 4.988 | 4.992 |
| **Practice Location** | **Contrast** | **SE** | **t** | **P-value** |  |  |
| Small town vs. Isolated Rural | 0.038 | 0.008 | 5.010 | 0.000 |  |  |
| Micropolitan vs. Isolated Rural | 0.292 | 0.007 | 41.850 | 0.000 |  |  |
| Metropolitan vs. Isolated rural | 0.685 | 0.007 | 103.990 | 0.000 |  |  |
| Micropolitan vs. Small Town | 0.254 | 0.005 | 49.210 | 0.000 |  |  |
| Metropolitan vs. Small Town | 0.647 | 0.005 | 140.670 | 0.000 |  |  |
| Metropolitan vs. Micropolitan | 0.393 | 0.003 | 113.970 | 0.000 |  |  |

| **Number of outpatient visits, nurse practitioner, physician assistant, clinical nurse specialist** |  |  |  |  |  |  |
| --- | --- | --- | --- | --- | --- | --- |
| N | 27,710,872 |  |  |  |  |  |
| R^2^ | 0.170 |  |  |  |  |  |
|  | **Coefficient** | **SE** | **t** | **P-value** | **LL CI** | **UL CI** |
| Small Town vs. Isolated | -0.335 | 0.005 | -70.020 | 0.000 | -0.345 | -0.326 |
| Micropolitan vs. Isolated | -0.068 | 0.004 | -15.420 | 0.000 | -0.077 | -0.060 |
| Metropolitan vs. Isolated | -0.097 | 0.004 | -23.070 | 0.000 | -0.105 | -0.088 |
| Frail | 1.530 | 0.003 | 472.940 | 0.000 | 1.523 | 1.536 |
| Mean age | -0.003 | 0.000 | -24.530 | 0.000 | -0.003 | -0.002 |
| Under 65 | 0.136 | 0.004 | 38.780 | 0.000 | 0.129 | 0.143 |
| Over 85 | -0.044 | 0.003 | -15.760 | 0.000 | -0.049 | -0.038 |
| Female | 0.321 | 0.001 | 243.530 | 0.000 | 0.319 | 0.324 |
| White vs. Unknown | 0.138 | 0.005 | 25.780 | 0.000 | 0.127 | 0.148 |
| Black vs. Unknown | -0.107 | 0.006 | -18.420 | 0.000 | -0.118 | -0.096 |
| Other vs. Unknown | -0.144 | 0.008 | -19.030 | 0.000 | -0.159 | -0.129 |
| Asian vs. Unknown | -0.435 | 0.007 | -59.000 | 0.000 | -0.449 | -0.421 |
| Hispanic vs. Unknown | -0.208 | 0.007 | -28.370 | 0.000 | -0.222 | -0.193 |
| North American Native vs. Unknown | 0.841 | 0.010 | 82.610 | 0.000 | 0.821 | 0.861 |
| Disabled (original reason for Medicare eligibility) | 0.222 | 0.002 | 90.390 | 0.000 | 0.217 | 0.227 |
| Dual eligible for Medicaid | 0.662 | 0.002 | 336.580 | 0.000 | 0.658 | 0.666 |
| Nursing home | 2.602 | 0.003 | 960.110 | 0.000 | 2.597 | 2.608 |
| Died in the year | -1.452 | 0.004 | -402.940 | 0.000 | -1.459 | -1.445 |
| Median household income (area) | 0.000 | 0.000 | -9.530 | 0.000 | 0.000 | 0.000 |
| Residents under poverty level | 0.000 | 0.000 | 0.260 | 0.796 | 0.000 | 0.000 |
| Number of hierarchical condition categories | 0.381 | 0.001 | 715.240 | 0.000 | 0.380 | 0.382 |
| Coronary artery disease | -0.347 | 0.003 | -108.060 | 0.000 | -0.353 | -0.341 |
| Congestive heart failure | -0.167 | 0.003 | -63.430 | 0.000 | -0.172 | -0.162 |
| Diabetes | -0.116 | 0.002 | -68.690 | 0.000 | -0.120 | -0.113 |
| Cancer | -0.080 | 0.002 | -34.290 | 0.000 | -0.085 | -0.075 |
| Chronic obstructive pulmonary disease | -0.011 | 0.002 | -4.830 | 0.000 | -0.016 | -0.007 |
| End stage renal disease | -1.306 | 0.006 | -212.660 | 0.000 | -1.318 | -1.294 |
| Midwest vs. Northeast | 0.434 | 0.016 | 27.710 | 0.000 | 0.403 | 0.465 |
| South vs. Northeast | 0.475 | 0.016 | 30.010 | 0.000 | 0.444 | 0.506 |
| West vs. Northeast | 0.787 | 0.022 | 36.590 | 0.000 | 0.745 | 0.829 |
| Hospital referral region (suppressed) |  |  |  |  |  |  |
| Constant | -0.148 | 0.020 | -7.350 | 0.000 | -0.187 | -0.108 |
| **Practice Location** | **Margin** | **SE** | **t** | **P-value** | **LL CI** | **UL CI** |
| Isolated Rural | 1.832 | 0.004 | 449.260 | 0.000 | 1.824 | 1.840 |
| Small Town | 1.497 | 0.003 | 539.840 | 0.000 | 1.491 | 1.502 |
| Micropolitan | 1.764 | 0.002 | 885.890 | 0.000 | 1.760 | 1.768 |
| Metropolitan | 1.735 | 0.001 | 2313.910 | 0.000 | 1.734 | 1.737 |
| **Practice Location** | **Contrast** | **SE** | **t** | **P-value** |  |  |
| Small town vs. Isolated Rural | -0.335 | 0.005 | -70.020 | 0.000 |  |  |
| Micropolitan vs. Isolated Rural | -0.068 | 0.004 | -15.420 | 0.000 |  |  |
| Metropolitan vs. Isolated rural | -0.097 | 0.004 | -23.070 | 0.000 |  |  |
| Micropolitan vs. Small Town | 0.267 | 0.003 | 81.310 | 0.000 |  |  |
| Metropolitan vs. Small Town | 0.239 | 0.003 | 81.700 | 0.000 |  |  |
| Metropolitan vs. Micropolitan | -0.028 | 0.002 | -12.860 | 0.000 |  |  |

| **Number of clinicians encountered, primary care physician** |  |  |  |  |  |  |
| --- | --- | --- | --- | --- | --- | --- |
| N | 27,710,872 |  |  |  |  |  |
| R^2^ | 0.099 |  |  |  |  |  |
|  | **Coefficient** | **SE** | **t** | **P-value** | **LL CI** | **UL CI** |
| Small Town vs. Isolated | 0.081 | 0.001 | 66.040 | 0.000 | 0.078 | 0.083 |
| Micropolitan vs. Isolated | 0.112 | 0.001 | 99.020 | 0.000 | 0.110 | 0.114 |
| Metropolitan vs. Isolated | 0.196 | 0.001 | 183.440 | 0.000 | 0.194 | 0.198 |
| Frail | 0.226 | 0.001 | 273.270 | 0.000 | 0.224 | 0.228 |
| Mean age | 0.003 | 0.000 | 113.270 | 0.000 | 0.003 | 0.003 |
| Under 65 | 0.018 | 0.001 | 20.460 | 0.000 | 0.017 | 0.020 |
| Over 85 | -0.054 | 0.001 | -75.940 | 0.000 | -0.055 | -0.053 |
| Female | 0.106 | 0.000 | 314.780 | 0.000 | 0.105 | 0.107 |
| White vs. Unknown | 0.001 | 0.001 | 1.080 | 0.282 | -0.001 | 0.004 |
| Black vs. Unknown | 0.000 | 0.001 | 0.150 | 0.883 | -0.003 | 0.003 |
| Other vs. Unknown | -0.021 | 0.002 | -10.920 | 0.000 | -0.025 | -0.017 |
| Asian vs. Unknown | 0.014 | 0.002 | 7.450 | 0.000 | 0.010 | 0.018 |
| Hispanic vs. Unknown | 0.001 | 0.002 | 0.560 | 0.575 | -0.003 | 0.005 |
| North American Native vs. Unknown | 0.180 | 0.003 | 69.100 | 0.000 | 0.175 | 0.185 |
| Disabled (original reason for Medicare eligibility) | -0.010 | 0.001 | -16.330 | 0.000 | -0.011 | -0.009 |
| Dual eligible for Medicaid | -0.042 | 0.001 | -82.960 | 0.000 | -0.043 | -0.041 |
| Nursing home | 0.397 | 0.001 | 572.830 | 0.000 | 0.396 | 0.398 |
| Died in the year | -0.436 | 0.001 | -472.950 | 0.000 | -0.438 | -0.434 |
| Median household income (area) | 0.000 | 0.000 | 98.500 | 0.000 | 0.000 | 0.000 |
| Residents under poverty level | 0.000 | 0.000 | 11.520 | 0.000 | 0.000 | 0.000 |
| Number of hierarchical condition categories | 0.092 | 0.000 | 672.060 | 0.000 | 0.091 | 0.092 |
| Coronary artery disease | -0.020 | 0.001 | -23.810 | 0.000 | -0.021 | -0.018 |
| Congestive heart failure | -0.045 | 0.001 | -66.870 | 0.000 | -0.046 | -0.044 |
| Diabetes | 0.023 | 0.000 | 53.690 | 0.000 | 0.022 | 0.024 |
| Cancer | -0.026 | 0.001 | -42.950 | 0.000 | -0.027 | -0.024 |
| Chronic obstructive pulmonary disease | 0.013 | 0.001 | 21.920 | 0.000 | 0.012 | 0.014 |
| End stage renal disease | -0.298 | 0.002 | -190.040 | 0.000 | -0.302 | -0.295 |
| Midwest vs. Northeast | -0.116 | 0.004 | -29.080 | 0.000 | -0.124 | -0.109 |
| South vs. Northeast | -0.072 | 0.004 | -17.790 | 0.000 | -0.080 | -0.064 |
| West vs. Northeast | -0.018 | 0.005 | -3.280 | 0.001 | -0.029 | -0.007 |
| Hospital referral region (suppressed) |  |  |  |  |  |  |
| Constant | 0.626 | 0.005 | 121.930 | 0.000 | 0.616 | 0.636 |
| **Practice Location** | **Margin** | **SE** | **t** | **P-value** | **LL CI** | **UL CI** |
| Isolated Rural | 1.020 | 0.001 | 978.510 | 0.000 | 1.018 | 1.022 |
| Small Town | 1.101 | 0.001 | 1553.330 | 0.000 | 1.100 | 1.102 |
| Micropolitan | 1.132 | 0.001 | 2224.900 | 0.000 | 1.131 | 1.133 |
| Metropolitan | 1.216 | 0.000 | 6343.710 | 0.000 | 1.216 | 1.217 |
| **Practice Location** | **Contrast** | **SE** | **t** | **P-value** |  |  |
| Small town vs. Isolated Rural | 0.081 | 0.001 | 66.040 | 0.000 |  |  |
| Micropolitan vs. Isolated Rural | 0.112 | 0.001 | 99.020 | 0.000 |  |  |
| Metropolitan vs. Isolated rural | 0.196 | 0.001 | 183.440 | 0.000 |  |  |
| Micropolitan vs. Small Town | 0.031 | 0.001 | 37.410 | 0.000 |  |  |
| Metropolitan vs. Small Town | 0.115 | 0.001 | 154.420 | 0.000 |  |  |
| Metropolitan vs. Micropolitan | 0.084 | 0.001 | 150.010 | 0.000 |  |  |

| **Number of clinicians encountered, specialist** |  |  |  |  |  |  |
| --- | --- | --- | --- | --- | --- | --- |
| N | 27,710,872 |  |  |  |  |  |
| R^2^ | 0.225 |  |  |  |  |  |
|  | **Coefficient** | **SE** | **t** | **P-value** | **LL CI** | **UL CI** |
| Small Town vs. Isolated | 0.029 | 0.003 | 10.720 | 0.000 | 0.024 | 0.034 |
| Micropolitan vs. Isolated | 0.119 | 0.003 | 47.510 | 0.000 | 0.114 | 0.124 |
| Metropolitan vs. Isolated | 0.354 | 0.002 | 149.240 | 0.000 | 0.349 | 0.358 |
| Frail | 0.506 | 0.002 | 276.280 | 0.000 | 0.503 | 0.510 |
| Mean age | 0.010 | 0.000 | 166.640 | 0.000 | 0.010 | 0.010 |
| Under 65 | 0.048 | 0.002 | 23.890 | 0.000 | 0.044 | 0.051 |
| Over 85 | -0.512 | 0.002 | -324.610 | 0.000 | -0.515 | -0.508 |
| Female | 0.090 | 0.001 | 121.100 | 0.000 | 0.089 | 0.092 |
| White vs. Unknown | 0.070 | 0.003 | 23.130 | 0.000 | 0.064 | 0.076 |
| Black vs. Unknown | -0.337 | 0.003 | -102.560 | 0.000 | -0.344 | -0.331 |
| Other vs. Unknown | -0.321 | 0.004 | -75.070 | 0.000 | -0.330 | -0.313 |
| Asian vs. Unknown | -0.477 | 0.004 | -114.100 | 0.000 | -0.485 | -0.468 |
| Hispanic vs. Unknown | -0.286 | 0.004 | -69.050 | 0.000 | -0.294 | -0.278 |
| North American Native vs. Unknown | -0.105 | 0.006 | -18.230 | 0.000 | -0.116 | -0.094 |
| Disabled (original reason for Medicare eligibility) | 0.181 | 0.001 | 129.860 | 0.000 | 0.178 | 0.184 |
| Dual eligible for Medicaid | -0.457 | 0.001 | -410.150 | 0.000 | -0.459 | -0.455 |
| Nursing home | -0.744 | 0.002 | -484.530 | 0.000 | -0.747 | -0.741 |
| Died in the year | -1.668 | 0.002 | -816.750 | 0.000 | -1.672 | -1.664 |
| Median household income (area) | 0.000 | 0.000 | 223.110 | 0.000 | 0.000 | 0.000 |
| Residents under poverty level | 0.000 | 0.000 | -1.100 | 0.271 | 0.000 | 0.000 |
| Number of hierarchical condition categories | 0.405 | 0.000 | 1341.380 | 0.000 | 0.404 | 0.406 |
| Coronary artery disease | 0.164 | 0.002 | 90.370 | 0.000 | 0.161 | 0.168 |
| Congestive heart failure | -0.031 | 0.001 | -20.580 | 0.000 | -0.034 | -0.028 |
| Diabetes | -0.197 | 0.001 | -205.390 | 0.000 | -0.199 | -0.195 |
| Cancer | 1.146 | 0.001 | 868.140 | 0.000 | 1.144 | 1.149 |
| Chronic obstructive pulmonary disease | -0.102 | 0.001 | -75.660 | 0.000 | -0.104 | -0.099 |
| End stage renal disease | -0.351 | 0.003 | -100.930 | 0.000 | -0.358 | -0.344 |
| Midwest vs. Northeast | -0.044 | 0.009 | -4.910 | 0.000 | -0.061 | -0.026 |
| South vs. Northeast | 0.078 | 0.009 | 8.700 | 0.000 | 0.060 | 0.095 |
| West vs. Northeast | -0.043 | 0.012 | -3.500 | 0.000 | -0.066 | -0.019 |
| Hospital referral region (suppressed) |  |  |  |  |  |  |
| Constant | 0.388 | 0.011 | 34.140 | 0.000 | 0.366 | 0.411 |
| **Practice Location** | **Margin** | **SE** | **t** | **P-value** | **LL CI** | **UL CI** |
| Isolated Rural | 1.963 | 0.002 | 849.650 | 0.000 | 1.958 | 1.967 |
| Small Town | 1.992 | 0.002 | 1268.230 | 0.000 | 1.989 | 1.995 |
| Micropolitan | 2.082 | 0.001 | 1846.200 | 0.000 | 2.080 | 2.084 |
| Metropolitan | 2.316 | 0.000 | 5452.300 | 0.000 | 2.316 | 2.317 |
| **Practice Location** | **Contrast** | **SE** | **t** | **P-value** |  |  |
| Small town vs. Isolated Rural | 0.029 | 0.003 | 10.720 | 0.000 |  |  |
| Micropolitan vs. Isolated Rural | 0.119 | 0.003 | 47.510 | 0.000 |  |  |
| Metropolitan vs. Isolated rural | 0.354 | 0.002 | 149.240 | 0.000 |  |  |
| Micropolitan vs. Small Town | 0.090 | 0.002 | 48.530 | 0.000 |  |  |
| Metropolitan vs. Small Town | 0.325 | 0.002 | 196.100 | 0.000 |  |  |
| Metropolitan vs. Micropolitan | 0.234 | 0.001 | 188.970 | 0.000 |  |  |

| **Number of clinicians encountered, nurse practitioner, physician assistant, clinical nurse specialist** |  |  |  |  |  |  |
| --- | --- | --- | --- | --- | --- | --- |
| N | 27,710,872 |  |  |  |  |  |
| R^2^ | 0.149 |  |  |  |  |  |
|  | **Coefficient** | **SE** | **t** | **P-value** | **LL CI** | **UL CI** |
| Small Town vs. Isolated | -0.076 | 0.001 | -52.510 | 0.000 | -0.078 | -0.073 |
| Micropolitan vs. Isolated | 0.052 | 0.001 | 39.270 | 0.000 | 0.050 | 0.055 |
| Metropolitan vs. Isolated | 0.068 | 0.001 | 53.690 | 0.000 | 0.065 | 0.070 |
| Frail | 0.323 | 0.001 | 331.930 | 0.000 | 0.321 | 0.324 |
| Mean age | -0.003 | 0.000 | -88.710 | 0.000 | -0.003 | -0.003 |
| Under 65 | 0.031 | 0.001 | 29.760 | 0.000 | 0.029 | 0.033 |
| Over 85 | -0.043 | 0.001 | -51.500 | 0.000 | -0.045 | -0.041 |
| Female | 0.144 | 0.000 | 363.250 | 0.000 | 0.143 | 0.145 |
| White vs. Unknown | 0.060 | 0.002 | 37.320 | 0.000 | 0.057 | 0.063 |
| Black vs. Unknown | -0.072 | 0.002 | -41.380 | 0.000 | -0.076 | -0.069 |
| Other vs. Unknown | -0.083 | 0.002 | -36.400 | 0.000 | -0.087 | -0.078 |
| Asian vs. Unknown | -0.142 | 0.002 | -64.030 | 0.000 | -0.146 | -0.137 |
| Hispanic vs. Unknown | -0.050 | 0.002 | -22.630 | 0.000 | -0.054 | -0.045 |
| North American Native vs. Unknown | 0.264 | 0.003 | 86.350 | 0.000 | 0.258 | 0.270 |
| Disabled (original reason for Medicare eligibility) | 0.059 | 0.001 | 79.760 | 0.000 | 0.057 | 0.060 |
| Dual eligible for Medicaid | 0.035 | 0.001 | 58.900 | 0.000 | 0.034 | 0.036 |
| Nursing home | 0.466 | 0.001 | 572.280 | 0.000 | 0.464 | 0.468 |
| Died in the year | -0.460 | 0.001 | -425.070 | 0.000 | -0.462 | -0.458 |
| Median household income (area) | 0.000 | 0.000 | -3.160 | 0.002 | 0.000 | 0.000 |
| Residents under poverty level | -0.001 | 0.000 | -30.410 | 0.000 | -0.001 | -0.001 |
| Number of hierarchical condition categories | 0.119 | 0.000 | 742.950 | 0.000 | 0.119 | 0.119 |
| Coronary artery disease | -0.010 | 0.001 | -10.880 | 0.000 | -0.012 | -0.009 |
| Congestive heart failure | -0.028 | 0.001 | -34.850 | 0.000 | -0.029 | -0.026 |
| Diabetes | -0.055 | 0.001 | -107.750 | 0.000 | -0.056 | -0.054 |
| Cancer | 0.078 | 0.001 | 111.820 | 0.000 | 0.077 | 0.080 |
| Chronic obstructive pulmonary disease | -0.003 | 0.001 | -4.500 | 0.000 | -0.005 | -0.002 |
| End stage renal disease | -0.340 | 0.002 | -184.100 | 0.000 | -0.343 | -0.336 |
| Midwest vs. Northeast | 0.187 | 0.005 | 39.840 | 0.000 | 0.178 | 0.197 |
| South vs. Northeast | 0.184 | 0.005 | 38.820 | 0.000 | 0.175 | 0.194 |
| West vs. Northeast | 0.167 | 0.006 | 25.870 | 0.000 | 0.154 | 0.180 |
| Hospital referral region (suppressed) |  |  |  |  |  |  |
| Constant | 0.250 | 0.006 | 41.470 | 0.000 | 0.238 | 0.262 |
| **Practice Location** | **Margin** | **SE** | **t** | **P-value** | **LL CI** | **UL CI** |
| Isolated Rural | 0.709 | 0.001 | 578.520 | 0.000 | 0.706 | 0.711 |
| Small Town | 0.633 | 0.001 | 760.200 | 0.000 | 0.631 | 0.635 |
| Micropolitan | 0.761 | 0.001 | 1272.480 | 0.000 | 0.760 | 0.762 |
| Metropolitan | 0.776 | 0.000 | 3445.000 | 0.000 | 0.776 | 0.777 |
| **Practice Location** | **Contrast** | **SE** | **t** | **p-value** |  |  |
| Small town vs. Isolated Rural | -0.076 | 0.001 | -52.510 | 0.000 |  |  |
| Micropolitan vs. Isolated Rural | 0.052 | 0.001 | 39.270 | 0.000 |  |  |
| Metropolitan vs. Isolated rural | 0.068 | 0.001 | 53.690 | 0.000 |  |  |
| Micropolitan vs. Small Town | 0.128 | 0.001 | 129.630 | 0.000 |  |  |
| Metropolitan vs. Small Town | 0.143 | 0.001 | 162.900 | 0.000 |  |  |
| Metropolitan vs. Micropolitan | 0.015 | 0.001 | 23.080 | 0.000 |  |  |

| **Total payments** |  |  |  |  |  |  |
| --- | --- | --- | --- | --- | --- | --- |
| N | 27,710,872 |  |  |  |  |  |
| R^2^ | 0.482 |  |  |  |  |  |
|  | **Coefficient** | **SE** | **t** | **P-value** | **LL CI** | **UL CI** |
| Small Town vs. Isolated | -253.509 | 24.016 | -10.560 | 0.000 | -300.580 | -206.439 |
| Micropolitan vs. Isolated | -717.413 | 22.238 | -32.260 | 0.000 | -760.999 | -673.827 |
| Metropolitan vs. Isolated | -724.913 | 20.986 | -34.540 | 0.000 | -766.044 | -683.781 |
| Frail | 8610.253 | 16.218 | 530.910 | 0.000 | 8578.466 | 8642.039 |
| Mean age | -94.793 | 0.523 | -181.230 | 0.000 | -95.818 | -93.768 |
| Under 65 | -720.311 | 17.603 | -40.920 | 0.000 | -754.812 | -685.810 |
| Over 85 | -1600.596 | 13.950 | -114.730 | 0.000 | -1627.938 | -1573.253 |
| Female | 532.839 | 6.612 | 80.590 | 0.000 | 519.880 | 545.798 |
| White vs. Unknown | -336.034 | 26.787 | -12.540 | 0.000 | -388.535 | -283.533 |
| Black vs. Unknown | -211.807 | 29.126 | -7.270 | 0.000 | -268.894 | -154.720 |
| Other vs. Unknown | -523.705 | 37.881 | -13.830 | 0.000 | -597.950 | -449.460 |
| Asian vs. Unknown | -216.360 | 36.973 | -5.850 | 0.000 | -288.825 | -143.896 |
| Hispanic vs. Unknown | 550.257 | 36.682 | 15.000 | 0.000 | 478.361 | 622.153 |
| North American Native vs. Unknown | 713.603 | 51.037 | 13.980 | 0.000 | 613.573 | 813.634 |
| Disabled (original reason for Medicare eligibility) | -1269.144 | 12.324 | -102.990 | 0.000 | -1293.297 | -1244.990 |
| Dual eligible for Medicaid | -2425.750 | 9.861 | -245.990 | 0.000 | -2445.077 | -2406.423 |
| Nursing home | 10510.610 | 13.592 | 773.300 | 0.000 | 10483.970 | 10537.250 |
| Died in the year | 517.291 | 18.074 | 28.620 | 0.000 | 481.866 | 552.717 |
| Median household income (area) | 0.001 | 0.000 | 6.120 | 0.000 | 0.001 | 0.002 |
| Residents under poverty level | 6.619 | 0.464 | 14.260 | 0.000 | 5.709 | 7.529 |
| Number of hierarchical condition categories | 7341.848 | 2.673 | 2746.540 | 0.000 | 7336.609 | 7347.087 |
| Coronary artery disease | 881.256 | 16.104 | 54.720 | 0.000 | 849.693 | 912.819 |
| Congestive heart failure | -3356.269 | 13.205 | -254.160 | 0.000 | -3382.151 | -3330.387 |
| Diabetes | -6758.134 | 8.486 | -796.350 | 0.000 | -6774.767 | -6741.501 |
| Cancer | 1662.866 | 11.689 | 142.250 | 0.000 | 1639.956 | 1685.777 |
| Chronic obstructive pulmonary disease | -4761.846 | 11.892 | -400.410 | 0.000 | -4785.155 | -4738.537 |
| End stage renal disease | 22847.590 | 30.802 | 741.770 | 0.000 | 22787.220 | 22907.960 |
| Midwest vs. Northeast | 255.582 | 78.504 | 3.260 | 0.001 | 101.718 | 409.447 |
| South vs. Northeast | 325.117 | 79.289 | 4.100 | 0.000 | 169.714 | 480.521 |
| West vs. Northeast | 105.388 | 107.857 | 0.980 | 0.329 | -106.008 | 316.783 |
| Hospital referral region (suppressed) |  |  |  |  |  |  |
| Constant | 7147.123 | 100.657 | 71.000 | 0.000 | 6949.838 | 7344.407 |
| **Practice Location** | **Margin** | **SE** | **t** | **P-value** | **LL CI** | **UL CI** |
| Isolated Rural | 11501.400 | 20.448 | 562.480 | 0.000 | 11461.330 | 11541.480 |
| Small Town | 11247.890 | 13.902 | 809.090 | 0.000 | 11220.650 | 11275.140 |
| Micropolitan | 10783.990 | 9.983 | 1080.270 | 0.000 | 10764.420 | 10803.560 |
| Metropolitan | 10776.490 | 3.761 | 2865.420 | 0.000 | 10769.120 | 10783.860 |
| **Practice Location** | **Contrast** | **SE** | **t** | **P-value** |  |  |
| Small town vs. Isolated Rural | -253.509 | 24.016 | -10.560 | 0.000 |  |  |
| Micropolitan vs. Isolated Rural | -717.413 | 22.238 | -32.260 | 0.000 |  |  |
| Metropolitan vs. Isolated rural | -724.913 | 20.986 | -34.540 | 0.000 |  |  |
| Micropolitan vs. Small Town | -463.904 | 16.466 | -28.170 | 0.000 |  |  |
| Metropolitan vs. Small Town | -471.404 | 14.658 | -32.160 | 0.000 |  |  |
| Metropolitan vs. Micropolitan | -7.500 | 10.983 | -0.680 | 0.495 |  |  |

| **Acute care payments** |  |  |  |  |  |  |
| --- | --- | --- | --- | --- | --- | --- |
| N | 27,710,872 |  |  |  |  |  |
| R^2^ | 0.383 |  |  |  |  |  |
|  | **Coefficient** | **SE** | **t** | **P-value** | **LL CI** | **UL CI** |
| Small Town vs. Isolated | -104.118 | 13.954 | -7.460 | 0.000 | -131.467 | -76.768 |
| Micropolitan vs. Isolated | -321.342 | 12.921 | -24.870 | 0.000 | -346.667 | -296.017 |
| Metropolitan vs. Isolated | -454.751 | 12.193 | -37.300 | 0.000 | -478.649 | -430.852 |
| Frail | 1790.837 | 9.423 | 190.050 | 0.000 | 1772.368 | 1809.305 |
| Mean age | -75.947 | 0.304 | -249.900 | 0.000 | -76.543 | -75.351 |
| Under 65 | -704.261 | 10.228 | -68.860 | 0.000 | -724.307 | -684.214 |
| Over 85 | -1175.311 | 8.106 | -145.000 | 0.000 | -1191.198 | -1159.425 |
| Female | 40.260 | 3.842 | 10.480 | 0.000 | 32.731 | 47.790 |
| White vs. Unknown | -233.929 | 15.564 | -15.030 | 0.000 | -264.434 | -203.425 |
| Black vs. Unknown | 74.101 | 16.923 | 4.380 | 0.000 | 40.932 | 107.270 |
| Other vs. Unknown | 104.823 | 22.010 | 4.760 | 0.000 | 61.685 | 147.962 |
| Asian vs. Unknown | 449.704 | 21.482 | 20.930 | 0.000 | 407.600 | 491.808 |
| Hispanic vs. Unknown | 609.946 | 21.313 | 28.620 | 0.000 | 568.172 | 651.719 |
| North American Native vs. Unknown | 535.434 | 29.654 | 18.060 | 0.000 | 477.313 | 593.554 |
| Disabled (original reason for Medicare eligibility) | -1278.074 | 7.160 | -178.490 | 0.000 | -1292.108 | -1264.040 |
| Dual eligible for Medicaid | -1231.287 | 5.730 | -214.900 | 0.000 | -1242.517 | -1220.058 |
| Nursing home | 2227.358 | 7.897 | 282.040 | 0.000 | 2211.880 | 2242.837 |
| Died in the year | 3194.942 | 10.502 | 304.230 | 0.000 | 3174.359 | 3215.525 |
| Median household income (area) | -0.001 | 0.000 | -7.780 | 0.000 | -0.001 | -0.001 |
| Residents under poverty level | 6.338 | 0.270 | 23.490 | 0.000 | 5.809 | 6.866 |
| Number of hierarchical condition categories | 3948.023 | 1.553 | 2541.920 | 0.000 | 3944.979 | 3951.068 |
| Coronary artery disease | 1751.263 | 9.357 | 187.160 | 0.000 | 1732.924 | 1769.602 |
| Congestive heart failure | -1485.405 | 7.673 | -193.600 | 0.000 | -1500.443 | -1470.367 |
| Diabetes | -4061.568 | 4.931 | -823.710 | 0.000 | -4071.232 | -4051.903 |
| Cancer | -2319.641 | 6.792 | -341.530 | 0.000 | -2332.953 | -2306.330 |
| Chronic obstructive pulmonary disease | -2854.199 | 6.910 | -413.060 | 0.000 | -2867.742 | -2840.656 |
| End stage renal disease | 2800.451 | 17.897 | 156.480 | 0.000 | 2765.374 | 2835.527 |
| Midwest vs. Northeast | 287.329 | 45.613 | 6.300 | 0.000 | 197.929 | 376.729 |
| South vs. Northeast | 196.000 | 46.069 | 4.250 | 0.000 | 105.706 | 286.294 |
| West vs. Northeast | 245.476 | 62.668 | 3.920 | 0.000 | 122.649 | 368.303 |
| Hospital referral region (suppressed) |  |  |  |  |  |  |
| Constant | 4385.817 | 58.485 | 74.990 | 0.000 | 4271.189 | 4500.445 |
| **Practice Location** | **Margin** | **SE** | **t** | **P-value** | **LL CI** | **UL CI** |
| Isolated Rural | 3862.229 | 11.881 | 325.080 | 0.000 | 3838.943 | 3885.515 |
| Small Town | 3758.112 | 8.077 | 465.260 | 0.000 | 3742.280 | 3773.943 |
| Micropolitan | 3540.887 | 5.800 | 610.470 | 0.000 | 3529.519 | 3552.256 |
| Metropolitan | 3407.479 | 2.185 | 1559.360 | 0.000 | 3403.196 | 3411.762 |
| **Practice Location** | **Contrast** | **SE** | **t** | **P-value** |  |  |
| Small town vs. Isolated Rural | -104.118 | 13.954 | -7.460 | 0.000 |  |  |
| Micropolitan vs. Isolated Rural | -321.342 | 12.921 | -24.870 | 0.000 |  |  |
| Metropolitan vs. Isolated rural | -454.751 | 12.193 | -37.300 | 0.000 |  |  |
| Micropolitan vs. Small Town | -217.224 | 9.567 | -22.710 | 0.000 |  |  |
| Metropolitan vs. Small Town | -350.633 | 8.517 | -41.170 | 0.000 |  |  |
| Metropolitan vs. Micropolitan | -133.409 | 6.381 | -20.910 | 0.000 |  |  |

| **Procedures payments** |  |  |  |  |  |  |
| --- | --- | --- | --- | --- | --- | --- |
| N | 27,710,872 |  |  |  |  |  |
| R^2^ | 0.396 |  |  |  |  |  |
|  | **Coefficient** | **SE** | **t** | **P-value** | **LL CI** | **UL CI** |
| Small Town vs. Isolated | 3.102 | 4.050 | 0.770 | 0.444 | -4.835 | 11.040 |
| Micropolitan vs. Isolated | 2.919 | 3.750 | 0.780 | 0.436 | -4.431 | 10.270 |
| Metropolitan vs. Isolated | 14.614 | 3.539 | 4.130 | 0.000 | 7.678 | 21.551 |
| Frail | 685.798 | 2.735 | 250.760 | 0.000 | 680.437 | 691.158 |
| Mean age | -0.177 | 0.088 | -2.000 | 0.045 | -0.350 | -0.004 |
| Under 65 | -173.573 | 2.968 | -58.470 | 0.000 | -179.391 | -167.754 |
| Over 85 | -376.480 | 2.353 | -160.030 | 0.000 | -381.091 | -371.869 |
| Female | -75.053 | 1.115 | -67.310 | 0.000 | -77.239 | -72.868 |
| White vs. Unknown | 46.970 | 4.517 | 10.400 | 0.000 | 38.116 | 55.823 |
| Black vs. Unknown | -176.915 | 4.912 | -36.020 | 0.000 | -186.542 | -167.288 |
| Other vs. Unknown | -214.476 | 6.388 | -33.570 | 0.000 | -226.997 | -201.956 |
| Asian vs. Unknown | -332.095 | 6.235 | -53.260 | 0.000 | -344.315 | -319.875 |
| Hispanic vs. Unknown | -111.215 | 6.186 | -17.980 | 0.000 | -123.340 | -99.091 |
| North American Native vs. Unknown | -132.633 | 8.607 | -15.410 | 0.000 | -149.502 | -115.764 |
| Disabled (original reason for Medicare eligibility) | -25.849 | 2.078 | -12.440 | 0.000 | -29.922 | -21.776 |
| Dual eligible for Medicaid | -105.946 | 1.663 | -63.710 | 0.000 | -109.205 | -102.687 |
| Nursing home | 135.319 | 2.292 | 59.040 | 0.000 | 130.827 | 139.811 |
| Died in the year | -1513.066 | 3.048 | -496.410 | 0.000 | -1519.040 | -1507.092 |
| Median household income (area) | 0.001 | 0.000 | 33.510 | 0.000 | 0.001 | 0.001 |
| Residents under poverty level | -1.831 | 0.078 | -23.390 | 0.000 | -1.985 | -1.678 |
| Number of hierarchical condition categories | 398.985 | 0.451 | 885.090 | 0.000 | 398.102 | 399.869 |
| Coronary artery disease | 454.316 | 2.716 | 167.290 | 0.000 | 448.993 | 459.639 |
| Congestive heart failure | -264.179 | 2.227 | -118.630 | 0.000 | -268.543 | -259.814 |
| Diabetes | -219.775 | 1.431 | -153.570 | 0.000 | -222.580 | -216.970 |
| Cancer | 1446.407 | 1.971 | 733.750 | 0.000 | 1442.543 | 1450.270 |
| Chronic obstructive pulmonary disease | -336.048 | 2.006 | -167.560 | 0.000 | -339.979 | -332.117 |
| End stage renal disease | 17519.400 | 5.194 | 3372.840 | 0.000 | 17509.220 | 17529.580 |
| Midwest vs. Northeast | 7.212 | 13.239 | 0.540 | 0.586 | -18.735 | 33.159 |
| South vs. Northeast | -3.770 | 13.371 | -0.280 | 0.778 | -29.976 | 22.437 |
| West vs. Northeast | 38.664 | 18.189 | 2.130 | 0.034 | 3.016 | 74.313 |
| Hospital referral region (suppressed) |  |  |  |  |  |  |
| Constant | 634.948 | 16.974 | 37.410 | 0.000 | 601.679 | 668.217 |
| **Practice Location** | **Margin** | **SE** | **t** | **P-value** | **LL CI** | **UL CI** |
| Isolated Rural | 1568.130 | 3.448 | 454.760 | 0.000 | 1561.371 | 1574.888 |
| Small Town | 1571.232 | 2.344 | 670.220 | 0.000 | 1566.637 | 1575.827 |
| Micropolitan | 1571.049 | 1.683 | 933.240 | 0.000 | 1567.750 | 1574.349 |
| Metropolitan | 1582.744 | 0.634 | 2495.580 | 0.000 | 1581.501 | 1583.987 |
| **Practice Location** | **Contrast** | **SE** | **t** | **P-value** |  |  |
| Small town vs. Isolated Rural | 3.102 | 4.050 | 0.770 | 0.444 |  |  |
| Micropolitan vs. Isolated Rural | 2.919 | 3.750 | 0.780 | 0.436 |  |  |
| Metropolitan vs. Isolated rural | 14.614 | 3.539 | 4.130 | 0.000 |  |  |
| Micropolitan vs. Small Town | -0.183 | 2.777 | -0.070 | 0.947 |  |  |
| Metropolitan vs. Small Town | 11.512 | 2.472 | 4.660 | 0.000 |  |  |
| Metropolitan vs. Micropolitan | 11.695 | 1.852 | 6.310 | 0.000 |  |  |

| **Evaluation and management payments** |  |  |  |  |  |  |
| --- | --- | --- | --- | --- | --- | --- |
| N | 27,710,872 |  |  |  |  |  |
| R^2^ | 0.224 |  |  |  |  |  |
|  | **Coefficient** | **SE** | **t** | **P-value** | **LL CI** | **UL CI** |
| Small Town vs. Isolated | 27.698 | 3.844 | 7.210 | 0.000 | 20.164 | 35.232 |
| Micropolitan vs. Isolated | 61.508 | 3.559 | 17.280 | 0.000 | 54.532 | 68.484 |
| Metropolitan vs. Isolated | 130.453 | 3.359 | 38.840 | 0.000 | 123.870 | 137.037 |
| Frail | 831.705 | 2.596 | 320.400 | 0.000 | 826.618 | 836.793 |
| Mean age | -6.599 | 0.084 | -78.830 | 0.000 | -6.763 | -6.435 |
| Under 65 | 30.987 | 2.818 | 11.000 | 0.000 | 25.465 | 36.510 |
| Over 85 | -153.271 | 2.233 | -68.640 | 0.000 | -157.647 | -148.894 |
| Female | 113.490 | 1.058 | 107.240 | 0.000 | 111.415 | 115.564 |
| White vs. Unknown | 11.897 | 4.287 | 2.770 | 0.006 | 3.493 | 20.300 |
| Black vs. Unknown | -16.824 | 4.662 | -3.610 | 0.000 | -25.961 | -7.686 |
| Other vs. Unknown | -75.247 | 6.063 | -12.410 | 0.000 | -87.130 | -63.363 |
| Asian vs. Unknown | -91.461 | 5.918 | -15.460 | 0.000 | -103.060 | -79.862 |
| Hispanic vs. Unknown | -61.704 | 5.871 | -10.510 | 0.000 | -73.212 | -50.197 |
| North American Native vs. Unknown | -18.326 | 8.169 | -2.240 | 0.025 | -34.337 | -2.315 |
| Disabled (original reason for Medicare eligibility) | -57.817 | 1.973 | -29.310 | 0.000 | -61.683 | -53.951 |
| Dual eligible for Medicaid | -45.777 | 1.578 | -29.000 | 0.000 | -48.870 | -42.683 |
| Nursing home | 410.835 | 2.176 | 188.850 | 0.000 | 406.571 | 415.099 |
| Died in the year | -335.289 | 2.893 | -115.900 | 0.000 | -340.960 | -329.619 |
| Median household income (area) | 0.001 | 0.000 | 30.870 | 0.000 | 0.001 | 0.001 |
| Residents under poverty level | 0.093 | 0.074 | 1.250 | 0.211 | -0.053 | 0.239 |
| Number of hierarchical condition categories | 698.949 | 0.428 | 1633.590 | 0.000 | 698.111 | 699.788 |
| Coronary artery disease | 21.238 | 2.578 | 8.240 | 0.000 | 16.186 | 26.290 |
| Congestive heart failure | -361.934 | 2.114 | -171.240 | 0.000 | -366.077 | -357.792 |
| Diabetes | -546.562 | 1.358 | -402.380 | 0.000 | -549.224 | -543.899 |
| Cancer | 600.131 | 1.871 | 320.750 | 0.000 | 596.464 | 603.798 |
| Chronic obstructive pulmonary disease | -301.144 | 1.904 | -158.200 | 0.000 | -304.875 | -297.413 |
| End stage renal disease | -349.710 | 4.930 | -70.930 | 0.000 | -359.373 | -340.047 |
| Midwest vs. Northeast | -76.191 | 12.565 | -6.060 | 0.000 | -100.819 | -51.564 |
| South vs. Northeast | -16.520 | 12.691 | -1.300 | 0.193 | -41.394 | 8.354 |
| West vs. Northeast | 47.684 | 17.264 | 2.760 | 0.006 | 13.848 | 81.520 |
| Hospital referral region (suppressed) |  |  |  |  |  |  |
| Constant | 389.986 | 16.111 | 24.210 | 0.000 | 358.409 | 421.563 |
| **Practice Location** | **Margin** | **SE** | **t** | **P-value** | **LL CI** | **UL CI** |
| Isolated Rural | 1205.707 | 3.273 | 368.390 | 0.000 | 1199.293 | 1212.122 |
| Small Town | 1233.406 | 2.225 | 554.300 | 0.000 | 1229.044 | 1237.767 |
| Micropolitan | 1267.215 | 1.598 | 793.090 | 0.000 | 1264.084 | 1270.347 |
| Metropolitan | 1336.161 | 0.602 | 2219.660 | 0.000 | 1334.981 | 1337.341 |
| **Practice Location** | **Contrast** | **SE** | **t** | **P-value** |  |  |
| Small town vs. Isolated Rural | 27.698 | 3.844 | 7.210 | 0.000 |  |  |
| Micropolitan vs. Isolated Rural | 61.508 | 3.559 | 17.280 | 0.000 |  |  |
| Metropolitan vs. Isolated rural | 130.453 | 3.359 | 38.840 | 0.000 |  |  |
| Micropolitan vs. Small Town | 33.810 | 2.636 | 12.830 | 0.000 |  |  |
| Metropolitan vs. Small Town | 102.755 | 2.346 | 43.800 | 0.000 |  |  |
| Metropolitan vs. Micropolitan | 68.946 | 1.758 | 39.220 | 0.000 |  |  |

| **Other payments** |  |  |  |  |  |  |
| --- | --- | --- | --- | --- | --- | --- |
| N | 27,710,872 |  |  |  |  |  |
| R^2^ | 0.235 |  |  |  |  |  |
|  | **Coefficient** | **SE** | **t** | **P-value** | **LL CI** | **UL CI** |
| Small Town vs. Isolated | -27.398 | 14.101 | -1.940 | 0.052 | -55.035 | 0.239 |
| Micropolitan vs. Isolated | -102.980 | 13.057 | -7.890 | 0.000 | -128.571 | -77.389 |
| Metropolitan vs. Isolated | -13.915 | 12.322 | -1.130 | 0.259 | -38.065 | 10.234 |
| Frail | 4747.556 | 9.522 | 498.580 | 0.000 | 4728.893 | 4766.219 |
| Mean age | -3.014 | 0.307 | -9.810 | 0.000 | -3.616 | -2.412 |
| Under 65 | 71.700 | 10.335 | 6.940 | 0.000 | 51.443 | 91.956 |
| Over 85 | 126.617 | 8.191 | 15.460 | 0.000 | 110.563 | 142.671 |
| Female | 475.229 | 3.882 | 122.420 | 0.000 | 467.621 | 482.838 |
| White vs. Unknown | -169.103 | 15.727 | -10.750 | 0.000 | -199.928 | -138.278 |
| Black vs. Unknown | -87.328 | 17.101 | -5.110 | 0.000 | -120.846 | -53.810 |
| Other vs. Unknown | -360.174 | 22.241 | -16.190 | 0.000 | -403.766 | -316.582 |
| Asian vs. Unknown | -285.680 | 21.708 | -13.160 | 0.000 | -328.227 | -243.133 |
| Hispanic vs. Unknown | 114.928 | 21.538 | 5.340 | 0.000 | 72.716 | 157.141 |
| North American Native vs. Unknown | 336.420 | 29.966 | 11.230 | 0.000 | 277.688 | 395.151 |
| Disabled (original reason for Medicare eligibility) | 141.476 | 7.236 | 19.550 | 0.000 | 127.294 | 155.657 |
| Dual eligible for Medicaid | -1000.543 | 5.790 | -172.810 | 0.000 | -1011.891 | -989.196 |
| Nursing home | 6841.158 | 7.980 | 857.260 | 0.000 | 6825.517 | 6856.799 |
| Died in the year | -447.947 | 10.612 | -42.210 | 0.000 | -468.746 | -427.147 |
| Median household income (area) | 0.000 | 0.000 | 3.190 | 0.001 | 0.000 | 0.001 |
| Residents under poverty level | 1.164 | 0.273 | 4.270 | 0.000 | 0.630 | 1.698 |
| Number of hierarchical condition categories | 2028.434 | 1.569 | 1292.420 | 0.000 | 2025.358 | 2031.510 |
| Coronary artery disease | -1074.253 | 9.455 | -113.620 | 0.000 | -1092.784 | -1055.721 |
| Congestive heart failure | -907.104 | 7.753 | -116.990 | 0.000 | -922.301 | -891.908 |
| Diabetes | -1651.716 | 4.983 | -331.490 | 0.000 | -1661.481 | -1641.950 |
| Cancer | 2253.656 | 6.863 | 328.370 | 0.000 | 2240.204 | 2267.107 |
| Chronic obstructive pulmonary disease | -1013.263 | 6.983 | -145.110 | 0.000 | -1026.949 | -999.578 |
| End stage renal disease | 3527.708 | 18.085 | 195.070 | 0.000 | 3492.263 | 3563.154 |
| Midwest vs. Northeast | 102.819 | 46.092 | 2.230 | 0.026 | 12.480 | 193.159 |
| South vs. Northeast | 213.252 | 46.553 | 4.580 | 0.000 | 122.009 | 304.495 |
| West vs. Northeast | 247.088 | 63.327 | 3.900 | 0.000 | 122.970 | 371.206 |
| Hospital referral region (suppressed) |  |  |  |  |  |  |
| Constant | 691.267 | 59.099 | 11.700 | 0.000 | 575.435 | 807.100 |
| **Practice Location** | **Margin** | **SE** | **t** | **P-value** | **LL CI** | **UL CI** |
| Isolated Rural | 4210.675 | 12.006 | 350.720 | 0.000 | 4187.144 | 4234.206 |
| Small Town | 4183.277 | 8.162 | 512.510 | 0.000 | 4167.279 | 4199.275 |
| Micropolitan | 4107.695 | 5.861 | 700.830 | 0.000 | 4096.207 | 4119.182 |
| Metropolitan | 4196.759 | 2.208 | 1900.580 | 0.000 | 4192.432 | 4201.087 |
| **Practice Location** | **Contrast** | **SE** | **t** | **P-value** |  |  |
| Small town vs. Isolated Rural | -27.398 | 14.101 | -1.940 | 0.052 |  |  |
| Micropolitan vs. Isolated Rural | -102.980 | 13.057 | -7.890 | 0.000 |  |  |
| Metropolitan vs. Isolated rural | -13.915 | 12.322 | -1.130 | 0.259 |  |  |
| Micropolitan vs. Small Town | -75.583 | 9.668 | -7.820 | 0.000 |  |  |
| Metropolitan vs. Small Town | 13.482 | 8.606 | 1.570 | 0.117 |  |  |
| Metropolitan vs. Micropolitan | 89.065 | 6.448 | 13.810 | 0.000 |  |  |

| **Practices where isolated beneficiaries get care** |  |  |  |  |  |  |  |  |  |  |  |  |  |  |  |  |  |  |
| --- | --- | --- | --- | --- | --- | --- | --- | --- | --- | --- | --- | --- | --- | --- | --- | --- | --- | --- |
| N=1,301,710 |  |  |  |  |  |  |  |  |  |  |  |  |  |  |  |  |  |  |
| R^2^=0.013 |  |  |  |  |  |  |  |  |  |  |  |  |  |  |  |  |  |  |
|  | **Small Town** | | | | | | **Micropolitan** | | | | | | **Metropolitan** | | | | | |
|  | **Coef** | **SE** | **Z Score** | **P-**  **value** | **LL CI** | **UL CI** | **Coef** | **SE** | **Z Score** | **P-**  **value** | **LL CI** | **UL CI** | **Coef** | **SE** | **Z Score** | **P-**  **value** | **LL CI** | **UL CI** |
| Frail | 0.030 | 0.015 | 1.990 | 0.047 | 0.000 | 0.060 | 0.059 | 0.015 | 3.900 | 0.000 | 0.029 | 0.089 | 0.163 | 0.013 | 12.390 | 0.000 | 0.137 | 0.189 |
| Mean age | -0.005 | 0.000 | -12.080 | 0.000 | -0.006 | -0.004 | -0.007 | 0.000 | -17.830 | 0.000 | -0.008 | -0.007 | -0.014 | 0.000 | -37.530 | 0.000 | -0.015 | -0.013 |
| Under 65 | 0.016 | 0.013 | 1.200 | 0.229 | -0.010 | 0.041 | -0.027 | 0.013 | -2.070 | 0.038 | -0.053 | -0.001 | -0.034 | 0.012 | -2.880 | 0.004 | -0.058 | -0.011 |
| Over 85 | -0.021 | 0.011 | -1.920 | 0.055 | -0.043 | 0.000 | -0.062 | 0.011 | -5.530 | 0.000 | -0.083 | -0.040 | -0.090 | 0.010 | -8.800 | 0.000 | -0.110 | -0.070 |
| Female | -0.014 | 0.005 | -2.670 | 0.008 | -0.024 | -0.004 | -0.024 | 0.005 | -4.750 | 0.000 | -0.034 | -0.014 | -0.087 | 0.005 | -18.980 | 0.000 | -0.097 | -0.078 |
| White vs. Unknown | 0.077 | 0.025 | 3.140 | 0.002 | 0.029 | 0.125 | 0.013 | 0.023 | 0.560 | 0.577 | -0.033 | 0.059 | -0.016 | 0.021 | -0.760 | 0.449 | -0.056 | 0.025 |
| Black vs. Unknown | 0.204 | 0.028 | 7.170 | 0.000 | 0.148 | 0.260 | 0.300 | 0.027 | 11.000 | 0.000 | 0.247 | 0.353 | 0.194 | 0.024 | 7.920 | 0.000 | 0.146 | 0.242 |
| Other vs. Unknown | 0.132 | 0.044 | 3.010 | 0.003 | 0.046 | 0.219 | 0.165 | 0.041 | 3.980 | 0.000 | 0.084 | 0.246 | 0.090 | 0.038 | 2.390 | 0.017 | 0.016 | 0.163 |
| Asian vs. Unknown | 0.159 | 0.071 | 2.230 | 0.026 | 0.019 | 0.298 | 0.408 | 0.063 | 6.430 | 0.000 | 0.283 | 0.532 | 0.242 | 0.059 | 4.090 | 0.000 | 0.126 | 0.358 |
| Hispanic vs. Unknown | 0.176 | 0.046 | 3.820 | 0.000 | 0.086 | 0.266 | 0.329 | 0.043 | 7.690 | 0.000 | 0.245 | 0.412 | 0.231 | 0.039 | 5.940 | 0.000 | 0.155 | 0.308 |
| North American Native vs. Unknown | -0.399 | 0.031 | -12.800 | 0.000 | -0.460 | -0.338 | -0.579 | 0.030 | -19.310 | 0.000 | -0.638 | -0.520 | -0.074 | 0.025 | -2.920 | 0.004 | -0.123 | -0.024 |
| Disabled (original reason for Medicare eligibility) | 0.081 | 0.009 | 9.180 | 0.000 | 0.064 | 0.098 | 0.029 | 0.009 | 3.220 | 0.001 | 0.011 | 0.046 | -0.049 | 0.008 | -5.970 | 0.000 | -0.065 | -0.033 |
| Dual eligible for Medicaid | -0.087 | 0.007 | -12.190 | 0.000 | -0.101 | -0.073 | -0.186 | 0.007 | -25.500 | 0.000 | -0.200 | -0.172 | -0.336 | 0.007 | -50.130 | 0.000 | -0.349 | -0.323 |
| Nursing home | 0.009 | 0.010 | 0.910 | 0.363 | -0.011 | 0.030 | -0.236 | 0.011 | -21.870 | 0.000 | -0.257 | -0.215 | -0.013 | 0.009 | -1.370 | 0.171 | -0.032 | 0.006 |
| Died in the year | 0.034 | 0.014 | 2.430 | 0.015 | 0.007 | 0.062 | 0.007 | 0.014 | 0.510 | 0.611 | -0.021 | 0.035 | 0.078 | 0.012 | 6.230 | 0.000 | 0.053 | 0.102 |
| Median household income (area) | 0.000 | 0.000 | 22.600 | 0.000 | 0.000 | 0.000 | 0.000 | 0.000 | 73.020 | 0.000 | 0.000 | 0.000 | 0.000 | 0.000 | 99.370 | 0.000 | 0.000 | 0.000 |
| Residents under poverty level | 0.003 | 0.000 | 7.060 | 0.000 | 0.002 | 0.004 | 0.018 | 0.000 | 37.800 | 0.000 | 0.017 | 0.019 | 0.011 | 0.000 | 25.170 | 0.000 | 0.010 | 0.012 |
| Number of hierarchical condition categories | 0.016 | 0.002 | 6.760 | 0.000 | 0.011 | 0.020 | 0.056 | 0.002 | 24.770 | 0.000 | 0.052 | 0.061 | 0.091 | 0.002 | 45.330 | 0.000 | 0.087 | 0.095 |
| Coronary artery disease | 0.019 | 0.013 | 1.470 | 0.141 | -0.006 | 0.045 | 0.059 | 0.013 | 4.650 | 0.000 | 0.034 | 0.084 | 0.032 | 0.011 | 2.790 | 0.005 | 0.009 | 0.055 |
| Congestive heart failure | -0.025 | 0.011 | -2.340 | 0.019 | -0.045 | -0.004 | -0.058 | 0.011 | -5.500 | 0.000 | -0.078 | -0.037 | -0.092 | 0.009 | -9.780 | 0.000 | -0.111 | -0.074 |
| Diabetes | 0.016 | 0.007 | 2.370 | 0.018 | 0.003 | 0.029 | -0.050 | 0.007 | -7.460 | 0.000 | -0.063 | -0.037 | -0.181 | 0.006 | -29.680 | 0.000 | -0.193 | -0.169 |
| Cancer | 0.020 | 0.010 | 1.950 | 0.052 | 0.000 | 0.040 | 0.106 | 0.010 | 10.820 | 0.000 | 0.087 | 0.125 | 0.199 | 0.009 | 23.120 | 0.000 | 0.182 | 0.216 |
| Chronic obstructive pulmonary disease | 0.018 | 0.009 | 2.040 | 0.041 | 0.001 | 0.036 | -0.086 | 0.009 | -9.540 | 0.000 | -0.103 | -0.068 | -0.250 | 0.008 | -30.280 | 0.000 | -0.266 | -0.234 |
| End stage renal disease | -0.009 | 0.032 | -0.290 | 0.770 | -0.073 | 0.054 | 0.052 | 0.031 | 1.690 | 0.090 | -0.008 | 0.112 | 0.256 | 0.026 | 9.820 | 0.000 | 0.205 | 0.307 |
| Midwest vs. Northeast | 0.146 | 0.008 | 18.460 | 0.000 | 0.131 | 0.162 | 0.269 | 0.008 | 33.970 | 0.000 | 0.254 | 0.285 | 0.159 | 0.007 | 21.900 | 0.000 | 0.145 | 0.174 |
| South vs. Northeast | 0.126 | 0.009 | 14.250 | 0.000 | 0.109 | 0.143 | 0.138 | 0.009 | 15.450 | 0.000 | 0.120 | 0.155 | 0.351 | 0.008 | 43.720 | 0.000 | 0.335 | 0.366 |
| West vs. Northeast | 0.122 | 0.010 | 12.580 | 0.000 | 0.103 | 0.141 | 0.449 | 0.009 | 48.020 | 0.000 | 0.430 | 0.467 | 0.743 | 0.008 | 89.690 | 0.000 | 0.726 | 0.759 |
| Constant | -0.983 | 0.043 | -22.780 | 0.000 | -1.068 | -0.899 | -1.719 | 0.042 | -40.880 | 0.000 | -1.801 | -1.636 | -1.036 | 0.038 | -27.530 | 0.000 | -1.110 | -0.962 |
| **Isolated Beneficiaries Travel to:** | **Margin** | **SE** | **Z Score** | **P-value** | **LL CI** | **UL CI** |  |  |  |  |  |  |  |  |  |  |  |  |
| Isolated Practice | 0.375 | 0.000 | 890.750 | 0.000 | 0.374 | 0.375 |  |  |  |  |  |  |  |  |  |  |  |  |
| Small Town Practice | 0.178 | 0.000 | 532.230 | 0.000 | 0.178 | 0.179 |  |  |  |  |  |  |  |  |  |  |  |  |
| Micropolitan Practice | 0.186 | 0.000 | 545.730 | 0.000 | 0.185 | 0.186 |  |  |  |  |  |  |  |  |  |  |  |  |
| Metropolitan Practice | 0.262 | 0.000 | 687.320 | 0.000 | 0.261 | 0.262 |  |  |  |  |  |  |  |  |  |  |  |  |

| **Practices where small town beneficiaries get care** |  |  |  |  |  |  |  |  |  |  |  |  |  |  |  |  |  |  |
| --- | --- | --- | --- | --- | --- | --- | --- | --- | --- | --- | --- | --- | --- | --- | --- | --- | --- | --- |
| N=1,887,823 |  |  |  |  |  |  |  |  |  |  |  |  |  |  |  |  |  |  |
| R^2^=0.013 |  |  |  |  |  |  |  |  |  |  |  |  |  |  |  |  |  |  |
|  | **Small Town** | | | | | | **Micropolitan** | | | | | | **Metropolitan** | | | | | |
|  | **Coef** | **SE** | **Z Score** | **P-**  **value** | **LL CI** | **UL CI** | **Coef** | **SE** | **Z Score** | **P-**  **value** | **LL CI** | **UL CI** | **Coef** | **SE** | **Z Score** | **P-**  **value** | **LL CI** | **UL CI** |
| Frail | 0.013 | 0.019 | 0.680 | 0.496 | -0.025 | 0.051 | 0.069 | 0.013 | 5.420 | 0.000 | 0.044 | 0.094 | 0.150 | 0.010 | 15.750 | 0.000 | 0.132 | 0.169 |
| Mean age | -0.004 | 0.001 | -8.350 | 0.000 | -0.006 | -0.003 | -0.009 | 0.000 | -25.760 | 0.000 | -0.010 | -0.008 | -0.013 | 0.000 | -47.040 | 0.000 | -0.014 | -0.013 |
| Under 65 | 0.004 | 0.017 | 0.250 | 0.800 | -0.029 | 0.037 | -0.023 | 0.011 | -2.100 | 0.036 | -0.045 | -0.002 | -0.057 | 0.009 | -6.460 | 0.000 | -0.075 | -0.040 |
| Over 85 | -0.064 | 0.015 | -4.290 | 0.000 | -0.093 | -0.035 | -0.115 | 0.010 | -11.340 | 0.000 | -0.134 | -0.095 | -0.124 | 0.008 | -15.840 | 0.000 | -0.140 | -0.109 |
| Female | -0.056 | 0.007 | -8.030 | 0.000 | -0.070 | -0.043 | -0.054 | 0.005 | -11.790 | 0.000 | -0.063 | -0.045 | -0.105 | 0.004 | -29.510 | 0.000 | -0.112 | -0.098 |
| White vs. Unknown | 0.003 | 0.035 | 0.070 | 0.940 | -0.065 | 0.070 | 0.031 | 0.023 | 1.370 | 0.171 | -0.013 | 0.076 | -0.083 | 0.017 | -4.880 | 0.000 | -0.116 | -0.050 |
| Black vs. Unknown | -0.324 | 0.039 | -8.400 | 0.000 | -0.399 | -0.248 | -0.027 | 0.025 | -1.070 | 0.283 | -0.075 | 0.022 | 0.019 | 0.018 | 1.000 | 0.316 | -0.018 | 0.055 |
| Other vs. Unknown | -0.153 | 0.061 | -2.510 | 0.012 | -0.272 | -0.034 | 0.048 | 0.037 | 1.280 | 0.200 | -0.025 | 0.121 | -0.142 | 0.029 | -4.890 | 0.000 | -0.199 | -0.085 |
| Asian vs. Unknown | -0.363 | 0.096 | -3.800 | 0.000 | -0.550 | -0.176 | 0.143 | 0.051 | 2.780 | 0.005 | 0.042 | 0.244 | -0.026 | 0.040 | -0.650 | 0.514 | -0.105 | 0.053 |
| Hispanic vs. Unknown | -0.193 | 0.057 | -3.370 | 0.001 | -0.305 | -0.081 | 0.135 | 0.034 | 3.950 | 0.000 | 0.068 | 0.202 | 0.182 | 0.026 | 7.130 | 0.000 | 0.132 | 0.233 |
| North American Native vs. Unknown | 0.692 | 0.042 | 16.400 | 0.000 | 0.609 | 0.775 | 0.240 | 0.029 | 8.140 | 0.000 | 0.182 | 0.297 | 0.284 | 0.022 | 12.850 | 0.000 | 0.241 | 0.328 |
| Disabled (original reason for Medicare eligibility) | 0.125 | 0.012 | 10.820 | 0.000 | 0.102 | 0.147 | 0.030 | 0.008 | 3.880 | 0.000 | 0.015 | 0.045 | -0.053 | 0.006 | -8.640 | 0.000 | -0.065 | -0.041 |
| Dual eligible for Medicaid | 0.142 | 0.009 | 15.370 | 0.000 | 0.124 | 0.161 | -0.091 | 0.006 | -14.380 | 0.000 | -0.103 | -0.079 | -0.267 | 0.005 | -52.540 | 0.000 | -0.277 | -0.257 |
| Nursing home | 0.248 | 0.013 | 18.790 | 0.000 | 0.222 | 0.273 | -0.007 | 0.009 | -0.770 | 0.443 | -0.025 | 0.011 | 0.075 | 0.007 | 10.550 | 0.000 | 0.061 | 0.089 |
| Died in the year | 0.094 | 0.018 | 5.180 | 0.000 | 0.058 | 0.129 | 0.043 | 0.012 | 3.480 | 0.000 | 0.019 | 0.067 | 0.075 | 0.009 | 8.090 | 0.000 | 0.057 | 0.094 |
| Median household income (area) | 0.000 | 0.000 | -1.240 | 0.214 | 0.000 | 0.000 | 0.000 | 0.000 | 30.730 | 0.000 | 0.000 | 0.000 | 0.000 | 0.000 | 86.960 | 0.000 | 0.000 | 0.000 |
| Residents under poverty level | 0.001 | 0.001 | 0.960 | 0.339 | -0.001 | 0.002 | 0.007 | 0.000 | 18.380 | 0.000 | 0.006 | 0.007 | 0.001 | 0.000 | 4.910 | 0.000 | 0.001 | 0.002 |
| Number of hierarchical condition categories | -0.018 | 0.003 | -5.970 | 0.000 | -0.024 | -0.012 | 0.044 | 0.002 | 22.740 | 0.000 | 0.040 | 0.048 | 0.077 | 0.001 | 52.280 | 0.000 | 0.074 | 0.080 |
| Coronary artery disease | 0.025 | 0.017 | 1.450 | 0.147 | -0.009 | 0.059 | 0.034 | 0.011 | 3.100 | 0.002 | 0.012 | 0.055 | 0.000 | 0.008 | -0.040 | 0.964 | -0.017 | 0.016 |
| Congestive heart failure | -0.007 | 0.014 | -0.490 | 0.626 | -0.034 | 0.021 | -0.065 | 0.009 | -7.150 | 0.000 | -0.083 | -0.048 | -0.073 | 0.007 | -10.280 | 0.000 | -0.087 | -0.059 |
| Diabetes | 0.037 | 0.009 | 4.190 | 0.000 | 0.020 | 0.055 | -0.055 | 0.006 | -9.510 | 0.000 | -0.067 | -0.044 | -0.189 | 0.005 | -41.430 | 0.000 | -0.198 | -0.180 |
| Cancer | -0.074 | 0.014 | -5.190 | 0.000 | -0.102 | -0.046 | 0.075 | 0.009 | 8.740 | 0.000 | 0.059 | 0.092 | 0.189 | 0.006 | 29.150 | 0.000 | 0.177 | 0.202 |
| Chronic obstructive pulmonary disease | 0.037 | 0.012 | 3.180 | 0.001 | 0.014 | 0.060 | -0.072 | 0.008 | -9.390 | 0.000 | -0.088 | -0.057 | -0.250 | 0.006 | -40.480 | 0.000 | -0.263 | -0.238 |
| End stage renal disease | -0.069 | 0.040 | -1.730 | 0.083 | -0.148 | 0.009 | 0.118 | 0.023 | 5.110 | 0.000 | 0.072 | 0.163 | 0.215 | 0.017 | 12.320 | 0.000 | 0.181 | 0.249 |
| Midwest vs. Northeast | -0.647 | 0.010 | -62.430 | 0.000 | -0.668 | -0.627 | -0.060 | 0.008 | -7.160 | 0.000 | -0.076 | -0.043 | 0.270 | 0.007 | 36.990 | 0.000 | 0.256 | 0.284 |
| South vs. Northeast | -0.976 | 0.011 | -88.930 | 0.000 | -0.998 | -0.955 | -0.221 | 0.009 | -25.930 | 0.000 | -0.238 | -0.205 | 0.360 | 0.007 | 48.860 | 0.000 | 0.345 | 0.374 |
| West vs. Northeast | -1.127 | 0.014 | -81.280 | 0.000 | -1.154 | -1.100 | -0.186 | 0.010 | -19.400 | 0.000 | -0.205 | -0.167 | 0.330 | 0.008 | 41.150 | 0.000 | 0.314 | 0.345 |
| Constant | -1.383 | 0.056 | -24.560 | 0.000 | -1.493 | -1.272 | -1.152 | 0.037 | -30.750 | 0.000 | -1.225 | -1.079 | -0.768 | 0.029 | -26.480 | 0.000 | -0.825 | -0.711 |
|  |  |  |  |  |  |  |  |  |  |  |  |  |  |  |  |  |  |  |
| **Small Town Beneficiaries Travel to:** | **Margin** | **SE** | **Z Score** | **P-value** | **LL CI** | **UL CI** |  |  |  |  |  |  |  |  |  |  |  |  |
| Isolated Practice | 0.049 | 0.000 | 314.590 | 0.000 | 0.049 | 0.050 |  |  |  |  |  |  |  |  |  |  |  |  |
| Small Town Practice | 0.559 | 0.000 | 1558.230 | 0.000 | 0.559 | 0.560 |  |  |  |  |  |  |  |  |  |  |  |  |
| Micropolitan Practice | 0.131 | 0.000 | 533.550 | 0.000 | 0.130 | 0.131 |  |  |  |  |  |  |  |  |  |  |  |  |
| Metropolitan Practice | 0.260 | 0.000 | 822.320 | 0.000 | 0.260 | 0.261 |  |  |  |  |  |  |  |  |  |  |  |  |

| **Practices where micropolitan beneficiaries get care** |  |  |  |  |  |  |  |  |  |  |  |  |  |  |  |  |  |  |
| --- | --- | --- | --- | --- | --- | --- | --- | --- | --- | --- | --- | --- | --- | --- | --- | --- | --- | --- |
| N=3,464,907 |  |  |  |  |  |  |  |  |  |  |  |  |  |  |  |  |  |  |
| R^2^=0.016 |  |  |  |  |  |  |  |  |  |  |  |  |  |  |  |  |  |  |
|  | **Small Town** | | | | | | **Micropolitan** | | | | | | **Metropolitan** | | | | | |
|  | **Coef** | **SE** | **Z Score** | **P-**  **value** | **LL CI** | **UL CI** | **Coef** | **SE** | **Z Score** | **P-**  **value** | **LL CI** | **UL CI** | **Coef** | **SE** | **Z Score** | **P-**  **value** | **LL CI** | **UL CI** |
| Frail | -0.106 | 0.021 | -5.000 | 0.000 | -0.147 | -0.064 | 0.002 | 0.015 | 0.120 | 0.904 | -0.027 | 0.031 | 0.146 | 0.007 | 21.080 | 0.000 | 0.132 | 0.159 |
| Mean age | -0.003 | 0.001 | -4.940 | 0.000 | -0.004 | -0.002 | -0.006 | 0.000 | -15.630 | 0.000 | -0.007 | -0.006 | -0.013 | 0.000 | -64.270 | 0.000 | -0.014 | -0.013 |
| Under 65 | -0.002 | 0.017 | -0.090 | 0.927 | -0.036 | 0.032 | 0.012 | 0.013 | 0.940 | 0.345 | -0.013 | 0.037 | -0.014 | 0.007 | -2.110 | 0.035 | -0.027 | -0.001 |
| Over 85 | -0.088 | 0.016 | -5.430 | 0.000 | -0.119 | -0.056 | -0.080 | 0.012 | -6.540 | 0.000 | -0.103 | -0.056 | -0.068 | 0.006 | -11.260 | 0.000 | -0.080 | -0.056 |
| Female | -0.078 | 0.007 | -10.500 | 0.000 | -0.093 | -0.064 | -0.086 | 0.005 | -15.680 | 0.000 | -0.097 | -0.075 | -0.106 | 0.003 | -39.240 | 0.000 | -0.111 | -0.101 |
| White vs. Unknown | 0.027 | 0.037 | 0.720 | 0.469 | -0.046 | 0.100 | 0.095 | 0.029 | 3.330 | 0.001 | 0.039 | 0.151 | -0.088 | 0.012 | -7.110 | 0.000 | -0.112 | -0.064 |
| Black vs. Unknown | -0.176 | 0.041 | -4.320 | 0.000 | -0.256 | -0.096 | -0.015 | 0.031 | -0.480 | 0.631 | -0.074 | 0.045 | -0.107 | 0.014 | -7.870 | 0.000 | -0.133 | -0.080 |
| Other vs. Unknown | -0.422 | 0.067 | -6.330 | 0.000 | -0.552 | -0.291 | 0.023 | 0.044 | 0.520 | 0.601 | -0.063 | 0.109 | -0.115 | 0.019 | -5.970 | 0.000 | -0.152 | -0.077 |
| Asian vs. Unknown | -0.709 | 0.096 | -7.430 | 0.000 | -0.897 | -0.522 | -0.098 | 0.057 | -1.720 | 0.085 | -0.211 | 0.014 | -0.011 | 0.023 | -0.470 | 0.641 | -0.057 | 0.035 |
| Hispanic vs. Unknown | -0.195 | 0.055 | -3.540 | 0.000 | -0.303 | -0.087 | -0.285 | 0.042 | -6.770 | 0.000 | -0.368 | -0.203 | 0.129 | 0.017 | 7.550 | 0.000 | 0.095 | 0.162 |
| North American Native vs. Unknown | 2.160 | 0.041 | 53.000 | 0.000 | 2.080 | 2.239 | 0.898 | 0.035 | 25.460 | 0.000 | 0.829 | 0.968 | 0.240 | 0.018 | 13.590 | 0.000 | 0.205 | 0.274 |
| Disabled (original reason for Medicare eligibility) | 0.183 | 0.012 | 15.000 | 0.000 | 0.159 | 0.207 | 0.173 | 0.009 | 19.250 | 0.000 | 0.155 | 0.190 | 0.016 | 0.005 | 3.370 | 0.001 | 0.007 | 0.025 |
| Dual eligible for Medicaid | 0.353 | 0.010 | 36.140 | 0.000 | 0.334 | 0.372 | 0.215 | 0.007 | 29.470 | 0.000 | 0.201 | 0.229 | -0.153 | 0.004 | -39.350 | 0.000 | -0.160 | -0.145 |
| Nursing home | 0.462 | 0.014 | 32.980 | 0.000 | 0.434 | 0.489 | 0.405 | 0.011 | 38.340 | 0.000 | 0.384 | 0.425 | 0.477 | 0.005 | 89.260 | 0.000 | 0.467 | 0.488 |
| Died in the year | 0.134 | 0.019 | 6.990 | 0.000 | 0.096 | 0.171 | 0.144 | 0.014 | 10.150 | 0.000 | 0.116 | 0.172 | 0.139 | 0.007 | 20.160 | 0.000 | 0.126 | 0.153 |
| Median household income (area) | 0.000 | 0.000 | -15.620 | 0.000 | 0.000 | 0.000 | 0.000 | 0.000 | -34.270 | 0.000 | 0.000 | 0.000 | 0.000 | 0.000 | 57.740 | 0.000 | 0.000 | 0.000 |
| Residents under poverty level | -0.007 | 0.001 | -12.630 | 0.000 | -0.008 | -0.006 | -0.003 | 0.000 | -7.720 | 0.000 | -0.004 | -0.002 | 0.006 | 0.000 | 30.870 | 0.000 | 0.006 | 0.007 |
| Number of hierarchical condition categories | -0.059 | 0.003 | -18.700 | 0.000 | -0.065 | -0.053 | -0.037 | 0.002 | -16.180 | 0.000 | -0.041 | -0.032 | 0.044 | 0.001 | 40.760 | 0.000 | 0.042 | 0.046 |
| Coronary artery disease | 0.014 | 0.018 | 0.770 | 0.439 | -0.022 | 0.050 | 0.007 | 0.013 | 0.560 | 0.577 | -0.019 | 0.033 | -0.023 | 0.006 | -3.630 | 0.000 | -0.036 | -0.011 |
| Congestive heart failure | 0.043 | 0.015 | 2.880 | 0.004 | 0.014 | 0.073 | 0.045 | 0.011 | 4.150 | 0.000 | 0.024 | 0.066 | -0.053 | 0.005 | -9.860 | 0.000 | -0.063 | -0.042 |
| Diabetes | 0.051 | 0.009 | 5.380 | 0.000 | 0.032 | 0.069 | 0.052 | 0.007 | 7.590 | 0.000 | 0.039 | 0.066 | -0.160 | 0.003 | -46.210 | 0.000 | -0.167 | -0.153 |
| Cancer | -0.038 | 0.015 | -2.570 | 0.010 | -0.067 | -0.009 | -0.055 | 0.011 | -5.030 | 0.000 | -0.076 | -0.033 | 0.137 | 0.005 | 28.330 | 0.000 | 0.128 | 0.147 |
| Chronic obstructive pulmonary disease | 0.074 | 0.012 | 6.030 | 0.000 | 0.050 | 0.098 | 0.084 | 0.009 | 9.390 | 0.000 | 0.067 | 0.102 | -0.217 | 0.005 | -46.030 | 0.000 | -0.226 | -0.208 |
| End stage renal disease | -0.040 | 0.039 | -1.020 | 0.308 | -0.116 | 0.037 | -0.068 | 0.028 | -2.430 | 0.015 | -0.123 | -0.013 | 0.161 | 0.012 | 12.960 | 0.000 | 0.136 | 0.185 |
| Midwest vs. Northeast | -0.396 | 0.011 | -35.530 | 0.000 | -0.418 | -0.374 | -0.117 | 0.010 | -11.950 | 0.000 | -0.136 | -0.097 | -0.307 | 0.005 | -62.140 | 0.000 | -0.317 | -0.298 |
| South vs. Northeast | -0.677 | 0.011 | -60.090 | 0.000 | -0.699 | -0.655 | -0.008 | 0.009 | -0.820 | 0.412 | -0.026 | 0.011 | -0.031 | 0.005 | -6.620 | 0.000 | -0.040 | -0.022 |
| West vs. Northeast | -1.142 | 0.015 | -77.760 | 0.000 | -1.171 | -1.113 | -0.616 | 0.012 | -52.240 | 0.000 | -0.639 | -0.593 | -0.002 | 0.005 | -0.460 | 0.643 | -0.013 | 0.008 |
| Constant | -2.436 | 0.059 | -41.320 | 0.000 | -2.552 | -2.321 | -1.886 | 0.045 | -42.200 | 0.000 | -1.974 | -1.799 | -0.524 | 0.021 | -24.840 | 0.000 | -0.565 | -0.483 |
|  |  |  |  |  |  |  |  |  |  |  |  |  |  |  |  |  |  |  |
| **Micropolitan Beneficiaries Travel to:** | **Margin** | **SE** | **Z Score** | **P-**  **value** | **LL CI** | **UL CI** |  |  |  |  |  |  |  |  |  |  |  |  |
| Isolated Practice | 0.023 | 0.000 | 284.510 | 0.000 | 0.022 | 0.023 |  |  |  |  |  |  |  |  |  |  |  |  |
| Small Town Practice | 0.043 | 0.000 | 393.270 | 0.000 | 0.042 | 0.043 |  |  |  |  |  |  |  |  |  |  |  |  |
| Micropolitan Practice | 0.719 | 0.000 | 2995.620 | 0.000 | 0.718 | 0.719 |  |  |  |  |  |  |  |  |  |  |  |  |
| Metropolitan Practice | 0.216 | 0.000 | 984.310 | 0.000 | 0.216 | 0.217 |  |  |  |  |  |  |  |  |  |  |  |  |

| **Practices where metropolitan beneficiaries get care** |  |  |  |  |  |  |  |  |  |  |  |  |  |  |  |  |  |  |
| --- | --- | --- | --- | --- | --- | --- | --- | --- | --- | --- | --- | --- | --- | --- | --- | --- | --- | --- |
| N=21,038,399 |  |  |  |  |  |  |  |  |  |  |  |  |  |  |  |  |  |  |
| R^2^=0.038 |  |  |  |  |  |  |  |  |  |  |  |  |  |  |  |  |  |  |
|  | **Small Town** | | | | | | **Micropolitan** | | | | | | **Metropolitan** | | | | | |
|  | **Coef** | **SE** | **Z Score** | **P-**  **value** | **LL CI** | **UL CI** | **Coef** | **SE** | **Z Score** | **P-**  **value** | **LL CI** | **UL CI** | **Coef** | **SE** | **Z Score** | **P-**  **value** | **LL CI** | **UL CI** |
| Frail | -0.337 | 0.018 | -18.590 | 0.000 | -0.372 | -0.301 | -0.187 | 0.011 | -17.460 | 0.000 | -0.208 | -0.166 | -0.094 | 0.008 | -12.000 | 0.000 | -0.110 | -0.079 |
| Mean age | 0.002 | 0.001 | 4.480 | 0.000 | 0.001 | 0.003 | 0.001 | 0.000 | 1.580 | 0.113 | 0.000 | 0.001 | -0.005 | 0.000 | -19.730 | 0.000 | -0.005 | -0.004 |
| Under 65 | -0.008 | 0.017 | -0.490 | 0.624 | -0.042 | 0.025 | -0.015 | 0.010 | -1.440 | 0.150 | -0.036 | 0.005 | 0.032 | 0.008 | 4.030 | 0.000 | 0.017 | 0.048 |
| Over 85 | -0.030 | 0.015 | -2.070 | 0.038 | -0.059 | -0.002 | -0.019 | 0.009 | -2.090 | 0.037 | -0.037 | -0.001 | 0.014 | 0.007 | 2.070 | 0.039 | 0.001 | 0.028 |
| Female | -0.132 | 0.007 | -18.600 | 0.000 | -0.146 | -0.118 | -0.095 | 0.004 | -21.850 | 0.000 | -0.103 | -0.086 | -0.093 | 0.003 | -28.570 | 0.000 | -0.099 | -0.086 |
| White vs. Unknown | 0.319 | 0.032 | 9.820 | 0.000 | 0.255 | 0.383 | 0.437 | 0.022 | 20.300 | 0.000 | 0.394 | 0.479 | 0.334 | 0.015 | 22.350 | 0.000 | 0.305 | 0.363 |
| Black vs. Unknown | -0.804 | 0.036 | -22.250 | 0.000 | -0.875 | -0.734 | -0.318 | 0.023 | -13.900 | 0.000 | -0.362 | -0.273 | -0.303 | 0.016 | -18.940 | 0.000 | -0.335 | -0.272 |
| Other vs. Unknown | -0.582 | 0.054 | -10.860 | 0.000 | -0.687 | -0.477 | -0.552 | 0.035 | -15.590 | 0.000 | -0.621 | -0.483 | -0.271 | 0.023 | -12.020 | 0.000 | -0.315 | -0.227 |
| Asian vs. Unknown | -1.563 | 0.069 | -22.790 | 0.000 | -1.697 | -1.428 | -1.164 | 0.040 | -29.350 | 0.000 | -1.241 | -1.086 | -0.780 | 0.024 | -32.100 | 0.000 | -0.827 | -0.732 |
| Hispanic vs. Unknown | -1.042 | 0.051 | -20.250 | 0.000 | -1.143 | -0.941 | -0.821 | 0.032 | -25.880 | 0.000 | -0.884 | -0.759 | -0.427 | 0.020 | -20.980 | 0.000 | -0.467 | -0.387 |
| North American Native vs. Unknown | 2.685 | 0.038 | 71.250 | 0.000 | 2.612 | 2.759 | 2.190 | 0.027 | 82.570 | 0.000 | 2.138 | 2.242 | 1.628 | 0.021 | 77.660 | 0.000 | 1.587 | 1.669 |
| Disabled (original reason for Medicare eligibility) | 0.307 | 0.012 | 25.440 | 0.000 | 0.283 | 0.331 | 0.311 | 0.007 | 42.290 | 0.000 | 0.296 | 0.325 | 0.257 | 0.006 | 45.120 | 0.000 | 0.245 | 0.268 |
| Dual eligible for Medicaid | 0.416 | 0.010 | 43.350 | 0.000 | 0.397 | 0.435 | 0.396 | 0.006 | 67.040 | 0.000 | 0.384 | 0.408 | 0.273 | 0.005 | 60.460 | 0.000 | 0.265 | 0.282 |
| Nursing home | 0.712 | 0.012 | 58.160 | 0.000 | 0.688 | 0.736 | 0.539 | 0.008 | 69.450 | 0.000 | 0.524 | 0.554 | 0.460 | 0.006 | 76.780 | 0.000 | 0.448 | 0.471 |
| Died in the year | 0.201 | 0.017 | 11.760 | 0.000 | 0.168 | 0.235 | 0.231 | 0.011 | 21.900 | 0.000 | 0.211 | 0.252 | 0.242 | 0.008 | 30.260 | 0.000 | 0.226 | 0.257 |
| Median household income (area) | 0.000 | 0.000 | -93.850 | 0.000 | 0.000 | 0.000 | 0.000 | 0.000 | -156.750 | 0.000 | 0.000 | 0.000 | 0.000 | 0.000 | -162.440 | 0.000 | 0.000 | 0.000 |
| Residents under poverty level | -0.015 | 0.000 | -31.610 | 0.000 | -0.016 | -0.014 | -0.013 | 0.000 | -45.650 | 0.000 | -0.014 | -0.013 | -0.014 | 0.000 | -61.160 | 0.000 | -0.014 | -0.013 |
| Number of hierarchical condition categories | -0.102 | 0.003 | -35.260 | 0.000 | -0.108 | -0.096 | -0.092 | 0.002 | -52.610 | 0.000 | -0.096 | -0.089 | -0.052 | 0.001 | -40.990 | 0.000 | -0.055 | -0.050 |
| Coronary artery disease | 0.036 | 0.018 | 2.050 | 0.041 | 0.002 | 0.070 | 0.079 | 0.010 | 7.550 | 0.000 | 0.058 | 0.099 | 0.043 | 0.008 | 5.560 | 0.000 | 0.028 | 0.059 |
| Congestive heart failure | 0.105 | 0.014 | 7.520 | 0.000 | 0.078 | 0.132 | 0.133 | 0.008 | 15.780 | 0.000 | 0.116 | 0.149 | 0.077 | 0.006 | 12.130 | 0.000 | 0.065 | 0.090 |
| Diabetes | 0.103 | 0.009 | 11.310 | 0.000 | 0.085 | 0.121 | 0.140 | 0.005 | 25.650 | 0.000 | 0.130 | 0.151 | 0.053 | 0.004 | 12.710 | 0.000 | 0.045 | 0.061 |
| Cancer | -0.176 | 0.014 | -12.540 | 0.000 | -0.203 | -0.148 | -0.136 | 0.008 | -16.160 | 0.000 | -0.152 | -0.119 | -0.129 | 0.006 | -21.030 | 0.000 | -0.141 | -0.117 |
| Chronic obstructive pulmonary disease | 0.246 | 0.012 | 20.790 | 0.000 | 0.223 | 0.270 | 0.205 | 0.007 | 28.310 | 0.000 | 0.191 | 0.220 | 0.138 | 0.006 | 24.890 | 0.000 | 0.127 | 0.149 |
| End stage renal disease | -0.157 | 0.038 | -4.170 | 0.000 | -0.231 | -0.083 | -0.150 | 0.022 | -6.920 | 0.000 | -0.192 | -0.107 | -0.138 | 0.015 | -8.990 | 0.000 | -0.168 | -0.108 |
| Midwest vs. Northeast | 0.064 | 0.010 | 6.120 | 0.000 | 0.043 | 0.084 | 0.856 | 0.008 | 110.680 | 0.000 | 0.841 | 0.871 | 0.284 | 0.005 | 52.070 | 0.000 | 0.274 | 0.295 |
| South vs. Northeast | -0.107 | 0.010 | -11.070 | 0.000 | -0.126 | -0.088 | 0.753 | 0.007 | 102.550 | 0.000 | 0.739 | 0.768 | 0.501 | 0.005 | 103.330 | 0.000 | 0.491 | 0.510 |
| West vs. Northeast | -0.447 | 0.012 | -35.860 | 0.000 | -0.471 | -0.422 | 0.156 | 0.009 | 17.210 | 0.000 | 0.139 | 0.174 | 0.064 | 0.006 | 10.770 | 0.000 | 0.053 | 0.076 |
| Constant | -4.362 | 0.053 | -82.360 | 0.000 | -4.466 | -4.259 | -4.012 | 0.034 | -119.210 | 0.000 | -4.078 | -3.946 | -3.050 | 0.024 | -126.790 | 0.000 | -3.097 | -3.002 |
|  |  |  |  |  |  |  |  |  |  |  |  |  |  |  |  |  |  |  |
| **Micropolitan Beneficiaries Travel to:** | **Margin** | **SE** | **Z Score** | **P-**  **value** | **LL CI** | **UL CI** |  |  |  |  |  |  |  |  |  |  |  |  |
| Isolated Practice | 0.004 | 0.000 | 289.200 | 0.000 | 0.004 | 0.004 |  |  |  |  |  |  |  |  |  |  |  |  |
| Small Town Practice | 0.011 | 0.000 | 479.440 | 0.000 | 0.011 | 0.011 |  |  |  |  |  |  |  |  |  |  |  |  |
| Micropolitan Practice | 0.019 | 0.000 | 643.860 | 0.000 | 0.019 | 0.019 |  |  |  |  |  |  |  |  |  |  |  |  |
| Metropolitan Practice | 0.966 | 0.000 | 25000.000 | 0.000 | 0.966 | 0.966 |  |  |  |  |  |  |  |  |  |  |  |  |

| **Distance from beneficiary to practice** |  |  |  |  |  |  |
| --- | --- | --- | --- | --- | --- | --- |
| N | 26,577,788 |  |  |  |  |  |
| R^2^ | 0.029 |  |  |  |  |  |
|  | **Coefficient** | **SE** | **Z Score** | **P-value** | **LL CI** | **UL CI** |
| Small Town vs. Isolated | -5.546 | 0.042 | -132.830 | 0.000 | -5.628 | -5.464 |
| Micropolitan vs. Isolated | -9.733 | 0.038 | -257.450 | 0.000 | -9.807 | -9.659 |
| Metropolitan vs. Isolated | -15.224 | 0.034 | -450.480 | 0.000 | -15.290 | -15.157 |
| Frail | 1.153 | 0.035 | 32.970 | 0.000 | 1.085 | 1.222 |
| Mean age | -0.114 | 0.001 | -101.180 | 0.000 | -0.116 | -0.112 |
| Under 65 | 0.148 | 0.038 | 3.890 | 0.000 | 0.073 | 0.222 |
| Over 85 | 0.941 | 0.030 | 31.320 | 0.000 | 0.882 | 1.000 |
| Female | -0.911 | 0.014 | -63.920 | 0.000 | -0.939 | -0.883 |
| White vs. Unknown | 0.579 | 0.058 | 10.030 | 0.000 | 0.466 | 0.692 |
| Black vs. Unknown | -0.191 | 0.062 | -3.060 | 0.002 | -0.314 | -0.069 |
| Other vs. Unknown | -0.489 | 0.082 | -5.930 | 0.000 | -0.651 | -0.328 |
| Asian vs. Unknown | -1.768 | 0.080 | -22.200 | 0.000 | -1.924 | -1.612 |
| Hispanic vs. Unknown | -0.698 | 0.078 | -8.890 | 0.000 | -0.852 | -0.544 |
| North American Native vs. Unknown | 12.915 | 0.112 | 114.970 | 0.000 | 12.695 | 13.135 |
| Disabled (original reason for Medicare eligibility) | 0.843 | 0.027 | 31.780 | 0.000 | 0.791 | 0.895 |
| Dual eligible for Medicaid | 0.545 | 0.021 | 25.850 | 0.000 | 0.504 | 0.586 |
| Nursing home | 11.092 | 0.029 | 377.510 | 0.000 | 11.035 | 11.150 |
| Died in the year | 3.457 | 0.039 | 88.440 | 0.000 | 3.380 | 3.533 |
| Median household income (area) | 0.000 | 0.000 | -30.930 | 0.000 | 0.000 | 0.000 |
| Residents under poverty level | -0.043 | 0.001 | -43.980 | 0.000 | -0.044 | -0.041 |
| Number of hierarchical condition categories | 0.142 | 0.006 | 24.630 | 0.000 | 0.131 | 0.153 |
| Coronary artery disease | -0.735 | 0.035 | -21.190 | 0.000 | -0.802 | -0.667 |
| Congestive heart failure | -0.468 | 0.028 | -16.460 | 0.000 | -0.524 | -0.412 |
| Diabetes | -1.536 | 0.018 | -84.150 | 0.000 | -1.572 | -1.500 |
| Cancer | 0.481 | 0.025 | 19.100 | 0.000 | 0.432 | 0.530 |
| Chronic obstructive pulmonary disease | -1.426 | 0.026 | -55.810 | 0.000 | -1.476 | -1.376 |
| End stage renal disease | 0.710 | 0.066 | 10.700 | 0.000 | 0.580 | 0.841 |
| Midwest vs. Northeast | 1.958 | 0.022 | 88.600 | 0.000 | 1.915 | 2.001 |
| South vs. Northeast | 4.994 | 0.020 | 248.510 | 0.000 | 4.954 | 5.033 |
| West vs. Northeast | 6.489 | 0.023 | 277.060 | 0.000 | 6.443 | 6.535 |
| Constant | 33.048 | 0.106 | 311.180 | 0.000 | 32.840 | 33.256 |
| **Overall Distance** | **Margin** | **SE** | **Z Score** | **P-value** | **LL CI** | **UL CI** |
| Isolated | 28.536 | 0.033 | 876.290 | 0.000 | 28.472 | 28.600 |
| Small Town | 22.990 | 0.027 | 846.830 | 0.000 | 22.937 | 23.044 |
| Micropolitan | 18.803 | 0.020 | 927.260 | 0.000 | 18.763 | 18.843 |
| Metropolitan | 13.313 | 0.008 | 1637.470 | 0.000 | 13.297 | 13.328 |

| **Distance from isolated beneficiaries to practice** |  |  |  |  |  |  |
| --- | --- | --- | --- | --- | --- | --- |
| N | 1,251,001 |  |  |  |  |  |
| R^2^ | 0.214 |  |  |  |  |  |
|  | **Coefficient** | **SE** | **Z Score** | **P-value** | **LL CI** | **UL CI** |
| Small Town vs. Isolated | 13.405 | 0.102 | 130.930 | 0.000 | 13.205 | 13.606 |
| Micropolitan vs. Isolated | 21.515 | 0.102 | 211.490 | 0.000 | 21.316 | 21.715 |
| Metropolitan vs. Isolated | 51.354 | 0.094 | 546.580 | 0.000 | 51.170 | 51.538 |
| Frail | 1.060 | 0.212 | 5.000 | 0.000 | 0.645 | 1.476 |
| Mean age | -0.058 | 0.006 | -9.910 | 0.000 | -0.070 | -0.047 |
| Under 65 | 0.150 | 0.187 | 0.800 | 0.423 | -0.216 | 0.515 |
| Over 85 | 0.985 | 0.159 | 6.190 | 0.000 | 0.673 | 1.297 |
| Female | -0.852 | 0.073 | -11.630 | 0.000 | -0.995 | -0.708 |
| White vs. Unknown | -0.141 | 0.335 | -0.420 | 0.674 | -0.798 | 0.516 |
| Black vs. Unknown | -2.302 | 0.392 | -5.870 | 0.000 | -3.071 | -1.533 |
| Other vs. Unknown | 0.103 | 0.626 | 0.170 | 0.869 | -1.123 | 1.330 |
| Asian vs. Unknown | 1.873 | 1.051 | 1.780 | 0.075 | -0.188 | 3.934 |
| Hispanic vs. Unknown | -0.459 | 0.619 | -0.740 | 0.458 | -1.672 | 0.754 |
| North American Native vs. Unknown | 12.753 | 0.430 | 29.680 | 0.000 | 11.911 | 13.595 |
| Disabled (original reason for Medicare eligibility) | -0.608 | 0.127 | -4.770 | 0.000 | -0.858 | -0.358 |
| Dual eligible for Medicaid | -0.217 | 0.104 | -2.080 | 0.037 | -0.421 | -0.013 |
| Nursing home | 6.947 | 0.150 | 46.390 | 0.000 | 6.653 | 7.240 |
| Died in the year | 1.616 | 0.199 | 8.100 | 0.000 | 1.225 | 2.007 |
| Median household income (area) | 0.000 | 0.000 | -40.540 | 0.000 | 0.000 | 0.000 |
| Residents under poverty level | -0.019 | 0.007 | -2.840 | 0.005 | -0.033 | -0.006 |
| Number of hierarchical condition categories | 0.036 | 0.032 | 1.120 | 0.263 | -0.027 | 0.099 |
| Coronary artery disease | -0.736 | 0.183 | -4.020 | 0.000 | -1.095 | -0.377 |
| Congestive heart failure | -0.385 | 0.150 | -2.570 | 0.010 | -0.679 | -0.092 |
| Diabetes | -1.992 | 0.096 | -20.740 | 0.000 | -2.180 | -1.803 |
| Cancer | 1.052 | 0.140 | 7.540 | 0.000 | 0.779 | 1.326 |
| Chronic obstructive pulmonary disease | -1.654 | 0.128 | -12.880 | 0.000 | -1.906 | -1.402 |
| End stage renal disease | 0.472 | 0.424 | 1.110 | 0.265 | -0.359 | 1.303 |
| Midwest vs. Northeast | 4.507 | 0.114 | 39.610 | 0.000 | 4.284 | 4.730 |
| South vs. Northeast | 0.267 | 0.127 | 2.110 | 0.035 | 0.019 | 0.516 |
| West vs. Northeast | 12.552 | 0.135 | 92.970 | 0.000 | 12.287 | 12.817 |
| Constant | 18.857 | 0.604 | 31.200 | 0.000 | 17.672 | 20.042 |
| **Overall for Isolated** | **Margin** | **SE** | **Z Score** | **P-value** | **LL CI** | **UL CI** |
| Isolated | 9.418 | 0.058 | 161.010 | 0.000 | 9.303 | 9.532 |
| Small Town | 22.823 | 0.084 | 271.030 | 0.000 | 22.658 | 22.988 |
| Micropolitan | 30.933 | 0.083 | 372.720 | 0.000 | 30.770 | 31.096 |
| Metropolitan | 60.772 | 0.073 | 835.080 | 0.000 | 60.629 | 60.914 |

| **Distance small town beneficiaries to practice** |  |  |  |  |  |  |
| --- | --- | --- | --- | --- | --- | --- |
| N | 1,824,697 |  |  |  |  |  |
| R^2^ | 0.223 |  |  |  |  |  |
|  | **Coefficient** | **SE** | **Z Score** | **P-value** | **LL CI** | **UL CI** |
| Small Town vs. Isolated | -13.346 | 0.132 | -101.460 | 0.000 | -13.604 | -13.088 |
| Micropolitan vs. Isolated | 8.534 | 0.148 | 57.700 | 0.000 | 8.244 | 8.824 |
| Metropolitan vs. Isolated | 32.593 | 0.138 | 235.390 | 0.000 | 32.322 | 32.865 |
| Frail | 1.682 | 0.157 | 10.730 | 0.000 | 1.375 | 1.990 |
| Mean age | -0.067 | 0.004 | -15.010 | 0.000 | -0.076 | -0.059 |
| Under 65 | 0.021 | 0.142 | 0.150 | 0.885 | -0.257 | 0.298 |
| Over 85 | 0.683 | 0.123 | 5.560 | 0.000 | 0.443 | 0.924 |
| Female | -0.777 | 0.057 | -13.540 | 0.000 | -0.890 | -0.665 |
| White vs. Unknown | -0.420 | 0.285 | -1.470 | 0.141 | -0.979 | 0.140 |
| Black vs. Unknown | -1.464 | 0.309 | -4.730 | 0.000 | -2.070 | -0.858 |
| Other vs. Unknown | -0.571 | 0.492 | -1.160 | 0.246 | -1.535 | 0.393 |
| Asian vs. Unknown | 1.227 | 0.723 | 1.700 | 0.090 | -0.189 | 2.644 |
| Hispanic vs. Unknown | 0.842 | 0.427 | 1.970 | 0.049 | 0.005 | 1.680 |
| North American Native vs. Unknown | 17.214 | 0.375 | 45.880 | 0.000 | 16.479 | 17.950 |
| Disabled (original reason for Medicare eligibility) | -0.108 | 0.097 | -1.110 | 0.265 | -0.297 | 0.082 |
| Dual eligible for Medicaid | 0.632 | 0.079 | 7.980 | 0.000 | 0.477 | 0.788 |
| Nursing home | 7.192 | 0.114 | 63.170 | 0.000 | 6.969 | 7.415 |
| Died in the year | 1.748 | 0.152 | 11.540 | 0.000 | 1.451 | 2.045 |
| Median household income (area) | 0.000 | 0.000 | -31.560 | 0.000 | 0.000 | 0.000 |
| Residents under poverty level | 0.041 | 0.005 | 9.010 | 0.000 | 0.032 | 0.050 |
| Number of hierarchical condition categories | 0.154 | 0.024 | 6.340 | 0.000 | 0.107 | 0.202 |
| Coronary artery disease | -0.612 | 0.138 | -4.430 | 0.000 | -0.883 | -0.341 |
| Congestive heart failure | -0.501 | 0.114 | -4.400 | 0.000 | -0.724 | -0.277 |
| Diabetes | -1.680 | 0.073 | -23.060 | 0.000 | -1.822 | -1.537 |
| Cancer | 0.550 | 0.109 | 5.060 | 0.000 | 0.337 | 0.763 |
| Chronic obstructive pulmonary disease | -1.387 | 0.097 | -14.270 | 0.000 | -1.578 | -1.197 |
| End stage renal disease | 1.198 | 0.295 | 4.060 | 0.000 | 0.619 | 1.776 |
| Midwest vs. Northeast | 1.201 | 0.109 | 11.010 | 0.000 | 0.987 | 1.415 |
| South vs. Northeast | 1.188 | 0.111 | 10.730 | 0.000 | 0.971 | 1.405 |
| West vs. Northeast | 14.562 | 0.124 | 117.020 | 0.000 | 14.318 | 14.806 |
| Constant | 27.495 | 0.488 | 56.400 | 0.000 | 26.539 | 28.450 |
| **Distance for Small Town** | **Margin** | **SE** | **Z Score** | **P-value** | **LL CI** | **UL CI** |
| Isolated | 21.770 | 0.126 | 172.560 | 0.000 | 21.522 | 22.017 |
| Small Town | 8.424 | 0.037 | 225.900 | 0.000 | 8.350 | 8.497 |
| Micropolitan | 30.304 | 0.077 | 391.210 | 0.000 | 30.152 | 30.455 |
| Metropolitan | 54.363 | 0.056 | 964.200 | 0.000 | 54.252 | 54.473 |

| **Distance micropolitan beneficiaries to practice** |  |  |  |  |  |  |
| --- | --- | --- | --- | --- | --- | --- |
| N | 3,289,621 |  |  |  |  |  |
| R^2^ | 0.221 |  |  |  |  |  |
|  | **Coefficient** | **SE** | **Z Score** | **P-value** | **LL CI** | **UL CI** |
| Small Town vs. Isolated | 2.134 | 0.161 | 13.230 | 0.000 | 1.818 | 2.450 |
| Micropolitan vs. Isolated | -16.789 | 0.133 | -126.410 | 0.000 | -17.050 | -16.529 |
| Metropolitan vs. Isolated | 28.260 | 0.138 | 205.010 | 0.000 | 27.990 | 28.530 |
| Frail | 1.470 | 0.108 | 13.630 | 0.000 | 1.259 | 1.682 |
| Mean age | -0.066 | 0.003 | -21.110 | 0.000 | -0.072 | -0.060 |
| Under 65 | 0.183 | 0.100 | 1.830 | 0.067 | -0.013 | 0.379 |
| Over 85 | 0.831 | 0.086 | 9.630 | 0.000 | 0.662 | 1.000 |
| Female | -0.670 | 0.040 | -16.720 | 0.000 | -0.749 | -0.592 |
| White vs. Unknown | 0.225 | 0.192 | 1.170 | 0.241 | -0.152 | 0.602 |
| Black vs. Unknown | -0.242 | 0.209 | -1.160 | 0.247 | -0.653 | 0.168 |
| Other vs. Unknown | 1.019 | 0.311 | 3.280 | 0.001 | 0.409 | 1.629 |
| Asian vs. Unknown | 1.224 | 0.400 | 3.060 | 0.002 | 0.439 | 2.008 |
| Hispanic vs. Unknown | 0.400 | 0.268 | 1.490 | 0.136 | -0.126 | 0.926 |
| North American Native vs. Unknown | 8.779 | 0.277 | 31.650 | 0.000 | 8.236 | 9.323 |
| Disabled (original reason for Medicare eligibility) | 0.373 | 0.069 | 5.410 | 0.000 | 0.238 | 0.508 |
| Dual eligible for Medicaid | 1.319 | 0.057 | 23.190 | 0.000 | 1.207 | 1.430 |
| Nursing home | 7.649 | 0.083 | 92.540 | 0.000 | 7.487 | 7.811 |
| Died in the year | 1.825 | 0.108 | 16.980 | 0.000 | 1.615 | 2.036 |
| Median household income (area) | 0.000 | 0.000 | 27.650 | 0.000 | 0.000 | 0.000 |
| Residents under poverty level | 0.034 | 0.003 | 11.270 | 0.000 | 0.028 | 0.040 |
| Number of hierarchical condition categories | 0.177 | 0.017 | 10.600 | 0.000 | 0.144 | 0.209 |
| Coronary artery disease | -0.551 | 0.096 | -5.740 | 0.000 | -0.740 | -0.363 |
| Congestive heart failure | -0.730 | 0.080 | -9.150 | 0.000 | -0.886 | -0.573 |
| Diabetes | -1.529 | 0.051 | -30.130 | 0.000 | -1.628 | -1.430 |
| Cancer | 0.409 | 0.075 | 5.480 | 0.000 | 0.263 | 0.556 |
| Chronic obstructive pulmonary disease | -1.169 | 0.068 | -17.150 | 0.000 | -1.303 | -1.036 |
| End stage renal disease | 0.029 | 0.201 | 0.150 | 0.883 | -0.364 | 0.423 |
| Midwest vs. Northeast | 3.734 | 0.072 | 51.860 | 0.000 | 3.593 | 3.875 |
| South vs. Northeast | 5.825 | 0.070 | 83.120 | 0.000 | 5.687 | 5.962 |
| West vs. Northeast | 11.290 | 0.079 | 143.070 | 0.000 | 11.135 | 11.444 |
| Constant | 20.524 | 0.345 | 59.420 | 0.000 | 19.847 | 21.201 |
| **Distance for Micropolitan** | **Margin** | **SE** | **Z Score** | **P-value** | **LL CI** | **UL CI** |
| Isolated | 25.557 | 0.131 | 195.430 | 0.000 | 25.301 | 25.813 |
| Small Town | 27.691 | 0.095 | 291.180 | 0.000 | 27.505 | 27.878 |
| Micropolitan | 8.768 | 0.023 | 381.470 | 0.000 | 8.723 | 8.813 |
| Metropolitan | 53.817 | 0.043 | 1241.150 | 0.000 | 53.732 | 53.902 |

| **Distance metropolitan beneficiaries to practice** |  |  |  |  |  |  |
| --- | --- | --- | --- | --- | --- | --- |
| N | 20,204,872 |  |  |  |  |  |
| R^2^ | 0.041 |  |  |  |  |  |
|  | **Coefficient** | **SE** | **Z Score** | **P-value** | **LL CI** | **UL CI** |
| Small Town vs. Isolated | -9.314 | 0.144 | -64.700 | 0.000 | -9.596 | -9.032 |
| Micropolitan vs. Isolated | -4.161 | 0.136 | -30.620 | 0.000 | -4.427 | -3.894 |
| Metropolitan vs. Isolated | -35.818 | 0.124 | -287.860 | 0.000 | -36.062 | -35.574 |
| Frail | 0.872 | 0.036 | 24.020 | 0.000 | 0.800 | 0.943 |
| Mean age | -0.090 | 0.001 | -75.030 | 0.000 | -0.093 | -0.088 |
| Under 65 | 0.133 | 0.041 | 3.220 | 0.001 | 0.052 | 0.215 |
| Over 85 | 1.121 | 0.032 | 35.170 | 0.000 | 1.059 | 1.184 |
| Female | -0.610 | 0.015 | -40.080 | 0.000 | -0.640 | -0.580 |
| White vs. Unknown | 0.504 | 0.059 | 8.550 | 0.000 | 0.388 | 0.620 |
| Black vs. Unknown | 0.200 | 0.064 | 3.140 | 0.002 | 0.075 | 0.325 |
| Other vs. Unknown | -0.108 | 0.082 | -1.310 | 0.190 | -0.270 | 0.053 |
| Asian vs. Unknown | -1.006 | 0.079 | -12.800 | 0.000 | -1.160 | -0.852 |
| Hispanic vs. Unknown | -0.697 | 0.079 | -8.800 | 0.000 | -0.853 | -0.542 |
| North American Native vs. Unknown | 4.866 | 0.150 | 32.550 | 0.000 | 4.573 | 5.159 |
| Disabled (original reason for Medicare eligibility) | 0.920 | 0.029 | 31.580 | 0.000 | 0.863 | 0.978 |
| Dual eligible for Medicaid | 0.839 | 0.023 | 36.620 | 0.000 | 0.795 | 0.884 |
| Nursing home | 11.148 | 0.032 | 353.640 | 0.000 | 11.086 | 11.209 |
| Died in the year | 3.455 | 0.042 | 82.200 | 0.000 | 3.373 | 3.538 |
| Median household income (area) | 0.000 | 0.000 | -2.000 | 0.046 | 0.000 | 0.000 |
| Residents under poverty level | -0.038 | 0.001 | -36.890 | 0.000 | -0.040 | -0.036 |
| Number of hierarchical condition categories | 0.057 | 0.006 | 9.340 | 0.000 | 0.045 | 0.069 |
| Coronary artery disease | -0.754 | 0.037 | -20.340 | 0.000 | -0.827 | -0.681 |
| Congestive heart failure | -0.342 | 0.030 | -11.280 | 0.000 | -0.402 | -0.283 |
| Diabetes | -1.171 | 0.020 | -60.030 | 0.000 | -1.210 | -1.133 |
| Cancer | 0.262 | 0.026 | 9.920 | 0.000 | 0.210 | 0.314 |
| Chronic obstructive pulmonary disease | -1.035 | 0.028 | -37.270 | 0.000 | -1.089 | -0.980 |
| End stage renal disease | 0.552 | 0.069 | 8.020 | 0.000 | 0.417 | 0.687 |
| Midwest vs. Northeast | 1.673 | 0.023 | 72.030 | 0.000 | 1.628 | 1.719 |
| South vs. Northeast | 4.926 | 0.020 | 240.770 | 0.000 | 4.886 | 4.966 |
| West vs. Northeast | 4.685 | 0.024 | 195.360 | 0.000 | 4.638 | 4.732 |
| Constant | 50.462 | 0.164 | 308.370 | 0.000 | 50.141 | 50.783 |
| **Distance for Metropolitan** | **Margin** | **SE** | **Z Score** | **P-value** | **LL CI** | **UL CI** |
| Isolated | 48.036 | 0.124 | 386.780 | 0.000 | 47.792 | 48.279 |
| Small Town | 38.722 | 0.073 | 528.560 | 0.000 | 38.578 | 38.865 |
| Micropolitan | 43.875 | 0.056 | 789.650 | 0.000 | 43.766 | 43.984 |
| Metropolitan | 12.217 | 0.008 | 1617.360 | 0.000 | 12.202 | 12.232 |

| **Unadjusted Bypass Numbers** |  |  |  |  |  |
| --- | --- | --- | --- | --- | --- |
|  |  | **Practice Location** | | | |
| **Beneficiary Location** | Overall | **Isolated** | **Small Town** | **Micropolitan** | **Metropolitan** |
| **Isolated** |  |  |  |  |  |
| N | 1,302,108 | 487,876 | 231,996 | 241,595 | 340,641 |
| % | 100% | 37.47 | 17.82 | 18.55 | 26.16 |
| Distance, miles | 29 | 9 | 23 | 31 | 61 |
| **Small Town** |  |  |  |  |  |
| N | 1,888,217 | 93,527 | 1,056,052 | 246,965 | 491,673 |
| % | 100% | 4.95 | 55.93 | 13.08 | 26.04 |
| Distance, miles | 23 | 22 | 8 | 30 | 55 |
| **Micropolitan** |  |  |  |  |  |
| N | 3,464,931 | 78,348 | 147,352 | 2,490,234 | 748,997 |
| % | 100% | 2.26 | 4.25 | 71.87 | 21.62 |
| Distance, miles | 19 | 25 | 28 | 9 | 54 |
| **Metropolitan** |  |  |  |  |  |
| N | 21,042,874 | 83,039 | 225,989 | 404,175 | 20,329,762 |
| % | 100% | 0.39 | 1.07 | 1.92 | 96.1 |
| Distance, miles | 13 | 49 | 40 | 45 | 12 |

| **Unadjusted Outcomes** | **Practice Location** | | | |
| --- | --- | --- | --- | --- |
|  | **Isolated** | **Small Town** | **Micropolitan** | **Metropolitan** |
| N | 743,196 | 1,662,480 | 3,384,913 | 21,926,378 |
| Inpatient stays |  |  |  |  |
| Mean number of stays per beneficiary | 0.296 | 0.304 | 0.296 | 0.310 |
| % with a multiple stays | 6.260% | 6.416% | 6.177% | 6.498% |
| % with a potentially avoidable hospitalizations, acute | 2.141% | 2.183% | 1.776% | 1.649% |
| % with a potentially avoidable hospitalizations, chronic | 2.678% | 2.814% | 2.717% | 2.678% |
| Readmissions |  |  |  |  |
| % 30-day all-cause for medical discharges | 19.675% | 19.510% | 17.587% | 18.173% |
| % 30-day all-cause for surgical discharges | 12.212% | 21.775% | 11.918% | 12.456% |
| % 30-day all-cause for acute myocardial infarction discharges | 15.746% | 15.915% | 14.396% | 15.819% |
| % 30-day all-cause for congestive heart failure | 24.490% | 24.558% | 23.077% | 23.400% |
| % 30-day all-cause for pneumonia discharges | 20.849% | 19.615% | 17.505% | 17.997% |
| Emergency department visits |  |  |  |  |
| Mean number of visits per beneficiary discharged | 0.563 | 0.615 | 0.565 | 0.456 |
| % with multiple visits discharged | 15.535% | 16.924% | 16.554% | 14.945% |
| Mean % visits that were |  |  |  |  |
| Necessary, but preventable | 10.812% | 10.877% | 10.256% | 9.943% |
| Unnecessary, but emergent | 33.898% | 34.369% | 33.790% | 32.406% |
| Unnecessary and nonemergent | 25.041% | 25.126% | 25.074% | 25.174% |
| % died | 4.180% | 4.196% | 3.949% | 3.888% |
| Quality Metrics |  |  |  |  |
| % diabetics who had a blood lipids test | 72.135% | 73.929% | 75.440% | 77.359% |
| % diabetics who had an eye exam | 65.138% | 64.811% | 66.733% | 69.314% |
| % diabetics who had a hemoglobin A1c test | 85.082% | 85.997% | 86.346% | 85.982% |
| % mammogram, aged 50-74 | 58.562% | 59.647% | 63.045% | 65.795% |
| **Access Measures** |  |  |  |  |
| % primary care clinician visit within 14-days of stay | 65.598% | 64.346% | 61.630% | 58.337% |
| % follow-up within 30-days of mental health stay | 70.195% | 69.910% | 70.974% | 67.934% |
| % follow-up within 7-days of mental health stay | 38.380% | 37.813% | 38.226% | 39.096% |
| Mean number of visits | 10.312 | 10.122 | 10.577 | 11.457 |
| Family medicine | 2.818 | 2.728 | 2.301 | 1.752 |
| Internist | 0.837 | 1.039 | 1.471 | 2.047 |
| Geriatrician | 0.014 | 0.012 | 0.014 | 0.064 |
| Other primary care | 0.163 | 0.183 | 0.107 | 0.107 |
| Specialist | 3.336 | 3.468 | 3.989 | 5.183 |
| Nurse practitioner, physician assistant, clinical nurse specialist | 2.298 | 1.899 | 2.033 | 1.648 |
| Mean number of clinicians encountered |  |  |  |  |
| Primary care physician | 0.969 | 1.047 | 1.089 | 1.229 |
| Specialist | 1.568 | 1.649 | 1.850 | 2.391 |
| Nurse practitioner, physician assistant, clinical nurse specialist | 0.879 | 0.790 | 0.886 | 0.739 |
| **Payments, $** |  |  |  |  |
| Total | 9,615 | 9,714 | 9,645 | 11,133 |
| Acute care | 3,092 | 3,092 | 3,107 | 3,551 |
| Procedures | 1,291 | 1,340 | 1,415 | 1,634 |
| Evaluation and management | 930 | 999 | 1,090 | 1,391 |
| Other | 3,646 | 3,773 | 3,736 | 4,304 |
